# Advancing energy storage and supercapacitor applications through the development of Li^+^-doped MgTiO_3_ perovskite nano-ceramics

**DOI:** 10.1038/s41598-024-52262-6

**Published:** 2024-01-22

**Authors:** Hend S. Magar, A. M. Mansour, Ali B. Abou Hammad

**Affiliations:** 1 Applied Organic Chemistry Department, National Research Centre (NRC), 33 El‑Bohouth St., Dokki, 12622 Cairo Egypt; 2 Solid State Physics Department, Physics Research Institute, National Research Centre, 33 El Bohouth St., Dokki, 12622 Giza Egypt

**Keywords:** Materials science, Condensed-matter physics, Electronic properties and materials

## Abstract

Perovskite oxide materials, specifically MgTiO_3_ (MT) and Li-doped MgTiO_3_ (MTxLi), were synthesized via a sol–gel method and calcination at 800 °C. This study explores the impact of varying Li doping levels (x = 0, 0.01, 0.05, and 0.1) on the crystalline structure and properties of MgTiO_3_. X-ray diffraction analysis revealed a well-defined rhombohedral MgTiO_3_ phase. Optical diffuse reflectance measurements provided insights into energy gap values, refractive index, and dielectric constant. Li^+^ doping enhanced the electrical properties of MgTiO_3_, with a notable phase transition observed at 50 °C. The study investigated impedance and AC conductivity under varying temperature and frequency conditions (25–120 °C, 4 Hz to 8 MHz). Electrochemical analysis through cyclic voltammetry and electrochemical impedance spectroscopy confirmed highly electrocatalytic properties for MTxLi, particularly when modified onto screen-printed electrodes. This work not only advances the understanding of Li-doped MgTiO_3_ nanostructures but also highlights their significant potential for direct electrochemical applications, particularly in the realm of energy storage.

## Introduction

The increasing demand for energy storage and consumption has prompted scientists to search for novel materials that can be applied in both energy storage and energy conversion technologies. Manufacturing energy devices, such as supercapacitors and batteries, entails materials with a diversity of electrical, chemical, and mechanical properties that can endure various environmental conditions and high temperatures in a sustainable manner. Ceramic oxides have been constructed with the desired properties to fulfill the manufacturing requirements. Ceramic oxides are characterized by their durability and corrosion resistance. Therefore, it widely used in energy harvesters, microwave communications, optical communication, photoelectrochemical devices, electro-optic, fuel cells, batteries, and sensors^1–3^. Among the wide range of ceramics, perovskite titanates have emerged as highly promising materials that have been extensively investigated for practical applications in recent decades.

Perovskite oxides have garnered substantial attention in recent years due to their diverse and exceptional properties, making them compelling candidates for various applications, especially in the realm of energy storage technology. This class of materials exhibits a distinctive crystal structure characterized by the general formula ABX_3_, where A is typically an alkaline earth metal, B is a transition metal, and X is an anion^4^.

In the context of perovskite oxides, alkaline earth-based titanates, particularly those derived from barium (Ba) and strontium (Sr), have emerged as pivotal contributors to advancements in energy storage technologies. The unique combination of their crystal structure and electrochemical properties makes them promising candidates for applications such as supercapacitor electrodes^4–6^.

The MTiO_3_ series, which includes elements like Mg, Mn, Ni, and others, is of particular interest due to its exceptional dielectric constant and remarkably high-quality factor. These unique properties in perovskite titanates stem from the arrangement of TiO_6_ octahedra, which are isolated by MO_6_ octahedra and cation vacancies. Each layer of MO_6_ octahedra is situated between two layers of TiO_6_ octahedra, contributing to these distinctive characteristics^3,7,8^.

Perovskite titanates find applications in various fields, such as optoelectronics, and lithium-ion batteries, gas sensing, among others. The thermal stability of the perovskite MTiO_3_ is a major factor that affects the properties of the perovskite, where some perovskite decomposes into spinel structures and the rutile phase^9,10^. MgTiO_3_ is a highly thermally stable perovskite with a high tolerance factor; it possesses good mechanical resistance and stability even in corrosive environments^11–13^. MgTiO_3_ demonstrates exceptional characteristics, including a high-quality factor (Q), notable dielectric constant, minimal dielectric loss, low leakage current density, and robust high-temperature stability. These attributes play a pivotal role in advancing the fields of microwave and millimeter wave integrated circuits, global telecommunication technology, integrated optical devices, and dynamic random-access memories^12^.

The utilization of MgTiO_3_ extends across various domains, contingent upon the specific modifiers employed. When MgTiO_3_ is modified with rare earth metals, its applications encompass a wide range of areas, including light-emitting and photovoltaic applications, plasma and flat panel devices, light-emitting and solid-state diodes, and optical devices, among others^13,14^. Meanwhile, MgTiO_3_ modified with transition metal ions can be used in microwave, satellite, and terrestrial communication, including radio software, GPS, and DBSTV for environmental monitoring^15^.

The high daily energy consumption drives the scientific community to explore new materials for application in energy storage and energy conversion. Perovskite oxides and halides belong to the prospective materials that can replace conventional materials for energy applications. The demands for new materials and the development of novel devices for the different energy applications push to fabricate perovskite materials at the nanoscale and develop their structure. The supercapacitor stands as a forward-looking energy storage device, capable of storing a significant electric capacitance within a compact arrangement. It finds extensive application in various electronic devices^16–18^. This technology boasts several benefits, including its adaptability to a wide range of operating temperatures, a limitless cycle life, a straightforward charging and discharging circuit, rapid charging capabilities, and a cost-effective nature.

Perovskite materials at the nanoscale exhibit distinctive features, including extensive porous structures, a significant surface area, regulated transport and charge-carrier mobility, potent absorption, and photoluminescence. Additionally, their unique adaptability in terms of composition, morphology, and functionalities candidate perovskite nanocrystals as highly effective elements for energy applications such as photovoltaics, catalysis, thermoelectrics, batteries, supercapacitors, and hydrogen storage systems^19–22^.

The electrochemical performance of supercapacitors depends on electrode materials, electrolytes, and potential windows. Metal oxides are extensively employed in energy storage and conversion applications, mainly due to their cost-effectiveness, abundant availability, ease of preparation, multiple valence states, and environmental friendliness. They find applications in various fields, including sensors^23–26^, biosensors^27,28^, lithium batteries^29^, supercapacitors^30–32^, electrocatalysis, and fuel cells.

The current work aims to fabricate MgTiO_3_ modified with Li^+^ to extend their application in energy storage systems, including lithium-ion batteries and supercapacitors. The production of Li-MgTiO_3_ as a dielectric nanoceramic material for supercapacitors was achieved via the acetic acid sol–gel method, followed by 3-h calcination at 800 °C to promote crystalline development. This research explores into evaluating the electrical and optical attributes of the resultant Li-MgTiO_3_ perovskite nano-ceramics, encompassing properties such as impedance, Cole–Cole plot analysis, conductivity, absorbance, and energy band gap.

The electrochemical studies were produced by using impedance and cyclic voltammetry electrochemical techniques. The modified screen-printed electrode exhibited remarkably electrocatalytic properties, proving effective in direct electrochemical applications. Notably, this synthesis approach holds significance for advancing energy storage applications.

This study ensures a comprehensive exploration of the doping mechanisms, contributing valuable insights into the tailored design of titanate-based materials for enhanced energy storage applications.

## Experimental

### ***Constructions of MgTiO***_***3***_*** and Mg***_***(1-x)***_***Li***_***x***_***TiO***_***3***_

Initially, the synthesis of MgTiO_3_ (MT) was carried out using the sol–gel reaction method. All the necessary chemicals were procured from Sigma Aldrich. The procedure commenced by dissolving precise amounts of highly pure magnesium acetate (Mg(CH_3_COO)_2_•4H_2_O) in 15 mL of water and acetic acid with continuous stirring. The required stoichiometric quantities of titanium isopropoxide were dissolved in acetylacetone (CH_3_COCH_2_COCH_3_) and introduced into the previously mentioned solution while maintaining a temperature of 50 °C.

**Figure 1:**
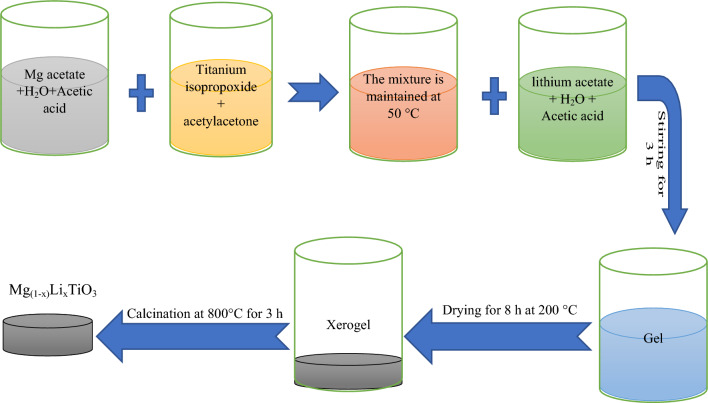
Schematic diagram of the synthesis process.

To produce Mg_(1-x)_Li_x_TiO_3_ (MTxLi), lithium acetate was dissolved in acetic acid and distilled water and subsequently combined with the MT solution, Fig. 1. This process led to the formation of the desired chemical structure, Mg_(1-x)_Li_x_TiO_3_, with varying lithium content (x = 0, 0.01, 0.05, & 0.1 mol.%, Table 1). The combination was stirred by magnetic stirring for 3 h. Afterward, all gel systems were subjected to drying at 200 °C for 8 h. The resulting xerogels were exposed to calcination at 800 °C for 3 h in the air.

**Table 1:** Sample composition and abbreviation.

Sample abbreviation	Mg	Li	Ti	Chemical formula
MT	1	0	1	MgTiO_3_
MT1Li	0.99	0.01	1	Mg_0.99_Li_0.01_TiO_3_
MT5Li	0.95	0.05	1	Mg_0.95_Li_0.05_TiO_3_
MT10Li	0.9	0.1	1	Mg_0.9_Li_0.1_TiO_3_

### Characterization

#### Crystalline phase

The structural phases of the samples were determined through Rigaku X-ray diffraction (D-max 2500), utilizing monochromatic (Cu Ka) radiation. The settings used were an acceleration voltage of 40 kV and an applied current of 100 mA.

#### Scanning electron microscopy (SEM)

Surface morphology was examined using a scanning electron microscope (SEM)—specifically, the Quanta FEG-250 from the Czech Republic.

#### Optical properties

UV–visible diffuse reflectance spectroscopy (DRS) assessments were conducted using a Jasco V570 UV–vis NIR spectrophotometer equipped with an integration sphere diffuse reflectance accessory, originating from the USA.

#### Electrical properties measurements

The ac conductivity and the impedance (Z' and Z") of MT and MTxLi were determined by utilizing the Hioki LCR IM3536. The sample powders were compacted into tablets with a diameter of 13 mm and a thickness defined as d and sintered at 800 °C for 3 h. The measurements were conducted employing the parallel plate capacitor methodology. The measurements were carried out in the frequency range (ν = 4Hz to 8MHz) and temperature range (T = 25 to 120 °C).

The expression for the complex impedance is provided in Eq. (1):

1 $$  Z* \, = \, Z^{\prime} \, - j \, Z, j = \, \surd \left( { - 1} \right) $$

The real (Z') and imaginary (Z") impedance of the MT and MTxLi samples were determined using Eq. (2) and Eq. (3), respectively^33^.

2 $${Z}^{\prime}= \frac{d}{2\pi \nu A{\varepsilon }_{0}}\times \frac{\varepsilon^{{\prime\prime}}(\nu )}{{\left({\varepsilon }^{\prime}\left(\nu \right)\right)}^{2}+{({\varepsilon }^{{\prime\prime}}\left(\nu \right))}^{2}}$$

3 $${Z}^{{\prime\prime}}= \frac{d}{2\pi \nu A{\varepsilon }_{0}}\times \frac{\varepsilon^{\prime}(\nu )}{{\left({\varepsilon }^{\prime}\left(\nu \right)\right)}^{2}+{({\varepsilon }^{{\prime\prime}}\left(\nu \right))}^{2}}$$

Here A represents the electrode surface area, ε_0_ denotes the permittivity of the free space (8.854 × 10^–12^ F m^−1^), ε′(*ν*) and ε′′(*ν*) stand for the dielectric constant and dielectric loss of the samples, respectively^7,34^.

4 $${\varepsilon }^{\prime}\left(\nu \right)= \frac{C \times d}{{\varepsilon }_{0} \times A} \mathrm{ and }{\varepsilon }^{"}\left(\nu \right)= {\varepsilon }^{\prime}\left(\nu \right) \times {\text{tan}}\delta $$

(C and tan δ are the measured capacitance loss tangent factor, respectively).

The ac conductivity is given by

5 $$\sigma (\nu )=G(\nu ) \times \frac{d}{A}$$

### Electrochemical measurements

Potassium chloride, potassium ferricyanide and potassium ferrocyanide were brought from sigma Aldrich.

**Figure 2:**
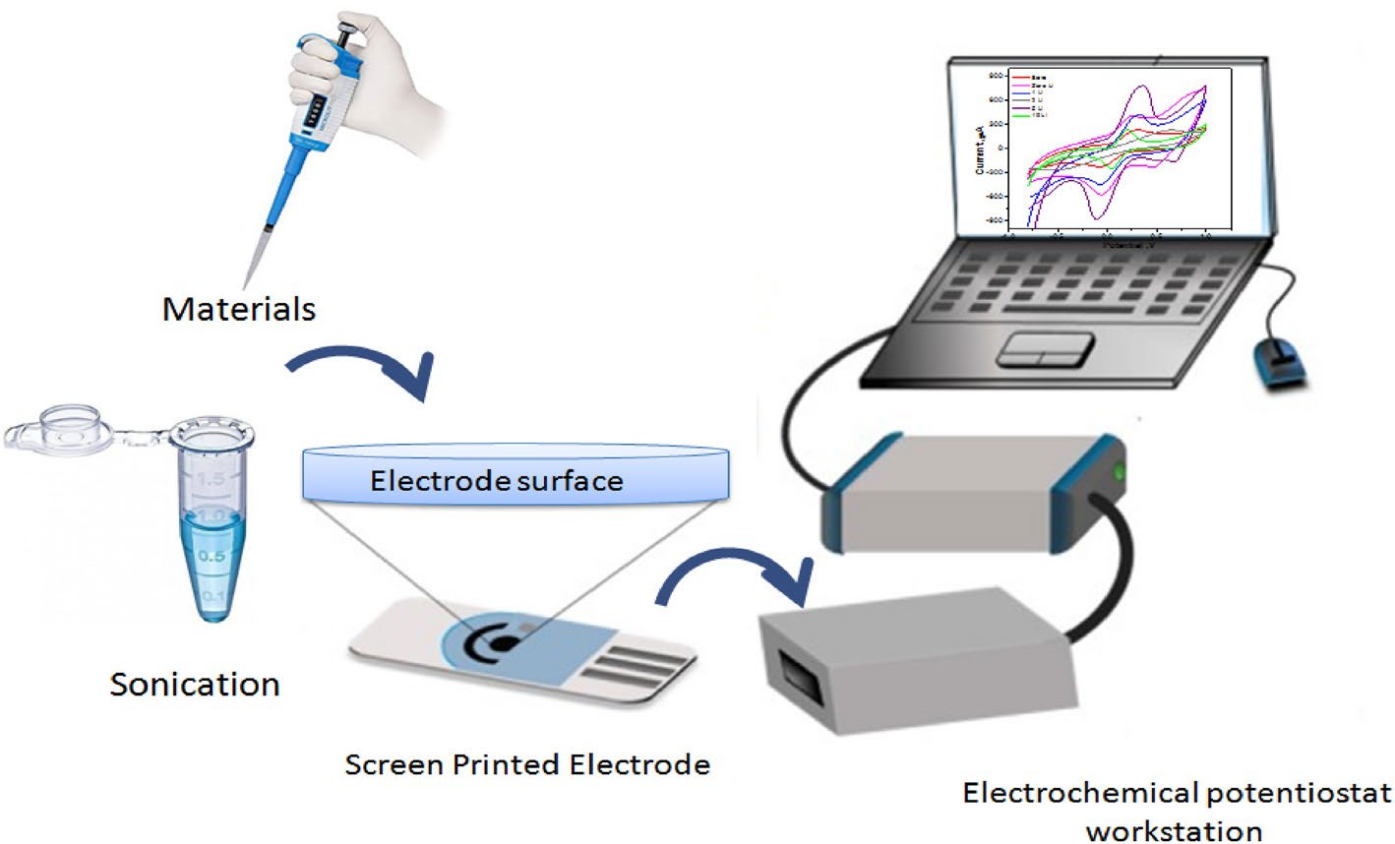
schematic diagram illustrates the modification steps of SPEs with the MTxLi and their electrochemical study using a potentiostat.

Electrochemical studies were produced using electrochemical workstation CHI –potentiostat and screen-printed electrodes (SPEs). For modified SPEs preparation, 10.0 mg of MT or MTxLi was weight, dispersed (1 ml double distilled water) and sonicated for 30 min. Furthermore, 30 μl of sonicated solution were drop on the SPE surface and dry in air. For CV and EIS measurements a mixture solution of 5 mM of the ferri/ferrocyanide [Fe (CN)_6_]^3−/4−^ and 0.1 M KCl are used. The following schematic shows the prepared materials SPEs modification method for electrochemical performance measurements (Fig. 2).

## Result and discussion

### Crystalline phase (XRD)

**Figure 3:**
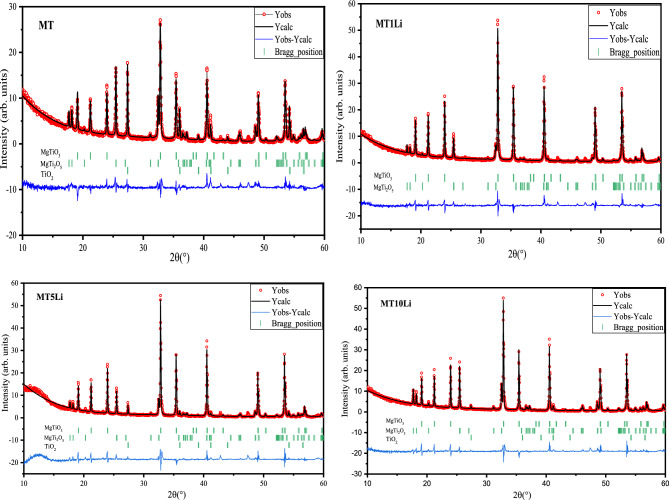
the XRD chart of MgTiO_3_ doped with different concentration of Li^+^ perovskite nano-ceramics.

X-ray diffraction stands out as the most valuable method for discerning the crystalline characteristics and phase purity of a sample. Additionally, it facilitates the determination of essential structural parameters like crystallite phase, crystallite size, lattice parameters…etc.^35^. Figure 3 displays the Rietveld refinement of the XRD pattern of both MT and Li-doped MT samples. The presence of distinct and intense peaks in the XRD pattern affirms the elevated structural organization and enhanced crystalline quality of the samples. The Rietveld refinement proves that both MT and Li-doped MT exhibit distinct crystallographic planes that are indexed to the rhombohedral crystal structure MgTiO_3_ Rhombohedral, alongside some peaks indexed to a secondary phase (orthorhombic MgTi_2_O_5_ and Tetragonal TiO_2_). The Rietveld refinement results are tabulated in Table 2, Lattice parameters and the fractions of the different phases of the compositions.

**Table 2:** Phase fraction and lattice parameter of each phase obtained from the Rietveld refinement of the samples.

	MgTiO_3_ Trigonal (R-3)			MgTi_2_O_5_ Orthorhombic (Bbmm)				TiO_2_ Tetragonal (P42/mnm)		
	Fraction	(a = b) Ǻ	(c) Ǻ	Fraction	(a) Ǻ	(b) Ǻ	(c) Ǻ	Fraction	(a = b) Ǻ	(c) Ǻ
MT	60.82%	5.06516	13.9368	26.02%	9.76338	10.0302	3.74624	13.16%	4.60328	2.96557
Mg1Li	88.71%	5.0664	13.9338	11.29%	9.7525	10.0259	3.7478	–	–	–
Mg5Li	85.03%	5.06639	13.9346	12.69%	9.73744	10.0182	3.74527	2.28%	4.60448	2.96601
Mg10Li	73.41%	5.06511	13.9292	25.96%	9.73674	10.0177	3.74601	0.63%	4.60373	2.96662

The appearance of the impurity phase MgTi_2_O_5_ can be attributed to the decomposition of MgTiO_3_, which is likely caused by the volatilization of Mg and oxygen deficiency^36,37^.

6 $$ {\text{2MgTiO}}_{{3}} \leftrightarrow {\text{ MgTi}}_{{2}} {\text{O}}_{{5}} + {\text{MgO}} $$

The Rietveld refinement doesn’t show a secondary phase for Lithium oxide, and according to Hume-Rothery criteria, the difference between the ionic radii of Li^+^ (0.74 Å) and Mg^2+^ (0.72 Å) is less than 10% therefore, Li^+^ is best suited to replace Mg^2+^ in the perovskite structure^38^.

The XRD chart provides evidence that the incorporation of Li^+^ ions enhance the crystal structure of the samples. As the Li^+^ ion concentration increases, the peak intensity of the primary phase, MgTiO_3_, shows an upward trend. In contrast, the peak intensity of the secondary phase declines, and at elevated Li^+^ ion concentrations, certain peaks related to the secondary phase disappear entirely. Consequently, Li^+^ ions emerge as a suitable choice for altering the local crystal structure of MgTiO_3_ since they function as charge compensators^39^.

### ***Li***^+^***- MgTiO***_***3***_*** perovskite nano-ceramics morphology (SEM)***

**Figure 4:**
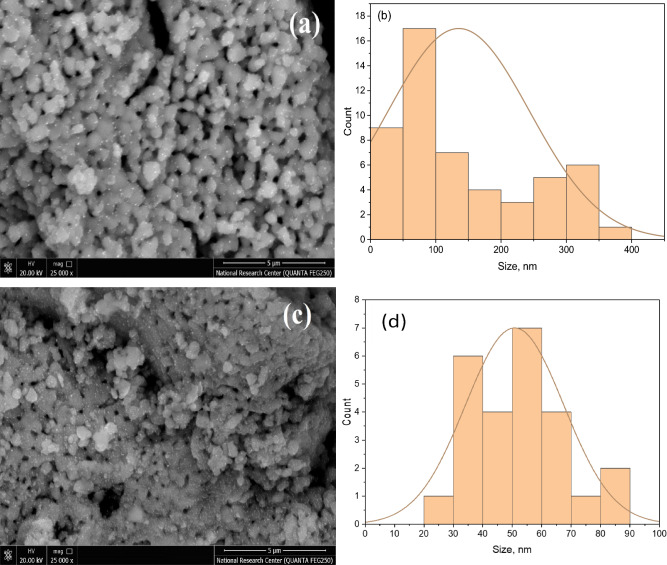
SEM micrographs and particle size distribution of MT (**a** and **b**) and MT10Li (**c** and **d**), respectively.

Figure 4a and c displays SEM micrographs of MT and MT10Li nanopowders that have undergone calcination at a temperature of 800 °C for 3 h, at magnification 25X. The micrographs of the Li^+^-MgTiO_3_ nanopowders exhibit significant regular formation due to the presence of an interconnected network structure and higher surface energy. Upon calcination at 800 °C, the particles grow to sizes ranging from approximately 38 to 62 nm, Fig. 4b and d, displaying a good-particles distribution. The SEM micrographs distinctly reveal the presence of many nanopores within the perovskite nanoceramics. These findings suggest that the process of calcination at 800 °C leads to increased particle sizes and reduced agglomeration tendencies among the Li^+^-MgTiO_3_ nano powders.

### Optical properties

UV–Vis spectroscopy is dedicated to assessing the light absorption capabilities of a chemical system. Within UV–VIS spectroscopy, molecules absorb incident light, leading to the excitation of electrons from their ground state to a higher energy level^40^. The energy of the absorbed light matches the energy gap between these ground and higher energy states. The spectrophotometer is used to measure the diffuse reflectance (Rd) of the sample as a function of the wavelength. Using these data, the energy band gap of a semiconductor can be determined^41^.

**Figure 5:**
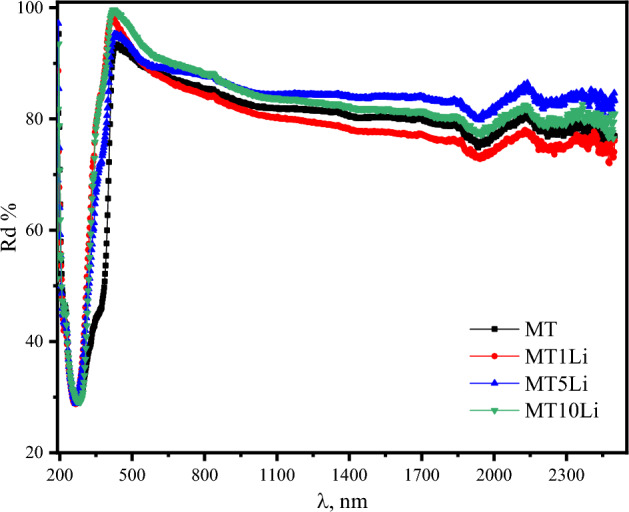
The diffuse reflectance spectra of the prepared samples at room temperature.

Figure 5 presents the diffuse reflectance spectra of the prepared samples at room temperature. The samples demonstrate a high reflectance at the measuring start at about 190 nm and then drops to minimum values at about 275 nm. At wavelength 275 nm, the diffuse reflectance shows a sharp increase with increasing of wavelength and reaches a maximum value at about 430 nm for all samples. A slight decrease is observed with increasing of wavelength until the end of measuring range. Moreover, the presence of interference peaks becomes evident at higher wavelengths.

The decrease in diffuse reflectance and the absence of fringes at shorter wavelengths signify the fundamental absorption of the films. The addition of Li^+^ causes a blue shift in the absorption edge, indicating an increase in oxygen content and highlighting the influence of oxygen on the optical properties^42^.

**Figure 6:**
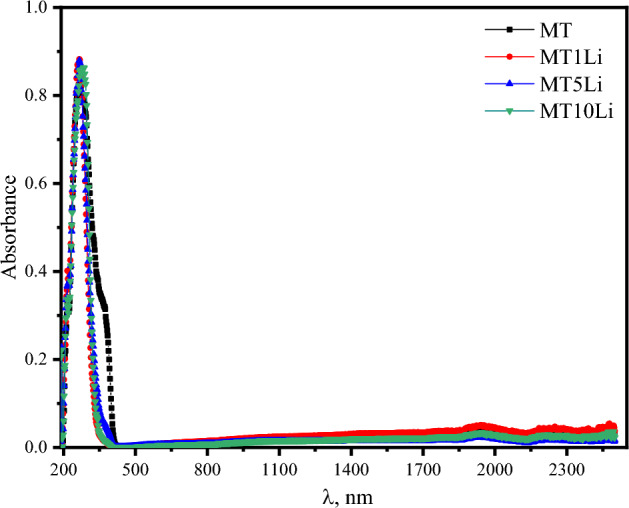
The absorbance spectra exhibited by the prepared perovskite nano-ceramics.

Figure 6 provides a visual representation of the absorbance spectra exhibited by the prepared samples. Notably, an absorption peak of considerable magnitude manifests itself at approximately 267 nm. This significant peak can be attributed to a charge transfer transition, specifically from O^2−^ to Ti^4+^^43^. For titanates, the presence of defect titanate centers exhibiting absorption beyond the intrinsic absorption edge is a well-known phenomenon^44^. Notably, a subtle disorder between Mg^2+^ and Ti^4+^ in MgTiO_3_ gives rise to the formation of titanate centers, which play a pivotal role in shaping the tail observed in the reflection spectrum of MgTiO_3_^43^. The hollandite structure exhibited a similar phenomenon in the titanate K_1.8_Mg_0.9_Ti_7.1_O_16_, as observed by de Haart et al.^45^. In MgTiO_3_, the defect titanate center resides within the Mg sublattice of the ilmenite structure. This particular titanate center may serve as a recombination center, resulting in the absence of photocurrent when excited in the defect titanate centers^43^.

The Kubelka–Munk function, also known as the Kubelka–Munk relation, precisely correlates the reflectance spectrum with the absorbance of materials denoted as F(R∞)^46^. The Kubelka–Munk relation^40^ allows estimation of the band gap type and value:

7 $$F\left({R}_{\infty }\right)=C{(h\nu -{E}_{g})}^{n}/h\nu $$

**Figure 7:**
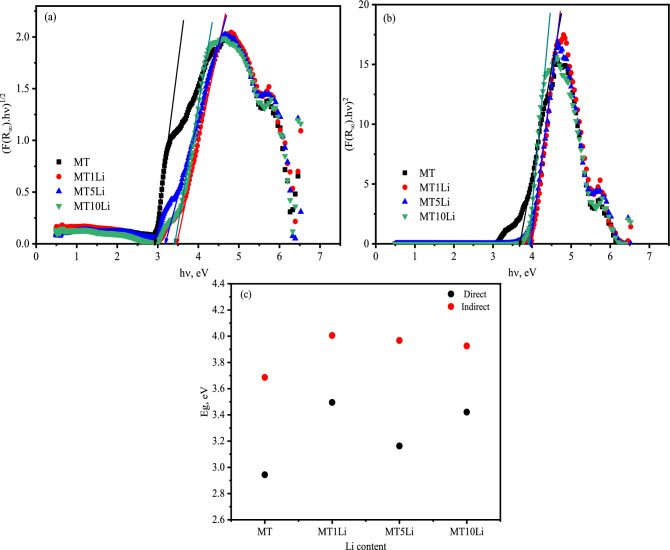
a & b are the (αhʋ)1/2, (αhʋ)2 curve versus (hʋ), and c is the energy gap against Li^+^ concentrations.

The Kubelka–Munk formula, denoted as F(R∞), employs photon energy (hυ) and the optical band gap (Eg) to calculate. The value of "n" is determined by the nature of the electronic transition and can be either 1/2 or 2 for directly allowed transitions or indirectly allowed transitions, respectively^41^. Figure 7a,b display the graphical representations of (αhʋ)^1/2^ and (αhʋ)^2^ as functions of (hʋ) with the goal of identifying the type and magnitude of the gap transition. The current analysis accommodates both direct and indirect transition types, with indirect transitions exhibiting higher values, as depicted in Fig. 7c. Consequently, a direct transition holds a higher likelihood than an indirect transition.

The energy of the band gap experiences a sudden increase with the addition of Li, followed by a slight decrease (Fig. 7c). The addition of Li led to a short-range ordering (SRO) mechanisms and disorder^47^. Bernard et al.^47^ discovered a novel phase (Li_2_MgTiO_4_) within the structure of rock salt, featuring a Mg/Ti ratio of 1, induced by the presence of lithium. These phenomena are attributed to short-range ordering (SRO) mechanisms and disorder. Their research on various samples reveals that these phenomena are associated with alterations in the cationic composition and phase transitions. The existence of SRO and disorder mechanisms within the structure of rock salt, in conjunction with the phase transitions, corresponds with the capacity of both lithium (Li) and magnesium (Mg) to coexist in the same location, particularly the tetrahedral sites that are theoretically unoccupied in a cubic face-centered system adhering to the NaCl-type structure^47^.

The inverse relationship between the energy gap and refractive index of a material is well-documented, where an increase in the energy gap leads to a corresponding decrease in the refractive index^48^. Thus, it is postulated that a distinct relationship exists between these commonly observed variables. To elucidate this connection, numerous endeavors have been undertaken to establish an empirical or semiempirical relation between the refractive index of semiconductors and their energy gaps^49^. These suggested relations, supported by compelling arguments and aligned with experimental data, serve as a valuable framework for understanding the intricate interplay between the refractive index and energy gap in semiconducting materials.

From these relations, the relations created by Kumar and Singh's^50^:

7 $${n}_{Kumar}=K{E}_{g}^{C}$$

where K = 3.37 and C = -0.32. Utilizing the Kumar and Singh method, the alteration in refractive index with the addition of Li has been computed for both direct and indirect transitions. These calculations have been documented in Table 3, offering the behavior of the refractive reverse to the band gap energy.

**Table 3:** Energy gap; refractive index; and dielectric constant of the prepared samples for both direct and indirect.

Li content	E_g direct_ (eV)	E_g indirect_ (eV)	n_Kumar_ (d)	n_Kumar_ (in)	e_Kumar_ (d)	e_Kumar_ (in)
MT	2.943	3.686	2.37	2.21	5.65	4.88
MT1Li	3.495	4.006	2.24	2.15	5.06	4.63
MT5Li	3.163	3.967	2.32	2.16	5.39	4.66
MT10Li	3.421	3.926	2.26	2.17	5.12	4.69

The dielectric constants of the established samples, pertaining to both direct and indirect gap types, have been diligently calculated from the refractive index using Eq. ^51^.

$${\varepsilon }_{\infty }={n}^{2}$$

These calculations are meticulously presented in Table 3. Notably, the variation in dielectric constant with changes in Li content closely mirrors the behavior of the refractive index, as dictated by the equation that establishes their intrinsic relationship.

### Impedance spectroscopy

Analyzing the impedance over a range of frequencies provides information about the material's response to different electrical signals, especially in the context of energy storage devices. This can reveal the frequency-dependent behavior of the dielectric properties, which is crucial for designing energy storage devices that operate over a wide range of frequencies. The impedance provides insights into the dielectric properties of materials, including their ability to store and release electrical energy.

The real part of impedance represents the resistive elements in the material, such as the losses associated with energy dissipation. Understanding these losses is essential for optimizing energy storage efficiency and minimizing heat generation during charge and discharge cycles. While, the imaginary part of the impedance is associated with the capacitive elements in the material, this indicates the ability of the materials to store electrical energy, which is fundamental for supercapacitor applications.

**Figure 8:**
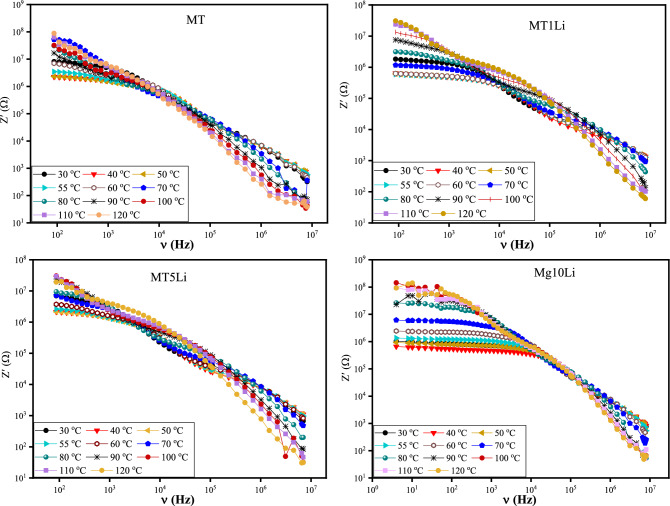
The real impedance (Z') of pure MT and MT doped with Li^+^ versus frequency at temperature range 30–120 °C.

Figure 8 depicts the correlation between real impedance (Z') and frequency (4Hz–8MHz) across a range of temperatures (30–120 °C) for MTXLi samples (with varying x values ranging from 0 to 20%). The behavior of the real impedance is characterized by a distinct pattern: a plateau emerges at low frequencies, followed by a two-step decline as frequency increases.

The plateau observed at low frequencies is a result of the DC resistance to the direct DC current, originating from the presence of free charge carriers. As frequency increases, the impedance exhibits two-steps decrease within the intermediate and high frequency region. This phenomenon can be attributed to the influence of grain boundaries and grains, respectively.

Figure 8 shows that doping with Li^+^ ions decrease the real impedance of the MTXLi. Therefore, doping with Li^+^ ions enhance the total conductivity of MTXLi. This effect is due to increasing the total charge carriers and oxygen vacancies.

The real impedance (Z') maintains a relatively consistent pattern across all temperatures as frequency increases. At low frequency region, the real impedance maintains a plateau region and decreases with increasing temperature up to the transition temperature (Tc), this behavior is due to increasing conductivity with temperature. This trend confirms that samples are thermally activated below the transition temperature (Tc). After transition temperature, the real impedance increases with further increase in temperature due to the anomalous behavior of samples above the transition temperature. The absorbed thermal energy above the transition temperature may be consumed in the structural transformations and the reconstruction of the new phase. Beyond the plateau region, the real impedance sharply declines as frequencies increase^52,53^.

Notably, in the high-frequency range, there is a convergence of the real impedance (Z') across all tested temperatures. This convergence is attributed to the release of space charge, resulting in a reduction of barrier effects^52,54^.

**Figure 9:**
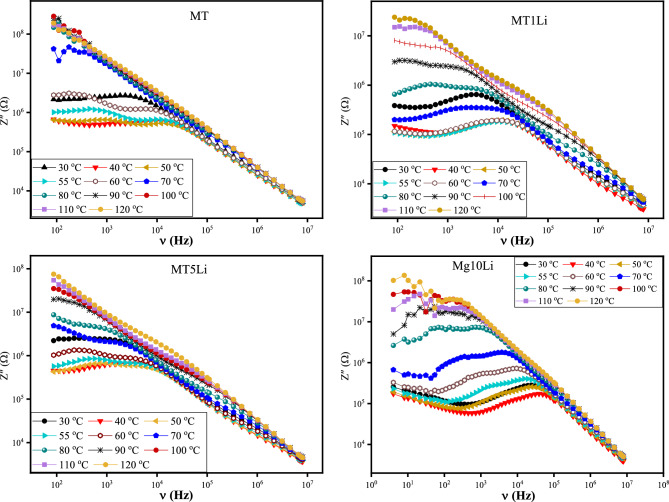
the imaginary impedance (Z") of pure MT and MT doped with Li^+^ versus frequency at temperature range 30–120 °C.

The imaginary impedance curve, Fig. 9, shows the same behavior as the real impedance, it shows a presence of two relaxation peaks that confirms the effect of the grain boundaries and grains. The first peak refers to the mechanism of conductivity due to the long-range hopping of the charge carriers in the grain boundaries. While, the second peak refers to the confined motility of the charge carriers in the grains, short range hopping. The peaks position shifts with increasing temperature confirming the temperature dependent of the relaxation nature of the samples. While, the asymmetric broadening of the peaks confirming the non-Debye relaxation behavior of the samples^55,56^.

**Figure 10:**
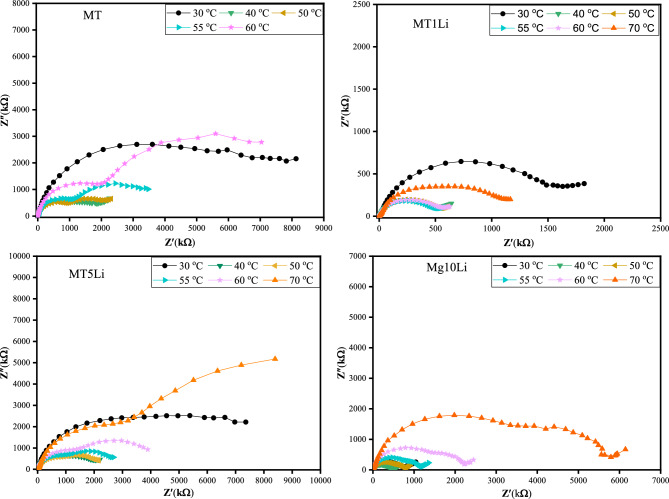
The Cole–Cole plot of pure MT and MT doped with Li^+^ versus frequency at different temperatures.

Figure 10 presents the Cole–Cole plot of pure MT and MT doped with Li^+^. The plots exhibit semicircles that can be deconstructed into two separate semicircles, each one is related to the impedance of distinct microstructure regions. The split of the Cole–Cole plot into two semicircles becomes evident with increasing temperature. The splitting of the semicircles arises from the presence of different relaxation behaviors inside samples. The first semicircle represents the bulk properties of the samples at high frequency, it can be represented by a combination of parallel bulk capacitance C_b_ and bulk resistance R_b_ (R_b_ is the intercept of the first semicircle with the real impedance axis (Z')). While the second semicircle at low frequency arises due to the presence of interfacial capacitance at grain boundaries, it can be represented by a parallel combination between the capacitance C_gb_ and resistance R_gb_ of the grain boundary (R_gb_ is the intercept of the second semicircle with real impedance axis (Z'))^57^. Notably, the centers of these semicircles reside under the axis of the real impedance (Z'), signifying a departure from Debye relaxation behavior. The radius of the circles reduces with increasing temperature until the critical point of the phase transition (Tc) and increases after this transition point. Below the transition point, the behavior of the Cole–Cole plot confirms the negative temperature coefficient of resistance (NTCR) as the resistance and the relaxation time decrease with increasing temperature^55,58^.

**Figure 11:**
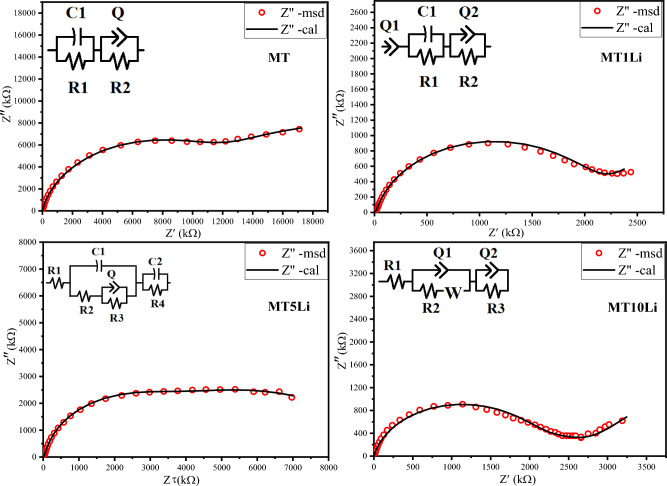
The Nyquist plot at room temperature with the equivalent circuits.

In Fig. 11, the Nyquist plot illustrates complex impedance plots of Z' versus Z'' and the equivalent circuit for each sample at room temperature. The experimental data were fitted according to the equivalent circuit, and the obtained results are listed in Table 4.

**Table 4:** The values of the equivalent circuit elements obtained from the fitting process.

**MT** (CR)(QR)	**C1 (F)**	**R1 (Ω)**	**Qgb (F)**	**N**	**R2 (Ω)**			
	1.95E-10	1.328E7	3.09 E-11	0.8856	1.34E7			
**MT1Li** Q(CR)(QR)	**Q1**	**n**	**C1 (F)**	**R1(Ω)**	**Q2 (F)**	**n**	**R2 (Ω)**	
	4.796E-8	0.5659	1.185E-11	1.12E3	9.77E-11	0.8915	2.016 E6	
**MT5Li** R(C(R(QR)))(CR)	**R1(Ω)**	**C1 (F)**	**R2 (Ω)**	**Q (F)**	**n**	**R3(Ω)**	**C (F)**	**R4 (Ω)**
	1.559E2	5.78E-12	2.89E4	1.17E-10	0.705	7.04E6	3.71E-11	2.684E6
**MT10Li** R(Q(RW))(QR)	**R1 (Ω)**	**Q1 (F)**	**n**	**R2 (Ω)**	**W**	**Q2 (F)**	**N**	**R3 (Ω)**
	1E-2	4.96E-11	0.87	2.087E6	2.151	1.316	0.67	5.25E5

The equivalent circuit revealed the presence of a constant phase element (Q) rather than an ideal capacitor (C) in the Nyquist plot. This observation suggests the existence of non-Debye-type dielectric relaxation^59^.

### AC-conductivity

**Figure 12:**
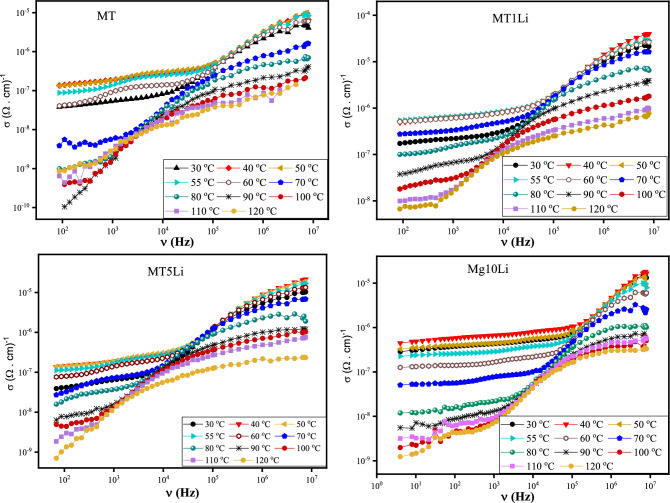
The experimental conductivity (σ) of pure MT and MT doped with Li^+^ versus frequency at temperature range 30 − 120 °C.

MgTiO_3_ is a perovskite-type oxide material with interesting electrical properties. The AC conductivity of MgTiO_3_ depends on factors such as temperature, frequency, and microstructure. Typically, the AC conductivity of MgTiO_3_ is studied in the range of 4 Hz to 8 MHz of frequency and in the temperature range from 25 to 120 °C, Fig. 12.

The conductivity of MTxLi exhibits distinct behaviors in the measured frequency range and it can be divided into two separate regions. At lower frequencies, the conductivity measurements demonstrate a consistent value despite the increase in frequency. This consistent behavior forms a stable plateau in the data. This plateau corresponds to the contribution of direct current (σ_dc_) conductivity to the overall conductivity. This phenomenon is likely attributed to the movement of charge carriers across long distances in an organized manner^8^.

Conversely, at higher frequencies, the measured conductivity displays a clear relationship with frequency, indicating an increase in conductivity as the frequency rises. This type of frequency-dependent conductivity is termed ac conductivity (σ_ac_). It arises from the localized motility (short-range motion) of charge carriers in grains. Furthermore, Li^+^ increases the total conductivity compared to the undoped sample. This comportment can be ascribed to the increasing charge carriers, such as oxygen vacancies released to maintain the charge neutrality of the perovskite MgTiO_3_.

The observed plateau in the measured conductivity becomes more prominent and expands across a wider frequency range as the temperature rises. This trend is depicted in Fig. 12. As temperature increases, the overall conductivity magnitude also rises up to a certain threshold, Curie temperature (Tc). Beyond the Curie temperature (Tc), however, further temperature increases lead to a decrease in conductivity. The Curie temperature (Tc) marks a pivotal juncture at which the material's properties undergo significant transformations^51^.

The AC conductivity in Fig. 12 exhibits irregular variation due to the presence of a ferroelectric transition occurring around 50 °C in the prepared samples. This transition significantly influences the dielectric and electrical properties, causing their behavior to deviate from a regular pattern around this critical temperature. The unique characteristics associated with the ferroelectric transition introduce complexities in the conductivity data, resulting in non-uniform and anomalous variations that cannot be explained by a conventional or regular pattern. Therefore, the irregularities in the AC conductivity data can be attributed to the distinct effects of the ferroelectric transition on the material's properties.

### Electrochemical properties

In electrochemical systems (e.g. energy storage devices, supercapacitors, and /or sensors), chemical and physical processes could be characterized and studied effectively using the electrochemical impedance spectroscopy (EIS) and cyclic voltammetry (CV) techniques as non-destructive investigating tools. Thus, such electrochemical methods could be used to monitor the performance and stability of any promised materials and their charge transport properties.

In the EIS technique, the Nyquist plots presented the imagine and real part of impedance, at high frequency, the semicircle part expresses the electron transfer process, whereas the semicircle diameter is the charge transfer resistance (R_ct_) value of the probe at the electrode interface. CV and EIS techniques are used for electrochemical characterizations of newly synthesized materials by using a solution of ferro / ferricyanide /KCl as a stranded redox mediator.

**Figure 13:**
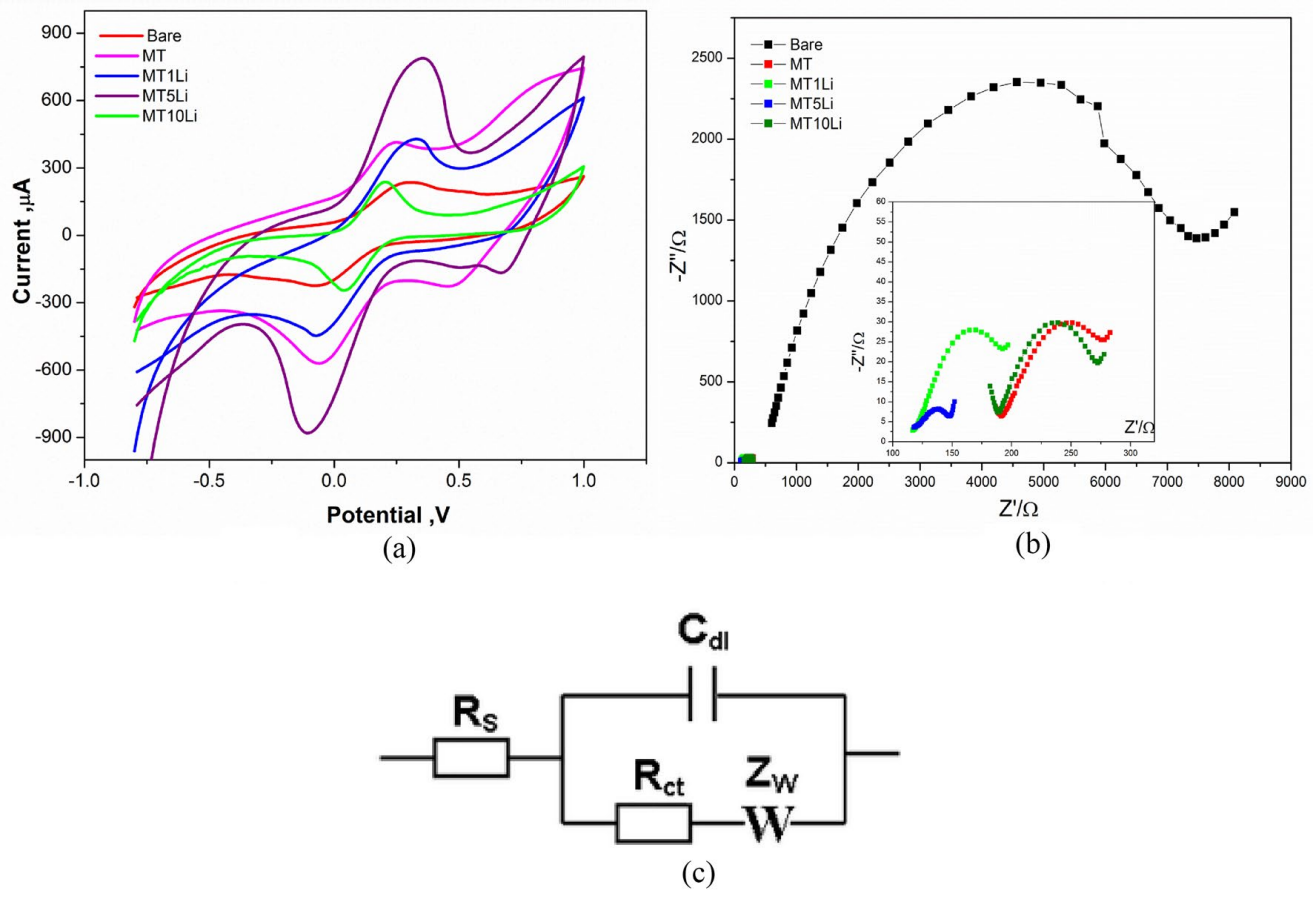
(**A**) The Cyclic voltammetry and (**B**) EIS measurements of (Bare, MT, MT1Li, MT5Li, and MT10Li) were conducted in a ferri/ferrocyanide (5 mM) and KCl (0.1M) mixture solution. (**C**) Fitting circuit.

Cyclic voltammetric studies are illustrated in Fig. 13A. The highest faradic current and fast reversible faradaic redox probe were approved for all modified electrodes (MT, MT1Li, MT5Li and MT10Li). The faradic current of the modified electrodes increased from MT, MT1Li up to MT5Li giving the highest faradic current then decreased for MT10Li.

The oxidation/reduction peaks appear for MT at 0.236 V and −0.064 V, for MT1Li at 0.315 and −0.08 V and for MT10Li at 0.202 V and 0.037 V. The higher value of oxidation/reduction peaks produced for MT5Li at 0.35 and −0.109 V, therefore the best one with high conductivity was MT5Li.

From ElS studies, (the Nyquist plots in Fig. 13B) and the circuit used for fitting (Fig. 13C) semicircle at high frequency indicates the electron transfer process, whereas the semicircle represents to resistance charge transfer (R_ct_) of the modified SPE interface. However, the charge transfer resistance decreased (see Table 5), MT (Rct = 55.9 Ω), MT1Li (Rct = 61.3 Ω) and MT10Li (Rct = 48.4 Ω). In MT5Li the semicircle diameter becomes smaller (Rct = 18.7Ω) compared to Bare/unmodified electrode (Rct = 425.2Ω), indicating a higher efficiency for interfacial electron transfer. These results demonstrate that MT5Li represents an interesting electrode for electrochemical applications.

**Table 5:** The CV& EIS electrochemical data of the prepared materials modified SPEs.

Electrode composite	*I*_*a*_ (µA)		*I*_*c*_ (µA)		E _*oxd*_ (V)			E_*red*_ (V)	E_1/2_ (V)		R_s_ (Ω)		R_ct_ (Ω)		C µF		W (Ω)	
Bare (unmodified)	229		−222.2		0.291			−0.06	0.115		704.9		4253.2		3.8		18,656	
MT	418		−577		0.236			−0.064	0.086		194.5		55.9		11.7		174.5	
MT1Li	427.3		−439		0.315			−0.08	0.117		188.7		61.3		12.5		146.5	
MT5Li	791		−880		0.35			−0.109	0.295		121.3		18.7		125.1		68.02	
MT10Li	234.2		−240.4		0.202			0.037	0.119		122.4		48.4		28.4		145.5	

From CV redox peaks of the prepared materials, fast oxidation reduction of faradaic redox was obtained due to the faradaic charge transfer and intercalation of protons at the modified SPE surface which present a significant feature of materials as a supercapacitor.

**Figure 14:**
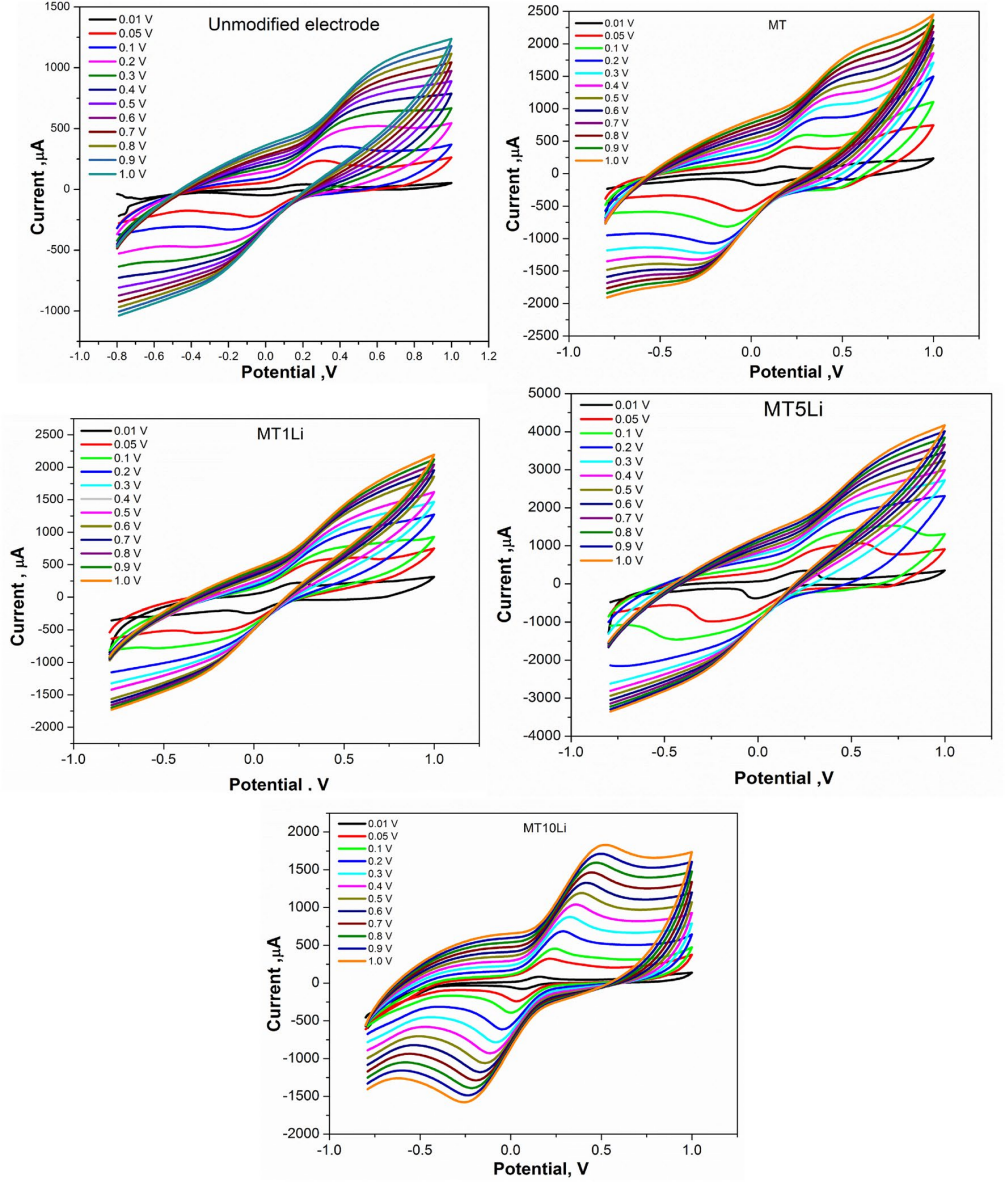
CV of prepared MT, MT1Li, MT5Li and MT10Li modified SPE at different scan rates.

**Figure 15:**
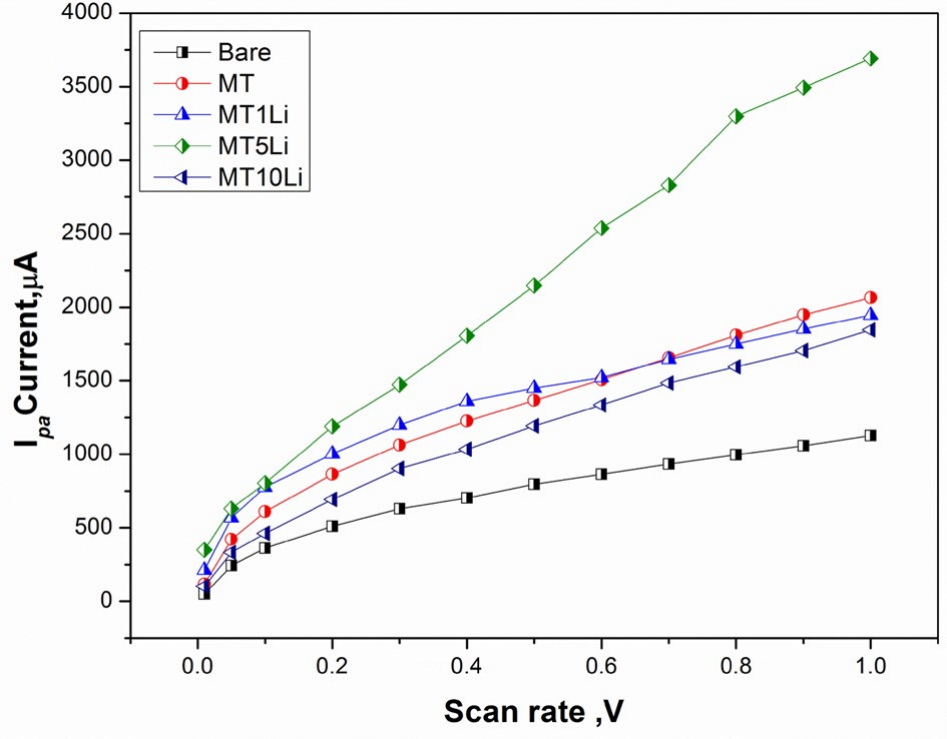
the oxidation current study of unmodified, MT, MT1Li, MT5Li and MT10Li modified SPE at different scan rate.

The scan rate effect on the electrochemical behavior of each prepared material (MT, MT1Li, MT5Li, and MT10Li) was studied by CV (Fig. 14). At different scan rates from 0.1 up to 1 V/s, the ip_a_ and ip_c_ redox peak currents increased with increasing the scan rate for all prepared materials. In Fig. 15, unmodified SPE produced lower peak current values. However, in MT, MT1Li and MT10Li the current values of peaks increased. For the MT5Li, the highest values of peak current were obtained due to its higher capacitive properties. Furthermore, the cyclic voltammetry study is a significant supercapacitor property. However, all of the synthesized materials were stable electrochemically at various scan rates without any damage to the modified SPE surface which is important for energy storage applications.

## Conclusions

MgTiO_3_ is successively prepared by sol–gel chemistry. The Rietveld refinement confirms the formation of perovskite MgTiO_3_ in the trigonal phase beside traces of orthorhombic MgTi_2_O_5_. The SEM results indicated that the fabricated perovskite nanoceramics exhibit a mesoporous morphology and distinct structures. The energy of the band gap experiences a sudden increase with the addition of Li^+^, followed by a slight decrease which is attributed to short-range ordering (SRO) mechanisms and disorder. The frequency-dependent behaviors of the dielectric properties, analyzed across a broad temperature range, offer crucial information for designing energy storage devices with optimal efficiency. The real and imaginary parts of impedance reveal distinct patterns, showcasing the influence of grain boundaries, grains, and the impact of Li^+^ doping on the overall conductivity of MTxLi. The AC conductivity studies emphasize the significance of temperature, frequency, and microstructure in influencing the conductivity behavior. The electrochemical impedance spectroscopy (EIS) and cyclic voltammetry (CV) techniques have proven to be valuable tools for the comprehensive characterization of newly synthesized materials intended for use in electrochemical systems such as energy storage devices, supercapacitors, and sensors. Nyquist plots obtained from EIS provided insights into the electron transfer processes, with the semicircle diameter representing the charge transfer resistance (Rct) at the electrode interface. Concurrently, CV studies revealed that all modified electrodes (MT, MT1Li, MT5Li, and MT10Li) exhibited a notable faradic current, with MT5Li demonstrating the highest conductivity. These results highlight that the MT5Li configuration (at potentials of 0.35 and −0.109 V) displays enhanced interfacial electron transfer efficiency (Rct = 425.2 Ω) compared with the 0.0Li-MT sample. This makes MT5Li an intriguing electrode option for various electrochemical applications (energy Storage and Supercapacitors). Moreover, the fast oxidation–reduction peaks observed in CV pointed towards efficient faradaic charge transfer and proton intercalation at the modified solid-state electrode surface, highlighting the potential application of MT5Li as a superior material for supercapacitors. Overall, the combined use of EIS and CV techniques has provided a thorough understanding of the electrochemical properties of the synthesized materials, emphasizing the significance of MT5Li for future electrochemical applications.

### Supplementary Information

Supplementary Information 1.

Supplementary Information 2.

Supplementary Information 3.

Supplementary Information 4.

Supplementary Information 5.

Supplementary Information 6.

Supplementary Information 7.

Supplementary Information 8.

## Data Availability

Data available upon request from the corresponding author on reasonable request.
