# Supplementary material for: Advancing energy storage and supercapacitor applications through the development of Li+-doped MgTiO3 perovskite nano-ceramics
Source: Sci Rep. 2024 Jan 22;14:1849. doi: 10.1038/s41598-024-52262-6 (PMC10803294; doi:10.1038/s41598-024-52262-6)
Supplement: Supplementary file 1 — Supplementary Information 1. [file 41598_2024_52262_MOESM1_ESM.docx]

Diffuse reflectance (Sample: MT)

| TITLE | MT-DR |
| --- | --- |
| DATA TYPE | |
| ORIGIN | JASCO |
| OWNER |  |
| DATE | 23/05/22 |
| TIME | 10:20:41 |
| SPECTROMETER/DATA SYSTEM | JASCO Corp., V-570, Rev. 1.00 |
| RESOLUTION | |
| DELTAX | -2 |
| XUNITS | NANOMETERS |
| YUNITS | REFLECTANCE |
| FIRSTX | 2500 |
| LASTX | 190 |
| NPOINTS | 1156 |
| FIRSTY | 0.7871 |
| MAXY | 0.9379 |
| MINY | 0.30105 |
| XYDATA |  |
| 2500 | 78.71 |
| 2498 | 79.21 |
| 2496 | 80.244 |
| 2494 | 79.03 |
| 2492 | 79.562 |
| 2490 | 80.816 |
| 2488 | 80.372 |
| 2486 | 77.759 |
| 2484 | 77.756 |
| 2482 | 75.908 |
| 2480 | 76.207 |
| 2478 | 79.276 |
| 2476 | 80.095 |
| 2474 | 80.798 |
| 2472 | 80.922 |
| 2470 | 80.133 |
| 2468 | 77.973 |
| 2466 | 77.857 |
| 2464 | 77.48 |
| 2462 | 76.381 |
| 2460 | 76.737 |
| 2458 | 76.905 |
| 2456 | 78.307 |
| 2454 | 78.258 |
| 2452 | 77.744 |
| 2450 | 77.656 |
| 2448 | 76.773 |
| 2446 | 77.196 |
| 2444 | 77.958 |
| 2442 | 77.841 |
| 2440 | 78.754 |
| 2438 | 78.626 |
| 2436 | 80.111 |
| 2434 | 80.147 |
| 2432 | 78.971 |
| 2430 | 80.127 |
| 2428 | 79.472 |
| 2426 | 79.311 |
| 2424 | 79.458 |
| 2422 | 78.501 |
| 2420 | 78.158 |
| 2418 | 77.641 |
| 2416 | 77.572 |
| 2414 | 78.132 |
| 2412 | 78.99 |
| 2410 | 78.848 |
| 2408 | 79.354 |
| 2406 | 78.471 |
| 2404 | 78.04 |
| 2402 | 79.48 |
| 2400 | 79.86 |
| 2398 | 80.335 |
| 2396 | 79.957 |
| 2394 | 79.038 |
| 2392 | 78.58 |
| 2390 | 78.769 |
| 2388 | 78.612 |
| 2386 | 79.839 |
| 2384 | 79.998 |
| 2382 | 80.31 |
| 2380 | 81.255 |
| 2378 | 79.935 |
| 2376 | 79.585 |
| 2374 | 78.664 |
| 2372 | 78.549 |
| 2370 | 78.779 |
| 2368 | 78.598 |
| 2366 | 79.211 |
| 2364 | 78.759 |
| 2362 | 78.553 |
| 2360 | 78.504 |
| 2358 | 78.244 |
| 2356 | 78.863 |
| 2354 | 79.803 |
| 2352 | 80.409 |
| 2350 | 80.993 |
| 2348 | 80.929 |
| 2346 | 80.295 |
| 2344 | 80.159 |
| 2342 | 80.368 |
| 2340 | 79.775 |
| 2338 | 80.609 |
| 2336 | 79.911 |
| 2334 | 78.927 |
| 2332 | 78.854 |
| 2330 | 78.607 |
| 2328 | 78.841 |
| 2326 | 79.286 |
| 2324 | 79.688 |
| 2322 | 78.693 |
| 2320 | 78.894 |
| 2318 | 78.417 |
| 2316 | 77.963 |
| 2314 | 78.351 |
| 2312 | 78.427 |
| 2310 | 78.712 |
| 2308 | 78.738 |
| 2306 | 79.33 |
| 2304 | 79.51 |
| 2302 | 79.284 |
| 2300 | 79.326 |
| 2298 | 78.804 |
| 2296 | 78.448 |
| 2294 | 78.557 |
| 2292 | 77.795 |
| 2290 | 77.366 |
| 2288 | 77.444 |
| 2286 | 77.311 |
| 2284 | 77.882 |
| 2282 | 77.458 |
| 2280 | 77.756 |
| 2278 | 77.687 |
| 2276 | 77.888 |
| 2274 | 78.599 |
| 2272 | 78.496 |
| 2270 | 78.587 |
| 2268 | 78.416 |
| 2266 | 78.071 |
| 2264 | 77.82 |
| 2262 | 78.192 |
| 2260 | 78.827 |
| 2258 | 78.778 |
| 2256 | 78.442 |
| 2254 | 78.175 |
| 2252 | 77.46 |
| 2250 | 78.273 |
| 2248 | 78.267 |
| 2246 | 78.537 |
| 2244 | 78.924 |
| 2242 | 78.369 |
| 2240 | 78.161 |
| 2238 | 78.115 |
| 2236 | 77.799 |
| 2234 | 77.857 |
| 2232 | 78.037 |
| 2230 | 77.388 |
| 2228 | 77.395 |
| 2226 | 77.586 |
| 2224 | 77.566 |
| 2222 | 77.846 |
| 2220 | 77.795 |
| 2218 | 77.98 |
| 2216 | 77.775 |
| 2214 | 77.859 |
| 2212 | 77.706 |
| 2210 | 77.415 |
| 2208 | 78.349 |
| 2206 | 78.196 |
| 2204 | 78.453 |
| 2202 | 78.839 |
| 2200 | 78.474 |
| 2198 | 78.523 |
| 2196 | 78.593 |
| 2194 | 78.141 |
| 2192 | 77.59 |
| 2190 | 77.64 |
| 2188 | 77.557 |
| 2186 | 78.008 |
| 2184 | 78.732 |
| 2182 | 78.971 |
| 2180 | 79.196 |
| 2178 | 78.762 |
| 2176 | 78.825 |
| 2174 | 79.268 |
| 2172 | 79.376 |
| 2170 | 79.47 |
| 2168 | 79.74 |
| 2166 | 79.403 |
| 2164 | 79.233 |
| 2162 | 79.366 |
| 2160 | 79.304 |
| 2158 | 79.903 |
| 2156 | 80.416 |
| 2154 | 80.676 |
| 2152 | 80.735 |
| 2150 | 80.83 |
| 2148 | 80.453 |
| 2146 | 80.436 |
| 2144 | 80.473 |
| 2142 | 80.565 |
| 2140 | 80.234 |
| 2138 | 80.333 |
| 2136 | 80.787 |
| 2134 | 80.536 |
| 2132 | 81.078 |
| 2130 | 81.428 |
| 2128 | 81.387 |
| 2126 | 81.221 |
| 2124 | 81.03 |
| 2122 | 80.728 |
| 2120 | 80.073 |
| 2118 | 80.028 |
| 2116 | 80.347 |
| 2114 | 79.937 |
| 2112 | 80.398 |
| 2110 | 80.888 |
| 2108 | 80.42 |
| 2106 | 80.617 |
| 2104 | 80.462 |
| 2102 | 80.357 |
| 2100 | 80.232 |
| 2098 | 80.384 |
| 2096 | 80.444 |
| 2094 | 80.211 |
| 2092 | 80.628 |
| 2090 | 80.677 |
| 2088 | 80.54 |
| 2086 | 80.052 |
| 2084 | 80.084 |
| 2082 | 79.737 |
| 2080 | 79.71 |
| 2078 | 79.842 |
| 2076 | 79.658 |
| 2074 | 79.816 |
| 2072 | 79.688 |
| 2070 | 79.446 |
| 2068 | 79.309 |
| 2066 | 79.379 |
| 2064 | 79.482 |
| 2062 | 79.605 |
| 2060 | 79.552 |
| 2058 | 79.542 |
| 2056 | 79.276 |
| 2054 | 79.394 |
| 2052 | 79.327 |
| 2050 | 78.966 |
| 2048 | 78.838 |
| 2046 | 78.928 |
| 2044 | 78.742 |
| 2042 | 78.552 |
| 2040 | 78.831 |
| 2038 | 78.3 |
| 2036 | 78.522 |
| 2034 | 78.865 |
| 2032 | 78.515 |
| 2030 | 78.429 |
| 2028 | 77.935 |
| 2026 | 77.93 |
| 2024 | 78.005 |
| 2022 | 78.068 |
| 2020 | 78.526 |
| 2018 | 78.21 |
| 2016 | 78.088 |
| 2014 | 78.21 |
| 2012 | 77.741 |
| 2010 | 77.455 |
| 2008 | 77.219 |
| 2006 | 76.898 |
| 2004 | 76.959 |
| 2002 | 77.135 |
| 2000 | 77.356 |
| 1998 | 77.473 |
| 1996 | 77.5 |
| 1994 | 77.037 |
| 1992 | 77.079 |
| 1990 | 76.791 |
| 1988 | 76.883 |
| 1986 | 77.15 |
| 1984 | 76.907 |
| 1982 | 76.905 |
| 1980 | 76.607 |
| 1978 | 76.373 |
| 1976 | 76.125 |
| 1974 | 76.268 |
| 1972 | 76.407 |
| 1970 | 76.713 |
| 1968 | 76.666 |
| 1966 | 76.619 |
| 1964 | 76.504 |
| 1962 | 76.114 |
| 1960 | 76.141 |
| 1958 | 76.162 |
| 1956 | 76.057 |
| 1954 | 76.271 |
| 1952 | 76.241 |
| 1950 | 76.017 |
| 1948 | 75.955 |
| 1946 | 75.603 |
| 1944 | 75.681 |
| 1942 | 75.837 |
| 1940 | 75.82 |
| 1938 | 76.123 |
| 1936 | 76.248 |
| 1934 | 76.539 |
| 1932 | 76.703 |
| 1930 | 76.688 |
| 1928 | 76.619 |
| 1926 | 76.402 |
| 1924 | 76.39 |
| 1922 | 76.552 |
| 1920 | 76.344 |
| 1918 | 76.353 |
| 1916 | 75.887 |
| 1914 | 75.957 |
| 1912 | 76.211 |
| 1910 | 75.961 |
| 1908 | 76.692 |
| 1906 | 76.48 |
| 1904 | 77.098 |
| 1902 | 77.2 |
| 1900 | 77.736 |
| 1898 | 77.65 |
| 1896 | 77.637 |
| 1894 | 77.479 |
| 1892 | 77.601 |
| 1890 | 77.846 |
| 1888 | 77.663 |
| 1886 | 77.486 |
| 1884 | 77.517 |
| 1882 | 77.372 |
| 1880 | 77.693 |
| 1878 | 77.966 |
| 1876 | 77.992 |
| 1874 | 78.186 |
| 1872 | 78.34 |
| 1870 | 78.456 |
| 1868 | 78.448 |
| 1866 | 78.611 |
| 1864 | 78.529 |
| 1862 | 78.488 |
| 1860 | 78.6 |
| 1858 | 78.657 |
| 1856 | 78.812 |
| 1854 | 78.895 |
| 1852 | 78.947 |
| 1850 | 79.003 |
| 1848 | 79.025 |
| 1846 | 79.066 |
| 1844 | 79.148 |
| 1842 | 79.147 |
| 1840 | 79.06 |
| 1838 | 79.298 |
| 1836 | 79.227 |
| 1834 | 79.258 |
| 1832 | 79.485 |
| 1830 | 79.254 |
| 1828 | 79.332 |
| 1826 | 79.387 |
| 1824 | 79.244 |
| 1822 | 79.209 |
| 1820 | 79.201 |
| 1818 | 79.201 |
| 1816 | 79.238 |
| 1814 | 79.381 |
| 1812 | 79.348 |
| 1810 | 79.357 |
| 1808 | 79.463 |
| 1806 | 79.402 |
| 1804 | 79.48 |
| 1802 | 79.514 |
| 1800 | 79.446 |
| 1798 | 79.364 |
| 1796 | 79.319 |
| 1794 | 79.317 |
| 1792 | 79.325 |
| 1790 | 79.46 |
| 1788 | 79.483 |
| 1786 | 79.56 |
| 1784 | 79.619 |
| 1782 | 79.668 |
| 1780 | 79.681 |
| 1778 | 79.527 |
| 1776 | 79.411 |
| 1774 | 79.384 |
| 1772 | 79.351 |
| 1770 | 79.357 |
| 1768 | 79.447 |
| 1766 | 79.383 |
| 1764 | 79.479 |
| 1762 | 79.552 |
| 1760 | 79.558 |
| 1758 | 79.61 |
| 1756 | 79.625 |
| 1754 | 79.691 |
| 1752 | 79.779 |
| 1750 | 79.772 |
| 1748 | 79.757 |
| 1746 | 79.702 |
| 1744 | 79.687 |
| 1742 | 79.722 |
| 1740 | 79.877 |
| 1738 | 79.944 |
| 1736 | 80.018 |
| 1734 | 80.122 |
| 1732 | 79.966 |
| 1730 | 80.054 |
| 1728 | 80.031 |
| 1726 | 80.035 |
| 1724 | 80.157 |
| 1722 | 80.115 |
| 1720 | 80.157 |
| 1718 | 80.194 |
| 1716 | 80.235 |
| 1714 | 80.259 |
| 1712 | 80.3 |
| 1710 | 80.315 |
| 1708 | 80.211 |
| 1706 | 80.221 |
| 1704 | 80.208 |
| 1702 | 80.253 |
| 1700 | 80.421 |
| 1698 | 80.461 |
| 1696 | 80.542 |
| 1694 | 80.58 |
| 1692 | 80.631 |
| 1690 | 80.564 |
| 1688 | 80.566 |
| 1686 | 80.441 |
| 1684 | 80.33 |
| 1682 | 80.404 |
| 1680 | 80.327 |
| 1678 | 80.514 |
| 1676 | 80.535 |
| 1674 | 80.551 |
| 1672 | 80.662 |
| 1670 | 80.467 |
| 1668 | 80.531 |
| 1666 | 80.481 |
| 1664 | 80.304 |
| 1662 | 80.509 |
| 1660 | 80.476 |
| 1658 | 80.493 |
| 1656 | 80.484 |
| 1654 | 80.394 |
| 1652 | 80.289 |
| 1650 | 80.376 |
| 1648 | 80.493 |
| 1646 | 80.443 |
| 1644 | 80.552 |
| 1642 | 80.433 |
| 1640 | 80.348 |
| 1638 | 80.302 |
| 1636 | 80.222 |
| 1634 | 80.392 |
| 1632 | 80.441 |
| 1630 | 80.605 |
| 1628 | 80.707 |
| 1626 | 80.543 |
| 1624 | 80.558 |
| 1622 | 80.538 |
| 1620 | 80.541 |
| 1618 | 80.65 |
| 1616 | 80.716 |
| 1614 | 80.686 |
| 1612 | 80.595 |
| 1610 | 80.528 |
| 1608 | 80.479 |
| 1606 | 80.518 |
| 1604 | 80.581 |
| 1602 | 80.684 |
| 1600 | 80.704 |
| 1598 | 80.681 |
| 1596 | 80.727 |
| 1594 | 80.707 |
| 1592 | 80.777 |
| 1590 | 80.713 |
| 1588 | 80.722 |
| 1586 | 80.736 |
| 1584 | 80.702 |
| 1582 | 80.746 |
| 1580 | 80.644 |
| 1578 | 80.611 |
| 1576 | 80.57 |
| 1574 | 80.558 |
| 1572 | 80.642 |
| 1570 | 80.657 |
| 1568 | 80.669 |
| 1566 | 80.736 |
| 1564 | 80.715 |
| 1562 | 80.715 |
| 1560 | 80.786 |
| 1558 | 80.811 |
| 1556 | 80.858 |
| 1554 | 80.943 |
| 1552 | 80.928 |
| 1550 | 80.81 |
| 1548 | 80.78 |
| 1546 | 80.711 |
| 1544 | 80.748 |
| 1542 | 80.77 |
| 1540 | 80.778 |
| 1538 | 80.802 |
| 1536 | 80.778 |
| 1534 | 80.809 |
| 1532 | 80.795 |
| 1530 | 80.725 |
| 1528 | 80.746 |
| 1526 | 80.762 |
| 1524 | 80.784 |
| 1522 | 80.852 |
| 1520 | 80.777 |
| 1518 | 80.864 |
| 1516 | 80.833 |
| 1514 | 80.862 |
| 1512 | 80.958 |
| 1510 | 80.895 |
| 1508 | 80.852 |
| 1506 | 80.809 |
| 1504 | 80.742 |
| 1502 | 80.708 |
| 1500 | 80.816 |
| 1498 | 80.749 |
| 1496 | 80.753 |
| 1494 | 80.783 |
| 1492 | 80.748 |
| 1490 | 80.835 |
| 1488 | 80.737 |
| 1486 | 80.747 |
| 1484 | 80.671 |
| 1482 | 80.731 |
| 1480 | 80.831 |
| 1478 | 80.793 |
| 1476 | 80.806 |
| 1474 | 80.673 |
| 1472 | 80.628 |
| 1470 | 80.63 |
| 1468 | 80.623 |
| 1466 | 80.669 |
| 1464 | 80.712 |
| 1462 | 80.759 |
| 1460 | 80.796 |
| 1458 | 80.774 |
| 1456 | 80.687 |
| 1454 | 80.67 |
| 1452 | 80.69 |
| 1450 | 80.61 |
| 1448 | 80.617 |
| 1446 | 80.582 |
| 1444 | 80.541 |
| 1442 | 80.608 |
| 1440 | 80.678 |
| 1438 | 80.672 |
| 1436 | 80.753 |
| 1434 | 80.789 |
| 1432 | 80.82 |
| 1430 | 80.856 |
| 1428 | 80.829 |
| 1426 | 80.822 |
| 1424 | 80.826 |
| 1422 | 80.817 |
| 1420 | 80.77 |
| 1418 | 80.807 |
| 1416 | 80.824 |
| 1414 | 80.824 |
| 1412 | 80.882 |
| 1410 | 80.913 |
| 1408 | 80.921 |
| 1406 | 80.948 |
| 1404 | 80.943 |
| 1402 | 80.916 |
| 1400 | 80.93 |
| 1398 | 80.953 |
| 1396 | 81.041 |
| 1394 | 81.115 |
| 1392 | 81.124 |
| 1390 | 81.17 |
| 1388 | 81.197 |
| 1386 | 81.182 |
| 1384 | 81.214 |
| 1382 | 81.273 |
| 1380 | 81.279 |
| 1378 | 81.305 |
| 1376 | 81.325 |
| 1374 | 81.31 |
| 1372 | 81.37 |
| 1370 | 81.437 |
| 1368 | 81.464 |
| 1366 | 81.496 |
| 1364 | 81.49 |
| 1362 | 81.488 |
| 1360 | 81.585 |
| 1358 | 81.672 |
| 1356 | 81.752 |
| 1354 | 81.772 |
| 1352 | 81.751 |
| 1350 | 81.731 |
| 1348 | 81.695 |
| 1346 | 81.716 |
| 1344 | 81.747 |
| 1342 | 81.713 |
| 1340 | 81.68 |
| 1338 | 81.667 |
| 1336 | 81.63 |
| 1334 | 81.724 |
| 1332 | 81.75 |
| 1330 | 81.756 |
| 1328 | 81.768 |
| 1326 | 81.757 |
| 1324 | 81.811 |
| 1322 | 81.86 |
| 1320 | 81.814 |
| 1318 | 81.765 |
| 1316 | 81.748 |
| 1314 | 81.708 |
| 1312 | 81.784 |
| 1310 | 81.796 |
| 1308 | 81.804 |
| 1306 | 81.815 |
| 1304 | 81.822 |
| 1302 | 81.862 |
| 1300 | 81.853 |
| 1298 | 81.867 |
| 1296 | 81.893 |
| 1294 | 81.871 |
| 1292 | 81.891 |
| 1290 | 81.871 |
| 1288 | 81.863 |
| 1286 | 81.883 |
| 1284 | 81.904 |
| 1282 | 81.959 |
| 1280 | 81.897 |
| 1278 | 81.921 |
| 1276 | 81.872 |
| 1274 | 81.878 |
| 1272 | 81.907 |
| 1270 | 81.92 |
| 1268 | 81.962 |
| 1266 | 81.948 |
| 1264 | 81.999 |
| 1262 | 82.011 |
| 1260 | 82.02 |
| 1258 | 82.038 |
| 1256 | 82.034 |
| 1254 | 82.065 |
| 1252 | 82.083 |
| 1250 | 82.072 |
| 1248 | 82.079 |
| 1246 | 82.044 |
| 1244 | 82.021 |
| 1242 | 82.059 |
| 1240 | 82.034 |
| 1238 | 82.042 |
| 1236 | 82.099 |
| 1234 | 82.12 |
| 1232 | 82.164 |
| 1230 | 82.189 |
| 1228 | 82.185 |
| 1226 | 82.175 |
| 1224 | 82.138 |
| 1222 | 82.183 |
| 1220 | 82.206 |
| 1218 | 82.205 |
| 1216 | 82.265 |
| 1214 | 82.189 |
| 1212 | 82.143 |
| 1210 | 82.14 |
| 1208 | 82.113 |
| 1206 | 82.075 |
| 1204 | 82.103 |
| 1202 | 82.177 |
| 1200 | 82.186 |
| 1198 | 82.21 |
| 1196 | 82.185 |
| 1194 | 82.219 |
| 1192 | 82.175 |
| 1190 | 82.159 |
| 1188 | 82.194 |
| 1186 | 82.259 |
| 1184 | 82.322 |
| 1182 | 82.364 |
| 1180 | 82.354 |
| 1178 | 82.316 |
| 1176 | 82.37 |
| 1174 | 82.35 |
| 1172 | 82.375 |
| 1170 | 82.335 |
| 1168 | 82.272 |
| 1166 | 82.264 |
| 1164 | 82.204 |
| 1162 | 82.181 |
| 1160 | 82.18 |
| 1158 | 82.224 |
| 1156 | 82.246 |
| 1154 | 82.302 |
| 1152 | 82.304 |
| 1150 | 82.318 |
| 1148 | 82.32 |
| 1146 | 82.358 |
| 1144 | 82.371 |
| 1142 | 82.317 |
| 1140 | 82.276 |
| 1138 | 82.302 |
| 1136 | 82.351 |
| 1134 | 82.398 |
| 1132 | 82.42 |
| 1130 | 82.323 |
| 1128 | 82.276 |
| 1126 | 82.258 |
| 1124 | 82.313 |
| 1122 | 82.401 |
| 1120 | 82.422 |
| 1118 | 82.411 |
| 1116 | 82.379 |
| 1114 | 82.331 |
| 1112 | 82.303 |
| 1110 | 82.32 |
| 1108 | 82.34 |
| 1106 | 82.319 |
| 1104 | 82.307 |
| 1102 | 82.314 |
| 1100 | 82.298 |
| 1098 | 82.323 |
| 1096 | 82.411 |
| 1094 | 82.403 |
| 1092 | 82.432 |
| 1090 | 82.454 |
| 1088 | 82.43 |
| 1086 | 82.46 |
| 1084 | 82.41 |
| 1082 | 82.401 |
| 1080 | 82.376 |
| 1078 | 82.349 |
| 1076 | 82.361 |
| 1074 | 82.35 |
| 1072 | 82.37 |
| 1070 | 82.389 |
| 1068 | 82.394 |
| 1066 | 82.436 |
| 1064 | 82.421 |
| 1062 | 82.413 |
| 1060 | 82.435 |
| 1058 | 82.44 |
| 1056 | 82.468 |
| 1054 | 82.513 |
| 1052 | 82.526 |
| 1050 | 82.541 |
| 1048 | 82.536 |
| 1046 | 82.538 |
| 1044 | 82.539 |
| 1042 | 82.524 |
| 1040 | 82.525 |
| 1038 | 82.472 |
| 1036 | 82.512 |
| 1034 | 82.572 |
| 1032 | 82.618 |
| 1030 | 82.704 |
| 1028 | 82.701 |
| 1026 | 82.681 |
| 1024 | 82.63 |
| 1022 | 82.62 |
| 1020 | 82.657 |
| 1018 | 82.713 |
| 1016 | 82.833 |
| 1014 | 82.871 |
| 1012 | 82.886 |
| 1010 | 82.871 |
| 1008 | 82.827 |
| 1006 | 82.844 |
| 1004 | 82.868 |
| 1002 | 82.902 |
| 1000 | 82.964 |
| 998 | 82.978 |
| 996 | 82.977 |
| 994 | 82.979 |
| 992 | 82.925 |
| 990 | 82.917 |
| 988 | 82.935 |
| 986 | 82.971 |
| 984 | 83.029 |
| 982 | 83.079 |
| 980 | 83.069 |
| 978 | 83.098 |
| 976 | 83.139 |
| 974 | 83.155 |
| 972 | 83.229 |
| 970 | 83.25 |
| 968 | 83.255 |
| 966 | 83.259 |
| 964 | 83.308 |
| 962 | 83.33 |
| 960 | 83.356 |
| 958 | 83.383 |
| 956 | 83.391 |
| 954 | 83.434 |
| 952 | 83.505 |
| 950 | 83.511 |
| 948 | 83.584 |
| 946 | 83.602 |
| 944 | 83.583 |
| 942 | 83.598 |
| 940 | 83.56 |
| 938 | 83.566 |
| 936 | 83.608 |
| 934 | 83.679 |
| 932 | 83.681 |
| 930 | 83.7 |
| 928 | 83.785 |
| 926 | 83.855 |
| 924 | 83.952 |
| 922 | 84.024 |
| 920 | 84.04 |
| 918 | 84.019 |
| 916 | 84.024 |
| 914 | 84.031 |
| 912 | 83.969 |
| 910 | 84.029 |
| 908 | 83.992 |
| 906 | 84.01 |
| 904 | 84.109 |
| 902 | 84.114 |
| 900 | 84.241 |
| 898 | 84.291 |
| 896 | 84.272 |
| 894 | 84.324 |
| 892 | 84.367 |
| 890 | 84.444 |
| 888 | 84.46 |
| 886 | 84.403 |
| 884 | 84.381 |
| 882 | 84.425 |
| 880 | 84.521 |
| 878 | 84.69 |
| 876 | 84.716 |
| 874 | 84.669 |
| 872 | 84.722 |
| 870 | 84.646 |
| 868 | 84.687 |
| 866 | 84.77 |
| 864 | 84.791 |
| 862 | 84.912 |
| 860 | 85.078 |
| 858 | 85.061 |
| 856 | 85.173 |
| 854 | 85.053 |
| 852 | 85.177 |
| 850 | 85.244 |
| 848 | 85.278 |
| 846 | 85.312 |
| 844 | 85.103 |
| 842 | 85.084 |
| 840 | 85.181 |
| 838 | 85.392 |
| 836 | 85.704 |
| 834 | 85.821 |
| 832 | 85.84 |
| 830 | 85.768 |
| 828 | 85.722 |
| 826 | 85.722 |
| 824 | 85.803 |
| 822 | 85.794 |
| 820 | 85.963 |
| 818 | 85.966 |
| 816 | 85.852 |
| 814 | 86.004 |
| 812 | 85.918 |
| 810 | 85.949 |
| 808 | 86.041 |
| 806 | 86.048 |
| 804 | 85.974 |
| 802 | 85.982 |
| 800 | 85.933 |
| 798 | 85.856 |
| 796 | 85.935 |
| 794 | 86.139 |
| 792 | 86.223 |
| 790 | 86.199 |
| 788 | 86.303 |
| 786 | 86.168 |
| 784 | 86.098 |
| 782 | 86.321 |
| 780 | 86.377 |
| 778 | 86.425 |
| 776 | 86.426 |
| 774 | 86.391 |
| 772 | 86.299 |
| 770 | 86.349 |
| 768 | 86.429 |
| 766 | 86.383 |
| 764 | 86.504 |
| 762 | 86.413 |
| 760 | 86.451 |
| 758 | 86.47 |
| 756 | 86.447 |
| 754 | 86.505 |
| 752 | 86.53 |
| 750 | 86.516 |
| 748 | 86.622 |
| 746 | 86.707 |
| 744 | 86.812 |
| 742 | 86.774 |
| 740 | 86.753 |
| 738 | 86.75 |
| 736 | 86.778 |
| 734 | 86.84 |
| 732 | 86.779 |
| 730 | 86.842 |
| 728 | 86.901 |
| 726 | 86.87 |
| 724 | 86.871 |
| 722 | 86.846 |
| 720 | 86.878 |
| 718 | 86.964 |
| 716 | 87.061 |
| 714 | 87.096 |
| 712 | 87.144 |
| 710 | 87.172 |
| 708 | 87.099 |
| 706 | 87.171 |
| 704 | 87.203 |
| 702 | 87.15 |
| 700 | 87.289 |
| 698 | 87.292 |
| 696 | 87.32 |
| 694 | 87.405 |
| 692 | 87.427 |
| 690 | 87.637 |
| 688 | 87.59 |
| 686 | 87.606 |
| 684 | 87.598 |
| 682 | 87.469 |
| 680 | 87.53 |
| 678 | 87.595 |
| 676 | 87.592 |
| 674 | 87.683 |
| 672 | 87.741 |
| 670 | 87.73 |
| 668 | 87.891 |
| 666 | 87.934 |
| 664 | 87.927 |
| 662 | 87.984 |
| 660 | 87.988 |
| 658 | 88.002 |
| 656 | 88.111 |
| 654 | 88.121 |
| 652 | 88.177 |
| 650 | 88.194 |
| 648 | 88.245 |
| 646 | 88.329 |
| 644 | 88.339 |
| 642 | 88.348 |
| 640 | 88.338 |
| 638 | 88.441 |
| 636 | 88.409 |
| 634 | 88.448 |
| 632 | 88.497 |
| 630 | 88.494 |
| 628 | 88.549 |
| 626 | 88.558 |
| 624 | 88.545 |
| 622 | 88.639 |
| 620 | 88.649 |
| 618 | 88.678 |
| 616 | 88.78 |
| 614 | 88.783 |
| 612 | 88.782 |
| 610 | 88.832 |
| 608 | 88.767 |
| 606 | 88.657 |
| 604 | 88.83 |
| 602 | 88.873 |
| 600 | 88.973 |
| 598 | 89.038 |
| 596 | 88.951 |
| 594 | 88.949 |
| 592 | 89.011 |
| 590 | 89.071 |
| 588 | 89.166 |
| 586 | 89.327 |
| 584 | 89.354 |
| 582 | 89.369 |
| 580 | 89.333 |
| 578 | 89.311 |
| 576 | 89.361 |
| 574 | 89.335 |
| 572 | 89.414 |
| 570 | 89.471 |
| 568 | 89.418 |
| 566 | 89.517 |
| 564 | 89.632 |
| 562 | 89.707 |
| 560 | 89.81 |
| 558 | 89.862 |
| 556 | 89.823 |
| 554 | 89.839 |
| 552 | 89.911 |
| 550 | 90.066 |
| 548 | 90.098 |
| 546 | 90.127 |
| 544 | 90.17 |
| 542 | 90.1 |
| 540 | 90.255 |
| 538 | 90.357 |
| 536 | 90.384 |
| 534 | 90.522 |
| 532 | 90.655 |
| 530 | 90.706 |
| 528 | 90.765 |
| 526 | 90.78 |
| 524 | 90.714 |
| 522 | 90.879 |
| 520 | 90.891 |
| 518 | 90.948 |
| 516 | 91.01 |
| 514 | 91.003 |
| 512 | 91.089 |
| 510 | 91.206 |
| 508 | 91.144 |
| 506 | 91.325 |
| 504 | 91.408 |
| 502 | 91.49 |
| 500 | 91.644 |
| 498 | 91.81 |
| 496 | 91.868 |
| 494 | 91.898 |
| 492 | 91.932 |
| 490 | 91.945 |
| 488 | 92.033 |
| 486 | 92.101 |
| 484 | 92.186 |
| 482 | 92.195 |
| 480 | 92.245 |
| 478 | 92.356 |
| 476 | 92.45 |
| 474 | 92.598 |
| 472 | 92.701 |
| 470 | 92.745 |
| 468 | 92.838 |
| 466 | 92.885 |
| 464 | 92.964 |
| 462 | 92.962 |
| 460 | 92.998 |
| 458 | 93.103 |
| 456 | 93.201 |
| 454 | 93.295 |
| 452 | 93.335 |
| 450 | 93.221 |
| 448 | 93.205 |
| 446 | 93.31 |
| 444 | 93.403 |
| 442 | 93.418 |
| 440 | 93.53 |
| 438 | 93.661 |
| 436 | 93.654 |
| 434 | 93.79 |
| 432 | 93.708 |
| 430 | 93.596 |
| 428 | 93.64 |
| 426 | 93.594 |
| 424 | 92.831 |
| 422 | 92.03 |
| 420 | 91.401 |
| 418 | 90.718 |
| 416 | 89.572 |
| 414 | 87.86 |
| 412 | 85.471 |
| 410 | 82.719 |
| 408 | 79.647 |
| 406 | 76.276 |
| 404 | 72.608 |
| 402 | 69.154 |
| 400 | 65.657 |
| 398 | 62.627 |
| 396 | 59.93 |
| 394 | 57.536 |
| 392 | 55.545 |
| 390 | 53.69 |
| 388 | 52.189 |
| 386 | 50.822 |
| 384 | 49.667 |
| 382 | 48.637 |
| 380 | 47.839 |
| 378 | 47.284 |
| 376 | 46.725 |
| 374 | 46.063 |
| 372 | 45.787 |
| 370 | 45.378 |
| 368 | 45.294 |
| 366 | 45.247 |
| 364 | 45.097 |
| 362 | 45.126 |
| 360 | 44.813 |
| 358 | 44.622 |
| 356 | 44.45 |
| 354 | 44.133 |
| 352 | 43.968 |
| 350 | 43.781 |
| 348 | 43.544 |
| 346 | 43.347 |
| 344 | 42.654 |
| 342 | 41.968 |
| 340 | 42.078 |
| 338 | 41.967 |
| 336 | 41.321 |
| 334 | 41.2 |
| 332 | 40.804 |
| 330 | 40.354 |
| 328 | 40.042 |
| 326 | 39.239 |
| 324 | 38.698 |
| 322 | 38.541 |
| 320 | 38.258 |
| 318 | 37.857 |
| 316 | 37.709 |
| 314 | 37.135 |
| 312 | 36.796 |
| 310 | 36.371 |
| 308 | 35.742 |
| 306 | 35.028 |
| 304 | 34.351 |
| 302 | 33.653 |
| 300 | 32.949 |
| 298 | 32.427 |
| 296 | 32.005 |
| 294 | 31.779 |
| 292 | 31.528 |
| 290 | 31.555 |
| 288 | 31.439 |
| 286 | 31.447 |
| 284 | 31.302 |
| 282 | 31.298 |
| 280 | 30.978 |
| 278 | 30.553 |
| 276 | 30.524 |
| 274 | 30.105 |
| 272 | 30.24 |
| 270 | 30.38 |
| 268 | 30.403 |
| 266 | 30.347 |
| 264 | 30.375 |
| 262 | 30.589 |
| 260 | 30.577 |
| 258 | 30.97 |
| 256 | 31.336 |
| 254 | 31.617 |
| 252 | 32.143 |
| 250 | 32.779 |
| 248 | 33.275 |
| 246 | 33.965 |
| 244 | 34.75 |
| 242 | 35.187 |
| 240 | 36.308 |
| 238 | 37.174 |
| 236 | 38.372 |
| 234 | 39.904 |
| 232 | 41.005 |
| 230 | 42.425 |
| 228 | 43.755 |
| 226 | 45.523 |
| 224 | 45.704 |
| 222 | 46.462 |
| 220 | 46.844 |
| 218 | 47.606 |
| 216 | 49.181 |
| 214 | 47.636 |
| 212 | 48.704 |
| 210 | 48.94 |
| 208 | 50.504 |
| 206 | 54.303 |
| 204 | 58.482 |
| 202 | 54.446 |
| 200 | 55.435 |
| 198 | 58.59 |
| 196 | 50.904 |
| 194 | 85.575 |
| 192 | 80.457 |
| 190 | 62.048 |
