# Supplementary material for: Advancing energy storage and supercapacitor applications through the development of Li+-doped MgTiO3 perovskite nano-ceramics
Source: Sci Rep. 2024 Jan 22;14:1849. doi: 10.1038/s41598-024-52262-6 (PMC10803294; doi:10.1038/s41598-024-52262-6)
Supplement: Supplementary file 3 — Supplementary Information 3. [file 41598_2024_52262_MOESM3_ESM.docx]

Diffuse reflectance (Sample: MT10Li)

| TITLE | MT10Li |
| --- | --- |
| DATA TYPE | |
| ORIGIN | JASCO |
| OWNER |  |
| DATE | 23/05/22 |
| TIME | 11:06:49 |
| SPECTROMETER/DATA SYSTEM | JASCO Corp., V-570, Rev. 1.00 |
| RESOLUTION | |
| DELTAX | -2 |
| XUNITS | NANOMETERS |
| YUNITS | REFLECTANCE |
| FIRSTX | 2500 |
| LASTX | 190 |
| NPOINTS | 1156 |
| FIRSTY | 0.80579 |
| MAXY | 0.99317 |
| MINY | 0.2994 |
| XYDATA |  |
| 2500 | 80.579 |
| 2498 | 78.274 |
| 2496 | 78.295 |
| 2494 | 75.865 |
| 2492 | 76.299 |
| 2490 | 76.693 |
| 2488 | 76.959 |
| 2486 | 77.347 |
| 2484 | 76.619 |
| 2482 | 76.165 |
| 2480 | 76.762 |
| 2478 | 76.663 |
| 2476 | 78.863 |
| 2474 | 79.911 |
| 2472 | 79.359 |
| 2470 | 81.457 |
| 2468 | 80.031 |
| 2466 | 78.892 |
| 2464 | 78.527 |
| 2462 | 77.686 |
| 2460 | 77.957 |
| 2458 | 77.92 |
| 2456 | 78.299 |
| 2454 | 79.374 |
| 2452 | 79.171 |
| 2450 | 79.88 |
| 2448 | 79.634 |
| 2446 | 79.731 |
| 2444 | 80.238 |
| 2442 | 80.152 |
| 2440 | 80.03 |
| 2438 | 78.605 |
| 2436 | 78.249 |
| 2434 | 78.037 |
| 2432 | 79.035 |
| 2430 | 78.956 |
| 2428 | 79.458 |
| 2426 | 78.49 |
| 2424 | 78.03 |
| 2422 | 78.367 |
| 2420 | 76.84 |
| 2418 | 77.525 |
| 2416 | 76.858 |
| 2414 | 77.651 |
| 2412 | 77.846 |
| 2410 | 76.824 |
| 2408 | 77.316 |
| 2406 | 76.92 |
| 2404 | 77.335 |
| 2402 | 78.37 |
| 2400 | 79.315 |
| 2398 | 79.503 |
| 2396 | 79.357 |
| 2394 | 79.18 |
| 2392 | 78.901 |
| 2390 | 79.297 |
| 2388 | 79.603 |
| 2386 | 80.139 |
| 2384 | 80.026 |
| 2382 | 78.835 |
| 2380 | 79.795 |
| 2378 | 79.801 |
| 2376 | 79.676 |
| 2374 | 79.538 |
| 2372 | 78.929 |
| 2370 | 78.315 |
| 2368 | 78.347 |
| 2366 | 78.468 |
| 2364 | 78.77 |
| 2362 | 78.587 |
| 2360 | 78.925 |
| 2358 | 79.047 |
| 2356 | 79.082 |
| 2354 | 79.759 |
| 2352 | 80.016 |
| 2350 | 80.645 |
| 2348 | 80.098 |
| 2346 | 80.772 |
| 2344 | 80.224 |
| 2342 | 80.064 |
| 2340 | 80.782 |
| 2338 | 80.566 |
| 2336 | 79.764 |
| 2334 | 78.898 |
| 2332 | 78.552 |
| 2330 | 77.796 |
| 2328 | 78.825 |
| 2326 | 79.219 |
| 2324 | 78.963 |
| 2322 | 78.969 |
| 2320 | 78.714 |
| 2318 | 78.806 |
| 2316 | 78.66 |
| 2314 | 78.873 |
| 2312 | 79.342 |
| 2310 | 79.424 |
| 2308 | 79.606 |
| 2306 | 79.469 |
| 2304 | 78.74 |
| 2302 | 78.274 |
| 2300 | 78.397 |
| 2298 | 78.083 |
| 2296 | 78.182 |
| 2294 | 78.676 |
| 2292 | 78.103 |
| 2290 | 78.003 |
| 2288 | 78.425 |
| 2286 | 78.803 |
| 2284 | 78.641 |
| 2282 | 78.302 |
| 2280 | 77.542 |
| 2278 | 77.298 |
| 2276 | 77.763 |
| 2274 | 77.917 |
| 2272 | 77.948 |
| 2270 | 77.487 |
| 2268 | 77.444 |
| 2266 | 78.202 |
| 2264 | 77.959 |
| 2262 | 78.451 |
| 2260 | 79.444 |
| 2258 | 79.116 |
| 2256 | 79.466 |
| 2254 | 79.987 |
| 2252 | 79.067 |
| 2250 | 79.372 |
| 2248 | 79.733 |
| 2246 | 78.981 |
| 2244 | 78.966 |
| 2242 | 78.378 |
| 2240 | 77.972 |
| 2238 | 78.617 |
| 2236 | 78.574 |
| 2234 | 78.566 |
| 2232 | 78.397 |
| 2230 | 77.942 |
| 2228 | 77.543 |
| 2226 | 77.672 |
| 2224 | 77.551 |
| 2222 | 77.44 |
| 2220 | 77.856 |
| 2218 | 77.813 |
| 2216 | 77.93 |
| 2214 | 77.49 |
| 2212 | 77.661 |
| 2210 | 77.658 |
| 2208 | 78.059 |
| 2206 | 78.238 |
| 2204 | 77.56 |
| 2202 | 77.687 |
| 2200 | 77.831 |
| 2198 | 77.646 |
| 2196 | 77.99 |
| 2194 | 78.027 |
| 2192 | 78.18 |
| 2190 | 77.863 |
| 2188 | 78.562 |
| 2186 | 78.453 |
| 2184 | 78.118 |
| 2182 | 79.031 |
| 2180 | 78.611 |
| 2178 | 78.58 |
| 2176 | 79.096 |
| 2174 | 78.91 |
| 2172 | 78.682 |
| 2170 | 79.308 |
| 2168 | 79.718 |
| 2166 | 80.081 |
| 2164 | 80.5 |
| 2162 | 79.893 |
| 2160 | 79.453 |
| 2158 | 79.549 |
| 2156 | 79.86 |
| 2154 | 80.193 |
| 2152 | 79.971 |
| 2150 | 80.218 |
| 2148 | 79.928 |
| 2146 | 80.106 |
| 2144 | 80.775 |
| 2142 | 81.189 |
| 2140 | 81.205 |
| 2138 | 81.306 |
| 2136 | 81.169 |
| 2134 | 80.53 |
| 2132 | 80.702 |
| 2130 | 80.883 |
| 2128 | 81.378 |
| 2126 | 81.517 |
| 2124 | 81.42 |
| 2122 | 81.286 |
| 2120 | 80.546 |
| 2118 | 80.278 |
| 2116 | 80.615 |
| 2114 | 80.6 |
| 2112 | 80.925 |
| 2110 | 81.577 |
| 2108 | 81.071 |
| 2106 | 81.437 |
| 2104 | 81.126 |
| 2102 | 81.182 |
| 2100 | 81.162 |
| 2098 | 80.666 |
| 2096 | 80.927 |
| 2094 | 80.549 |
| 2092 | 80.779 |
| 2090 | 81.019 |
| 2088 | 80.704 |
| 2086 | 80.608 |
| 2084 | 80.144 |
| 2082 | 79.923 |
| 2080 | 80.279 |
| 2078 | 80.149 |
| 2076 | 80.365 |
| 2074 | 80.091 |
| 2072 | 79.305 |
| 2070 | 79.052 |
| 2068 | 79.162 |
| 2066 | 79.471 |
| 2064 | 79.707 |
| 2062 | 79.677 |
| 2060 | 79.133 |
| 2058 | 79.206 |
| 2056 | 79.064 |
| 2054 | 79.448 |
| 2052 | 79.607 |
| 2050 | 79.443 |
| 2048 | 79.483 |
| 2046 | 78.895 |
| 2044 | 79.028 |
| 2042 | 78.671 |
| 2040 | 78.782 |
| 2038 | 79.265 |
| 2036 | 79.142 |
| 2034 | 79.613 |
| 2032 | 79.358 |
| 2030 | 78.612 |
| 2028 | 78.592 |
| 2026 | 78.459 |
| 2024 | 78.683 |
| 2022 | 78.984 |
| 2020 | 79.037 |
| 2018 | 78.41 |
| 2016 | 78.18 |
| 2014 | 78.239 |
| 2012 | 78.325 |
| 2010 | 78.297 |
| 2008 | 78.193 |
| 2006 | 78.11 |
| 2004 | 77.74 |
| 2002 | 77.863 |
| 2000 | 78.06 |
| 1998 | 78.071 |
| 1996 | 78.172 |
| 1994 | 78.423 |
| 1992 | 78.281 |
| 1990 | 78.173 |
| 1988 | 77.809 |
| 1986 | 77.523 |
| 1984 | 77.247 |
| 1982 | 77.2 |
| 1980 | 77.205 |
| 1978 | 76.901 |
| 1976 | 76.802 |
| 1974 | 76.645 |
| 1972 | 76.793 |
| 1970 | 76.954 |
| 1968 | 77.206 |
| 1966 | 77.61 |
| 1964 | 77.415 |
| 1962 | 77.418 |
| 1960 | 77.216 |
| 1958 | 77.134 |
| 1956 | 77.465 |
| 1954 | 77.46 |
| 1952 | 77.422 |
| 1950 | 77.013 |
| 1948 | 76.773 |
| 1946 | 76.412 |
| 1944 | 76.466 |
| 1942 | 76.668 |
| 1940 | 76.638 |
| 1938 | 76.716 |
| 1936 | 76.842 |
| 1934 | 76.674 |
| 1932 | 76.59 |
| 1930 | 76.307 |
| 1928 | 76.297 |
| 1926 | 76.355 |
| 1924 | 76.575 |
| 1922 | 76.952 |
| 1920 | 76.939 |
| 1918 | 76.968 |
| 1916 | 76.602 |
| 1914 | 76.722 |
| 1912 | 76.567 |
| 1910 | 76.397 |
| 1908 | 77.025 |
| 1906 | 77.086 |
| 1904 | 77.469 |
| 1902 | 77.383 |
| 1900 | 77.412 |
| 1898 | 77.336 |
| 1896 | 77.771 |
| 1894 | 77.608 |
| 1892 | 77.968 |
| 1890 | 78.014 |
| 1888 | 77.72 |
| 1886 | 77.708 |
| 1884 | 77.626 |
| 1882 | 77.661 |
| 1880 | 77.897 |
| 1878 | 78.125 |
| 1876 | 78.276 |
| 1874 | 78.482 |
| 1872 | 78.51 |
| 1870 | 78.475 |
| 1868 | 78.57 |
| 1866 | 78.586 |
| 1864 | 78.758 |
| 1862 | 78.755 |
| 1860 | 78.706 |
| 1858 | 78.743 |
| 1856 | 78.827 |
| 1854 | 79.022 |
| 1852 | 78.949 |
| 1850 | 79.227 |
| 1848 | 79.04 |
| 1846 | 79.112 |
| 1844 | 79.471 |
| 1842 | 79.24 |
| 1840 | 79.433 |
| 1838 | 79.388 |
| 1836 | 79.232 |
| 1834 | 79.187 |
| 1832 | 79.433 |
| 1830 | 79.319 |
| 1828 | 79.337 |
| 1826 | 79.466 |
| 1824 | 79.221 |
| 1822 | 79.391 |
| 1820 | 79.326 |
| 1818 | 79.336 |
| 1816 | 79.337 |
| 1814 | 79.344 |
| 1812 | 79.374 |
| 1810 | 79.338 |
| 1808 | 79.48 |
| 1806 | 79.554 |
| 1804 | 79.635 |
| 1802 | 79.683 |
| 1800 | 79.456 |
| 1798 | 79.352 |
| 1796 | 79.282 |
| 1794 | 79.381 |
| 1792 | 79.518 |
| 1790 | 79.496 |
| 1788 | 79.533 |
| 1786 | 79.496 |
| 1784 | 79.482 |
| 1782 | 79.52 |
| 1780 | 79.591 |
| 1778 | 79.557 |
| 1776 | 79.593 |
| 1774 | 79.593 |
| 1772 | 79.456 |
| 1770 | 79.349 |
| 1768 | 79.396 |
| 1766 | 79.46 |
| 1764 | 79.598 |
| 1762 | 79.617 |
| 1760 | 79.594 |
| 1758 | 79.554 |
| 1756 | 79.524 |
| 1754 | 79.576 |
| 1752 | 79.604 |
| 1750 | 79.665 |
| 1748 | 79.673 |
| 1746 | 79.758 |
| 1744 | 79.81 |
| 1742 | 79.868 |
| 1740 | 79.976 |
| 1738 | 80.02 |
| 1736 | 79.952 |
| 1734 | 80.069 |
| 1732 | 80.005 |
| 1730 | 80.043 |
| 1728 | 80.143 |
| 1726 | 80.164 |
| 1724 | 80.273 |
| 1722 | 80.327 |
| 1720 | 80.424 |
| 1718 | 80.314 |
| 1716 | 80.416 |
| 1714 | 80.401 |
| 1712 | 80.437 |
| 1710 | 80.512 |
| 1708 | 80.423 |
| 1706 | 80.484 |
| 1704 | 80.494 |
| 1702 | 80.486 |
| 1700 | 80.604 |
| 1698 | 80.615 |
| 1696 | 80.512 |
| 1694 | 80.652 |
| 1692 | 80.579 |
| 1690 | 80.545 |
| 1688 | 80.685 |
| 1686 | 80.533 |
| 1684 | 80.546 |
| 1682 | 80.54 |
| 1680 | 80.387 |
| 1678 | 80.43 |
| 1676 | 80.492 |
| 1674 | 80.447 |
| 1672 | 80.494 |
| 1670 | 80.43 |
| 1668 | 80.454 |
| 1666 | 80.502 |
| 1664 | 80.424 |
| 1662 | 80.5 |
| 1660 | 80.426 |
| 1658 | 80.473 |
| 1656 | 80.526 |
| 1654 | 80.5 |
| 1652 | 80.373 |
| 1650 | 80.449 |
| 1648 | 80.459 |
| 1646 | 80.418 |
| 1644 | 80.55 |
| 1642 | 80.481 |
| 1640 | 80.439 |
| 1638 | 80.473 |
| 1636 | 80.453 |
| 1634 | 80.592 |
| 1632 | 80.629 |
| 1630 | 80.718 |
| 1628 | 80.734 |
| 1626 | 80.584 |
| 1624 | 80.61 |
| 1622 | 80.588 |
| 1620 | 80.602 |
| 1618 | 80.67 |
| 1616 | 80.739 |
| 1614 | 80.703 |
| 1612 | 80.668 |
| 1610 | 80.62 |
| 1608 | 80.642 |
| 1606 | 80.65 |
| 1604 | 80.738 |
| 1602 | 80.668 |
| 1600 | 80.572 |
| 1598 | 80.654 |
| 1596 | 80.649 |
| 1594 | 80.77 |
| 1592 | 80.864 |
| 1590 | 80.877 |
| 1588 | 80.802 |
| 1586 | 80.847 |
| 1584 | 80.868 |
| 1582 | 80.723 |
| 1580 | 80.758 |
| 1578 | 80.751 |
| 1576 | 80.737 |
| 1574 | 80.843 |
| 1572 | 80.918 |
| 1570 | 80.823 |
| 1568 | 80.876 |
| 1566 | 80.858 |
| 1564 | 80.792 |
| 1562 | 80.892 |
| 1560 | 80.891 |
| 1558 | 80.948 |
| 1556 | 80.945 |
| 1554 | 80.918 |
| 1552 | 80.832 |
| 1550 | 80.753 |
| 1548 | 80.741 |
| 1546 | 80.776 |
| 1544 | 80.887 |
| 1542 | 80.952 |
| 1540 | 80.909 |
| 1538 | 80.906 |
| 1536 | 80.764 |
| 1534 | 80.728 |
| 1532 | 80.885 |
| 1530 | 80.832 |
| 1528 | 80.973 |
| 1526 | 80.996 |
| 1524 | 80.945 |
| 1522 | 80.984 |
| 1520 | 80.9 |
| 1518 | 81.029 |
| 1516 | 80.966 |
| 1514 | 81.021 |
| 1512 | 81.107 |
| 1510 | 81.048 |
| 1508 | 81.078 |
| 1506 | 81.005 |
| 1504 | 81.01 |
| 1502 | 80.993 |
| 1500 | 81.142 |
| 1498 | 81.123 |
| 1496 | 81.086 |
| 1494 | 81.07 |
| 1492 | 80.937 |
| 1490 | 80.961 |
| 1488 | 81.029 |
| 1486 | 81.071 |
| 1484 | 81.026 |
| 1482 | 81.044 |
| 1480 | 80.946 |
| 1478 | 80.905 |
| 1476 | 80.992 |
| 1474 | 80.947 |
| 1472 | 80.93 |
| 1470 | 81.043 |
| 1468 | 80.931 |
| 1466 | 80.99 |
| 1464 | 81.045 |
| 1462 | 81.009 |
| 1460 | 81.16 |
| 1458 | 81.164 |
| 1456 | 81.182 |
| 1454 | 81.067 |
| 1452 | 81.012 |
| 1450 | 80.938 |
| 1448 | 80.927 |
| 1446 | 80.979 |
| 1444 | 81.007 |
| 1442 | 81.064 |
| 1440 | 81.042 |
| 1438 | 80.998 |
| 1436 | 81.01 |
| 1434 | 80.991 |
| 1432 | 81.072 |
| 1430 | 81.13 |
| 1428 | 81.109 |
| 1426 | 81.13 |
| 1424 | 81.127 |
| 1422 | 81.111 |
| 1420 | 81.128 |
| 1418 | 81.164 |
| 1416 | 81.177 |
| 1414 | 81.216 |
| 1412 | 81.247 |
| 1410 | 81.283 |
| 1408 | 81.213 |
| 1406 | 81.168 |
| 1404 | 81.172 |
| 1402 | 81.152 |
| 1400 | 81.266 |
| 1398 | 81.407 |
| 1396 | 81.459 |
| 1394 | 81.534 |
| 1392 | 81.486 |
| 1390 | 81.506 |
| 1388 | 81.531 |
| 1386 | 81.577 |
| 1384 | 81.675 |
| 1382 | 81.635 |
| 1380 | 81.62 |
| 1378 | 81.52 |
| 1376 | 81.434 |
| 1374 | 81.459 |
| 1372 | 81.5 |
| 1370 | 81.622 |
| 1368 | 81.706 |
| 1366 | 81.749 |
| 1364 | 81.704 |
| 1362 | 81.685 |
| 1360 | 81.72 |
| 1358 | 81.789 |
| 1356 | 81.902 |
| 1354 | 81.952 |
| 1352 | 81.943 |
| 1350 | 81.949 |
| 1348 | 81.914 |
| 1346 | 81.936 |
| 1344 | 81.959 |
| 1342 | 81.945 |
| 1340 | 82.004 |
| 1338 | 81.972 |
| 1336 | 81.954 |
| 1334 | 81.877 |
| 1332 | 81.844 |
| 1330 | 81.827 |
| 1328 | 81.857 |
| 1326 | 81.992 |
| 1324 | 82.019 |
| 1322 | 82.067 |
| 1320 | 82.031 |
| 1318 | 81.999 |
| 1316 | 81.958 |
| 1314 | 81.99 |
| 1312 | 82.026 |
| 1310 | 82.035 |
| 1308 | 82.117 |
| 1306 | 82.078 |
| 1304 | 82.094 |
| 1302 | 82.099 |
| 1300 | 82.062 |
| 1298 | 82.115 |
| 1296 | 82.146 |
| 1294 | 82.13 |
| 1292 | 82.126 |
| 1290 | 82.119 |
| 1288 | 82.125 |
| 1286 | 82.18 |
| 1284 | 82.231 |
| 1282 | 82.265 |
| 1280 | 82.231 |
| 1278 | 82.206 |
| 1276 | 82.192 |
| 1274 | 82.159 |
| 1272 | 82.159 |
| 1270 | 82.186 |
| 1268 | 82.246 |
| 1266 | 82.298 |
| 1264 | 82.374 |
| 1262 | 82.399 |
| 1260 | 82.362 |
| 1258 | 82.364 |
| 1256 | 82.339 |
| 1254 | 82.316 |
| 1252 | 82.36 |
| 1250 | 82.338 |
| 1248 | 82.369 |
| 1246 | 82.366 |
| 1244 | 82.366 |
| 1242 | 82.411 |
| 1240 | 82.422 |
| 1238 | 82.51 |
| 1236 | 82.504 |
| 1234 | 82.534 |
| 1232 | 82.575 |
| 1230 | 82.535 |
| 1228 | 82.579 |
| 1226 | 82.595 |
| 1224 | 82.611 |
| 1222 | 82.679 |
| 1220 | 82.675 |
| 1218 | 82.596 |
| 1216 | 82.615 |
| 1214 | 82.54 |
| 1212 | 82.554 |
| 1210 | 82.621 |
| 1208 | 82.621 |
| 1206 | 82.582 |
| 1204 | 82.518 |
| 1202 | 82.589 |
| 1200 | 82.613 |
| 1198 | 82.7 |
| 1196 | 82.738 |
| 1194 | 82.754 |
| 1192 | 82.725 |
| 1190 | 82.716 |
| 1188 | 82.711 |
| 1186 | 82.742 |
| 1184 | 82.758 |
| 1182 | 82.806 |
| 1180 | 82.842 |
| 1178 | 82.842 |
| 1176 | 82.868 |
| 1174 | 82.88 |
| 1172 | 82.862 |
| 1170 | 82.857 |
| 1168 | 82.847 |
| 1166 | 82.829 |
| 1164 | 82.814 |
| 1162 | 82.854 |
| 1160 | 82.846 |
| 1158 | 82.861 |
| 1156 | 82.846 |
| 1154 | 82.803 |
| 1152 | 82.784 |
| 1150 | 82.798 |
| 1148 | 82.854 |
| 1146 | 82.893 |
| 1144 | 82.935 |
| 1142 | 82.913 |
| 1140 | 82.868 |
| 1138 | 82.901 |
| 1136 | 82.943 |
| 1134 | 82.995 |
| 1132 | 83.029 |
| 1130 | 82.976 |
| 1128 | 82.939 |
| 1126 | 82.845 |
| 1124 | 82.86 |
| 1122 | 82.881 |
| 1120 | 82.902 |
| 1118 | 82.969 |
| 1116 | 82.947 |
| 1114 | 82.983 |
| 1112 | 82.966 |
| 1110 | 82.978 |
| 1108 | 83.009 |
| 1106 | 82.975 |
| 1104 | 83.035 |
| 1102 | 83.044 |
| 1100 | 83.038 |
| 1098 | 83.068 |
| 1096 | 83.064 |
| 1094 | 83.097 |
| 1092 | 83.088 |
| 1090 | 83.101 |
| 1088 | 83.096 |
| 1086 | 83.09 |
| 1084 | 83.091 |
| 1082 | 83.104 |
| 1080 | 83.139 |
| 1078 | 83.167 |
| 1076 | 83.202 |
| 1074 | 83.169 |
| 1072 | 83.14 |
| 1070 | 83.132 |
| 1068 | 83.139 |
| 1066 | 83.226 |
| 1064 | 83.231 |
| 1062 | 83.241 |
| 1060 | 83.259 |
| 1058 | 83.25 |
| 1056 | 83.278 |
| 1054 | 83.307 |
| 1052 | 83.286 |
| 1050 | 83.296 |
| 1048 | 83.297 |
| 1046 | 83.312 |
| 1044 | 83.354 |
| 1042 | 83.364 |
| 1040 | 83.383 |
| 1038 | 83.356 |
| 1036 | 83.364 |
| 1034 | 83.384 |
| 1032 | 83.455 |
| 1030 | 83.531 |
| 1028 | 83.568 |
| 1026 | 83.57 |
| 1024 | 83.492 |
| 1022 | 83.466 |
| 1020 | 83.466 |
| 1018 | 83.488 |
| 1016 | 83.592 |
| 1014 | 83.62 |
| 1012 | 83.695 |
| 1010 | 83.692 |
| 1008 | 83.737 |
| 1006 | 83.792 |
| 1004 | 83.791 |
| 1002 | 83.894 |
| 1000 | 83.847 |
| 998 | 83.857 |
| 996 | 83.816 |
| 994 | 83.767 |
| 992 | 83.813 |
| 990 | 83.842 |
| 988 | 83.961 |
| 986 | 84.048 |
| 984 | 84.071 |
| 982 | 84.079 |
| 980 | 84.069 |
| 978 | 84.071 |
| 976 | 84.145 |
| 974 | 84.194 |
| 972 | 84.201 |
| 970 | 84.219 |
| 968 | 84.22 |
| 966 | 84.225 |
| 964 | 84.335 |
| 962 | 84.397 |
| 960 | 84.345 |
| 958 | 84.414 |
| 956 | 84.394 |
| 954 | 84.438 |
| 952 | 84.565 |
| 950 | 84.612 |
| 948 | 84.719 |
| 946 | 84.759 |
| 944 | 84.789 |
| 942 | 84.761 |
| 940 | 84.705 |
| 938 | 84.717 |
| 936 | 84.75 |
| 934 | 84.838 |
| 932 | 84.874 |
| 930 | 84.9 |
| 928 | 84.988 |
| 926 | 85 |
| 924 | 85.071 |
| 922 | 85.141 |
| 920 | 85.148 |
| 918 | 85.256 |
| 916 | 85.305 |
| 914 | 85.289 |
| 912 | 85.309 |
| 910 | 85.276 |
| 908 | 85.22 |
| 906 | 85.269 |
| 904 | 85.336 |
| 902 | 85.37 |
| 900 | 85.576 |
| 898 | 85.634 |
| 896 | 85.693 |
| 894 | 85.694 |
| 892 | 85.726 |
| 890 | 85.843 |
| 888 | 85.853 |
| 886 | 85.911 |
| 884 | 85.786 |
| 882 | 85.738 |
| 880 | 85.788 |
| 878 | 86 |
| 876 | 86.066 |
| 874 | 86.154 |
| 872 | 86.287 |
| 870 | 86.153 |
| 868 | 86.26 |
| 866 | 86.243 |
| 864 | 86.176 |
| 862 | 86.39 |
| 860 | 86.511 |
| 858 | 86.562 |
| 856 | 86.572 |
| 854 | 86.525 |
| 852 | 86.488 |
| 850 | 86.76 |
| 848 | 86.805 |
| 846 | 86.934 |
| 844 | 86.989 |
| 842 | 86.792 |
| 840 | 86.93 |
| 838 | 86.881 |
| 836 | 87.068 |
| 834 | 87.321 |
| 832 | 87.291 |
| 830 | 87.33 |
| 828 | 87.282 |
| 826 | 87.265 |
| 824 | 87.154 |
| 822 | 87.282 |
| 820 | 87.312 |
| 818 | 87.263 |
| 816 | 87.322 |
| 814 | 87.446 |
| 812 | 87.532 |
| 810 | 87.636 |
| 808 | 87.719 |
| 806 | 87.731 |
| 804 | 87.578 |
| 802 | 87.639 |
| 800 | 87.784 |
| 798 | 87.79 |
| 796 | 87.878 |
| 794 | 87.987 |
| 792 | 87.95 |
| 790 | 87.902 |
| 788 | 88.024 |
| 786 | 87.939 |
| 784 | 87.828 |
| 782 | 87.949 |
| 780 | 88.043 |
| 778 | 88.19 |
| 776 | 88.309 |
| 774 | 88.326 |
| 772 | 88.195 |
| 770 | 88.131 |
| 768 | 88.248 |
| 766 | 88.296 |
| 764 | 88.408 |
| 762 | 88.338 |
| 760 | 88.365 |
| 758 | 88.318 |
| 756 | 88.277 |
| 754 | 88.367 |
| 752 | 88.337 |
| 750 | 88.328 |
| 748 | 88.441 |
| 746 | 88.518 |
| 744 | 88.637 |
| 742 | 88.542 |
| 740 | 88.47 |
| 738 | 88.539 |
| 736 | 88.64 |
| 734 | 88.694 |
| 732 | 88.805 |
| 730 | 88.824 |
| 728 | 88.755 |
| 726 | 88.735 |
| 724 | 88.7 |
| 722 | 88.662 |
| 720 | 88.688 |
| 718 | 88.745 |
| 716 | 88.754 |
| 714 | 88.863 |
| 712 | 88.95 |
| 710 | 89.002 |
| 708 | 88.948 |
| 706 | 88.961 |
| 704 | 88.989 |
| 702 | 88.979 |
| 700 | 89.083 |
| 698 | 89.097 |
| 696 | 89.134 |
| 694 | 89.153 |
| 692 | 89.215 |
| 690 | 89.422 |
| 688 | 89.429 |
| 686 | 89.401 |
| 684 | 89.381 |
| 682 | 89.233 |
| 680 | 89.159 |
| 678 | 89.238 |
| 676 | 89.234 |
| 674 | 89.383 |
| 672 | 89.489 |
| 670 | 89.433 |
| 668 | 89.602 |
| 666 | 89.646 |
| 664 | 89.591 |
| 662 | 89.669 |
| 660 | 89.718 |
| 658 | 89.668 |
| 656 | 89.826 |
| 654 | 89.898 |
| 652 | 89.874 |
| 650 | 89.915 |
| 648 | 89.984 |
| 646 | 90.013 |
| 644 | 90.003 |
| 642 | 89.982 |
| 640 | 89.945 |
| 638 | 90.106 |
| 636 | 90.152 |
| 634 | 90.228 |
| 632 | 90.324 |
| 630 | 90.292 |
| 628 | 90.312 |
| 626 | 90.37 |
| 624 | 90.337 |
| 622 | 90.444 |
| 620 | 90.492 |
| 618 | 90.495 |
| 616 | 90.579 |
| 614 | 90.488 |
| 612 | 90.542 |
| 610 | 90.653 |
| 608 | 90.557 |
| 606 | 90.567 |
| 604 | 90.669 |
| 602 | 90.715 |
| 600 | 90.82 |
| 598 | 90.826 |
| 596 | 90.819 |
| 594 | 90.806 |
| 592 | 90.901 |
| 590 | 91.077 |
| 588 | 91.163 |
| 586 | 91.287 |
| 584 | 91.377 |
| 582 | 91.372 |
| 580 | 91.34 |
| 578 | 91.342 |
| 576 | 91.373 |
| 574 | 91.443 |
| 572 | 91.609 |
| 570 | 91.633 |
| 568 | 91.716 |
| 566 | 91.829 |
| 564 | 91.918 |
| 562 | 92.036 |
| 560 | 92.116 |
| 558 | 92.205 |
| 556 | 92.18 |
| 554 | 92.241 |
| 552 | 92.378 |
| 550 | 92.526 |
| 548 | 92.596 |
| 546 | 92.727 |
| 544 | 92.813 |
| 542 | 92.771 |
| 540 | 92.955 |
| 538 | 93.069 |
| 536 | 93.158 |
| 534 | 93.267 |
| 532 | 93.378 |
| 530 | 93.472 |
| 528 | 93.521 |
| 526 | 93.679 |
| 524 | 93.805 |
| 522 | 94.014 |
| 520 | 94.184 |
| 518 | 94.213 |
| 516 | 94.279 |
| 514 | 94.357 |
| 512 | 94.393 |
| 510 | 94.587 |
| 508 | 94.659 |
| 506 | 94.879 |
| 504 | 95.043 |
| 502 | 95.177 |
| 500 | 95.354 |
| 498 | 95.466 |
| 496 | 95.587 |
| 494 | 95.709 |
| 492 | 95.844 |
| 490 | 95.936 |
| 488 | 96.157 |
| 486 | 96.258 |
| 484 | 96.319 |
| 482 | 96.42 |
| 480 | 96.515 |
| 478 | 96.719 |
| 476 | 96.927 |
| 474 | 97.161 |
| 472 | 97.267 |
| 470 | 97.347 |
| 468 | 97.42 |
| 466 | 97.538 |
| 464 | 97.672 |
| 462 | 97.631 |
| 460 | 97.883 |
| 458 | 98.002 |
| 456 | 97.944 |
| 454 | 98.17 |
| 452 | 98.107 |
| 450 | 98.091 |
| 448 | 98.307 |
| 446 | 98.345 |
| 444 | 98.572 |
| 442 | 98.672 |
| 440 | 98.84 |
| 438 | 98.952 |
| 436 | 98.934 |
| 434 | 98.931 |
| 432 | 98.884 |
| 430 | 99.05 |
| 428 | 99.216 |
| 426 | 99.317 |
| 424 | 98.926 |
| 422 | 98.691 |
| 420 | 99.016 |
| 418 | 98.944 |
| 416 | 98.671 |
| 414 | 98.463 |
| 412 | 98.021 |
| 410 | 97.757 |
| 408 | 97.268 |
| 406 | 96.663 |
| 404 | 95.684 |
| 402 | 94.743 |
| 400 | 93.583 |
| 398 | 92.506 |
| 396 | 91.57 |
| 394 | 90.438 |
| 392 | 89.607 |
| 390 | 88.771 |
| 388 | 88.177 |
| 386 | 87.598 |
| 384 | 87.135 |
| 382 | 86.494 |
| 380 | 85.912 |
| 378 | 85.534 |
| 376 | 85.078 |
| 374 | 84.176 |
| 372 | 83.832 |
| 370 | 83.221 |
| 368 | 83.049 |
| 366 | 82.992 |
| 364 | 82.67 |
| 362 | 82.188 |
| 360 | 81.627 |
| 358 | 81.128 |
| 356 | 80.674 |
| 354 | 80.03 |
| 352 | 79.3 |
| 350 | 77.917 |
| 348 | 76.803 |
| 346 | 75.527 |
| 344 | 73.244 |
| 342 | 70.67 |
| 340 | 70.124 |
| 338 | 69.399 |
| 336 | 67.655 |
| 334 | 66.07 |
| 332 | 63.865 |
| 330 | 61.741 |
| 328 | 59.478 |
| 326 | 57.362 |
| 324 | 55.479 |
| 322 | 53.829 |
| 320 | 52.21 |
| 318 | 50.068 |
| 316 | 48.051 |
| 314 | 45.756 |
| 312 | 43.692 |
| 310 | 41.544 |
| 308 | 39.581 |
| 306 | 37.572 |
| 304 | 35.649 |
| 302 | 34.204 |
| 300 | 33.012 |
| 298 | 32.053 |
| 296 | 31.366 |
| 294 | 30.953 |
| 292 | 30.43 |
| 290 | 30.351 |
| 288 | 30.328 |
| 286 | 30.151 |
| 284 | 30.069 |
| 282 | 30.14 |
| 280 | 29.948 |
| 278 | 29.94 |
| 276 | 30.127 |
| 274 | 30.074 |
| 272 | 30.298 |
| 270 | 30.593 |
| 268 | 30.629 |
| 266 | 30.911 |
| 264 | 31.265 |
| 262 | 31.526 |
| 260 | 31.93 |
| 258 | 32.088 |
| 256 | 32.49 |
| 254 | 32.95 |
| 252 | 33.222 |
| 250 | 33.86 |
| 248 | 34.569 |
| 246 | 35.307 |
| 244 | 36.445 |
| 242 | 37.291 |
| 240 | 38.003 |
| 238 | 38.798 |
| 236 | 40.08 |
| 234 | 41.866 |
| 232 | 43.098 |
| 230 | 45.364 |
| 228 | 45.916 |
| 226 | 47.295 |
| 224 | 47.84 |
| 222 | 47.391 |
| 220 | 48.581 |
| 218 | 47.734 |
| 216 | 48.058 |
| 214 | 47.428 |
| 212 | 47.717 |
| 210 | 50.083 |
| 208 | 52.826 |
| 206 | 55.104 |
| 204 | 51.98 |
| 202 | 54.395 |
| 200 | 58.01 |
| 198 | 58.677 |
| 196 | 56.616 |
| 194 | 58.151 |
| 192 | 58.853 |
| 190 | 66.852 |
