# Supplementary material for: Advancing energy storage and supercapacitor applications through the development of Li+-doped MgTiO3 perovskite nano-ceramics
Source: Sci Rep. 2024 Jan 22;14:1849. doi: 10.1038/s41598-024-52262-6 (PMC10803294; doi:10.1038/s41598-024-52262-6)
Supplement: Supplementary file 5 — Supplementary Information 5. [file 41598_2024_52262_MOESM5_ESM.docx]

Diffuse reflectance (Sample: MT1Li)

| TITLE | MT1Li |
| --- | --- |
| DATA TYPE | |
| ORIGIN | JASCO |
| OWNER |  |
| DATE | 23/05/22 |
| TIME | 10:24:55 |
| SPECTROMETER/DATA SYSTEM | JASCO Corp., V-570, Rev. 1.00 |
| RESOLUTION | |
| DELTAX | -2 |
| XUNITS | NANOMETERS |
| YUNITS | REFLECTANCE |
| FIRSTX | 2500 |
| LASTX | 190 |
| NPOINTS | 1156 |
| FIRSTY | 0.81801 |
| MAXY | 1.0132 |
| MINY | 0.29356 |
| XYDATA |  |
| 2500 | 81.801 |
| 2498 | 79.193 |
| 2496 | 81.406 |
| 2494 | 80.304 |
| 2492 | 80.97 |
| 2490 | 80.649 |
| 2488 | 78.901 |
| 2486 | 78.024 |
| 2484 | 78.584 |
| 2482 | 78.65 |
| 2480 | 79.304 |
| 2478 | 81.223 |
| 2476 | 80.545 |
| 2474 | 81.87 |
| 2472 | 81.772 |
| 2470 | 81.084 |
| 2468 | 81.514 |
| 2466 | 80.175 |
| 2464 | 79.785 |
| 2462 | 80.178 |
| 2460 | 78.81 |
| 2458 | 78.718 |
| 2456 | 79.56 |
| 2454 | 79.611 |
| 2452 | 79.617 |
| 2450 | 81.227 |
| 2448 | 80.529 |
| 2446 | 80.907 |
| 2444 | 81.755 |
| 2442 | 82.314 |
| 2440 | 82.576 |
| 2438 | 82.58 |
| 2436 | 82.911 |
| 2434 | 81.377 |
| 2432 | 80.316 |
| 2430 | 80.171 |
| 2428 | 80.188 |
| 2426 | 79.917 |
| 2424 | 80.766 |
| 2422 | 80.604 |
| 2420 | 80.576 |
| 2418 | 81.363 |
| 2416 | 81.18 |
| 2414 | 81.805 |
| 2412 | 81.242 |
| 2410 | 80.492 |
| 2408 | 81.366 |
| 2406 | 81.342 |
| 2404 | 82.237 |
| 2402 | 83.3 |
| 2400 | 82.543 |
| 2398 | 81.684 |
| 2396 | 80.03 |
| 2394 | 79.732 |
| 2392 | 80.054 |
| 2390 | 81.422 |
| 2388 | 82.478 |
| 2386 | 83.04 |
| 2384 | 82.864 |
| 2382 | 82.074 |
| 2380 | 83.088 |
| 2378 | 82.472 |
| 2376 | 82.76 |
| 2374 | 83.215 |
| 2372 | 82.438 |
| 2370 | 82.222 |
| 2368 | 81.884 |
| 2366 | 81.235 |
| 2364 | 81.48 |
| 2362 | 82.714 |
| 2360 | 83.131 |
| 2358 | 83.035 |
| 2356 | 83.259 |
| 2354 | 82.642 |
| 2352 | 82.477 |
| 2350 | 82.78 |
| 2348 | 83.105 |
| 2346 | 82.382 |
| 2344 | 82.775 |
| 2342 | 82.647 |
| 2340 | 82.182 |
| 2338 | 83.242 |
| 2336 | 82.176 |
| 2334 | 81.889 |
| 2332 | 82.447 |
| 2330 | 81.713 |
| 2328 | 82.466 |
| 2326 | 83.214 |
| 2324 | 82.203 |
| 2322 | 81.609 |
| 2320 | 81.172 |
| 2318 | 79.916 |
| 2316 | 79.652 |
| 2314 | 80.114 |
| 2312 | 80.534 |
| 2310 | 81.507 |
| 2308 | 81.686 |
| 2306 | 81.934 |
| 2304 | 81.464 |
| 2302 | 81.263 |
| 2300 | 81.485 |
| 2298 | 81.602 |
| 2296 | 81.472 |
| 2294 | 81.555 |
| 2292 | 80.936 |
| 2290 | 80.85 |
| 2288 | 81.476 |
| 2286 | 81.31 |
| 2284 | 81.545 |
| 2282 | 81.245 |
| 2280 | 80.38 |
| 2278 | 80.017 |
| 2276 | 80.039 |
| 2274 | 80.062 |
| 2272 | 80.036 |
| 2270 | 80.694 |
| 2268 | 81.067 |
| 2266 | 80.99 |
| 2264 | 81.268 |
| 2262 | 80.568 |
| 2260 | 80.816 |
| 2258 | 80.984 |
| 2256 | 81.538 |
| 2254 | 82.439 |
| 2252 | 81.99 |
| 2250 | 81.673 |
| 2248 | 81.454 |
| 2246 | 80.691 |
| 2244 | 80.836 |
| 2242 | 80.696 |
| 2240 | 80.518 |
| 2238 | 81.097 |
| 2236 | 80.959 |
| 2234 | 80.904 |
| 2232 | 80.61 |
| 2230 | 80.482 |
| 2228 | 80.8 |
| 2226 | 80.959 |
| 2224 | 81.299 |
| 2222 | 80.902 |
| 2220 | 80.299 |
| 2218 | 80.497 |
| 2216 | 79.983 |
| 2214 | 79.702 |
| 2212 | 80.005 |
| 2210 | 79.786 |
| 2208 | 80.596 |
| 2206 | 80.723 |
| 2204 | 80.616 |
| 2202 | 80.341 |
| 2200 | 80.223 |
| 2198 | 80.834 |
| 2196 | 81.144 |
| 2194 | 82.109 |
| 2192 | 81.72 |
| 2190 | 80.893 |
| 2188 | 80.774 |
| 2186 | 80.579 |
| 2184 | 81.017 |
| 2182 | 82.155 |
| 2180 | 82.335 |
| 2178 | 82.322 |
| 2176 | 82.599 |
| 2174 | 82.245 |
| 2172 | 82.119 |
| 2170 | 82.49 |
| 2168 | 82.267 |
| 2166 | 82.457 |
| 2164 | 82.607 |
| 2162 | 81.906 |
| 2160 | 82.313 |
| 2158 | 82.01 |
| 2156 | 82.121 |
| 2154 | 82.91 |
| 2152 | 82.846 |
| 2150 | 82.649 |
| 2148 | 82.747 |
| 2146 | 83.023 |
| 2144 | 82.933 |
| 2142 | 83.929 |
| 2140 | 83.838 |
| 2138 | 83.862 |
| 2136 | 84 |
| 2134 | 83.732 |
| 2132 | 84.022 |
| 2130 | 83.919 |
| 2128 | 83.846 |
| 2126 | 84.105 |
| 2124 | 84.094 |
| 2122 | 83.809 |
| 2120 | 84.034 |
| 2118 | 83.78 |
| 2116 | 83.693 |
| 2114 | 83.568 |
| 2112 | 82.827 |
| 2110 | 83.077 |
| 2108 | 83.255 |
| 2106 | 82.997 |
| 2104 | 83.535 |
| 2102 | 83.363 |
| 2100 | 82.884 |
| 2098 | 83.16 |
| 2096 | 83.051 |
| 2094 | 82.975 |
| 2092 | 83.092 |
| 2090 | 83.159 |
| 2088 | 83.362 |
| 2086 | 83.076 |
| 2084 | 82.936 |
| 2082 | 82.296 |
| 2080 | 82.146 |
| 2078 | 82.404 |
| 2076 | 82.439 |
| 2074 | 82.588 |
| 2072 | 81.959 |
| 2070 | 81.669 |
| 2068 | 81.592 |
| 2066 | 81.559 |
| 2064 | 81.824 |
| 2062 | 81.744 |
| 2060 | 81.652 |
| 2058 | 81.723 |
| 2056 | 81.894 |
| 2054 | 82.006 |
| 2052 | 81.829 |
| 2050 | 82.072 |
| 2048 | 81.956 |
| 2046 | 81.905 |
| 2044 | 81.728 |
| 2042 | 81.082 |
| 2040 | 81.15 |
| 2038 | 80.99 |
| 2036 | 81.123 |
| 2034 | 81.519 |
| 2032 | 81.177 |
| 2030 | 80.743 |
| 2028 | 80.789 |
| 2026 | 80.755 |
| 2024 | 80.626 |
| 2022 | 80.691 |
| 2020 | 80.486 |
| 2018 | 80.172 |
| 2016 | 80.002 |
| 2014 | 80.141 |
| 2012 | 80.103 |
| 2010 | 80.157 |
| 2008 | 80.326 |
| 2006 | 80.051 |
| 2004 | 80.013 |
| 2002 | 80.113 |
| 2000 | 79.85 |
| 1998 | 80.153 |
| 1996 | 79.859 |
| 1994 | 79.519 |
| 1992 | 79.422 |
| 1990 | 79.158 |
| 1988 | 79.293 |
| 1986 | 79.137 |
| 1984 | 79.298 |
| 1982 | 79.114 |
| 1980 | 78.903 |
| 1978 | 78.732 |
| 1976 | 78.396 |
| 1974 | 78.473 |
| 1972 | 78.782 |
| 1970 | 78.981 |
| 1968 | 78.998 |
| 1966 | 78.908 |
| 1964 | 78.755 |
| 1962 | 78.685 |
| 1960 | 78.514 |
| 1958 | 78.637 |
| 1956 | 78.559 |
| 1954 | 78.638 |
| 1952 | 79.166 |
| 1950 | 78.913 |
| 1948 | 78.693 |
| 1946 | 78.408 |
| 1944 | 77.808 |
| 1942 | 78.141 |
| 1940 | 78.364 |
| 1938 | 78.506 |
| 1936 | 78.876 |
| 1934 | 78.799 |
| 1932 | 78.76 |
| 1930 | 78.99 |
| 1928 | 78.946 |
| 1926 | 78.561 |
| 1924 | 78.939 |
| 1922 | 78.69 |
| 1920 | 79.154 |
| 1918 | 79.181 |
| 1916 | 78.637 |
| 1914 | 78.851 |
| 1912 | 78.413 |
| 1910 | 78.446 |
| 1908 | 78.967 |
| 1906 | 78.939 |
| 1904 | 78.896 |
| 1902 | 78.547 |
| 1900 | 78.75 |
| 1898 | 79.002 |
| 1896 | 79.295 |
| 1894 | 79.385 |
| 1892 | 79.621 |
| 1890 | 79.73 |
| 1888 | 79.659 |
| 1886 | 79.656 |
| 1884 | 79.607 |
| 1882 | 79.411 |
| 1880 | 79.625 |
| 1878 | 79.771 |
| 1876 | 79.974 |
| 1874 | 80.491 |
| 1872 | 80.338 |
| 1870 | 80.449 |
| 1868 | 80.271 |
| 1866 | 80.278 |
| 1864 | 80.596 |
| 1862 | 80.467 |
| 1860 | 80.671 |
| 1858 | 80.801 |
| 1856 | 80.855 |
| 1854 | 80.86 |
| 1852 | 80.875 |
| 1850 | 80.976 |
| 1848 | 80.893 |
| 1846 | 81.262 |
| 1844 | 81.501 |
| 1842 | 81.383 |
| 1840 | 81.704 |
| 1838 | 81.629 |
| 1836 | 81.39 |
| 1834 | 81.387 |
| 1832 | 81.327 |
| 1830 | 81.176 |
| 1828 | 81.17 |
| 1826 | 81.254 |
| 1824 | 81.106 |
| 1822 | 81.124 |
| 1820 | 81.166 |
| 1818 | 81.172 |
| 1816 | 81.234 |
| 1814 | 81.415 |
| 1812 | 81.45 |
| 1810 | 81.424 |
| 1808 | 81.388 |
| 1806 | 81.288 |
| 1804 | 81.222 |
| 1802 | 81.138 |
| 1800 | 81.144 |
| 1798 | 81.106 |
| 1796 | 81.083 |
| 1794 | 81.175 |
| 1792 | 81.018 |
| 1790 | 81.053 |
| 1788 | 81.208 |
| 1786 | 81.149 |
| 1784 | 81.401 |
| 1782 | 81.416 |
| 1780 | 81.343 |
| 1778 | 81.341 |
| 1776 | 81.156 |
| 1774 | 81.148 |
| 1772 | 81.134 |
| 1770 | 81.103 |
| 1768 | 81.206 |
| 1766 | 81.195 |
| 1764 | 81.21 |
| 1762 | 81.345 |
| 1760 | 81.398 |
| 1758 | 81.382 |
| 1756 | 81.505 |
| 1754 | 81.527 |
| 1752 | 81.575 |
| 1750 | 81.658 |
| 1748 | 81.609 |
| 1746 | 81.477 |
| 1744 | 81.48 |
| 1742 | 81.587 |
| 1740 | 81.641 |
| 1738 | 81.827 |
| 1736 | 81.842 |
| 1734 | 81.797 |
| 1732 | 81.769 |
| 1730 | 81.802 |
| 1728 | 81.853 |
| 1726 | 81.996 |
| 1724 | 82.151 |
| 1722 | 82.109 |
| 1720 | 82.099 |
| 1718 | 81.961 |
| 1716 | 81.884 |
| 1714 | 82.011 |
| 1712 | 82.082 |
| 1710 | 82.133 |
| 1708 | 82.175 |
| 1706 | 82.105 |
| 1704 | 82.136 |
| 1702 | 82.228 |
| 1700 | 82.252 |
| 1698 | 82.303 |
| 1696 | 82.264 |
| 1694 | 82.383 |
| 1692 | 82.385 |
| 1690 | 82.247 |
| 1688 | 82.312 |
| 1686 | 82.094 |
| 1684 | 82.129 |
| 1682 | 82.217 |
| 1680 | 82.05 |
| 1678 | 82.199 |
| 1676 | 82.114 |
| 1674 | 82.116 |
| 1672 | 82.185 |
| 1670 | 82.021 |
| 1668 | 82.05 |
| 1666 | 81.923 |
| 1664 | 81.92 |
| 1662 | 82.026 |
| 1660 | 82.011 |
| 1658 | 82.086 |
| 1656 | 82.042 |
| 1654 | 81.951 |
| 1652 | 81.967 |
| 1650 | 82.066 |
| 1648 | 82.098 |
| 1646 | 82.198 |
| 1644 | 82.12 |
| 1642 | 82.083 |
| 1640 | 82.017 |
| 1638 | 81.93 |
| 1636 | 81.959 |
| 1634 | 82.128 |
| 1632 | 82.253 |
| 1630 | 82.238 |
| 1628 | 82.298 |
| 1626 | 82.1 |
| 1624 | 82.089 |
| 1622 | 82.115 |
| 1620 | 82.198 |
| 1618 | 82.223 |
| 1616 | 82.246 |
| 1614 | 82.206 |
| 1612 | 82.072 |
| 1610 | 82.09 |
| 1608 | 82.085 |
| 1606 | 82.151 |
| 1604 | 82.193 |
| 1602 | 82.205 |
| 1600 | 82.16 |
| 1598 | 82.144 |
| 1596 | 82.122 |
| 1594 | 82.206 |
| 1592 | 82.237 |
| 1590 | 82.258 |
| 1588 | 82.316 |
| 1586 | 82.196 |
| 1584 | 82.169 |
| 1582 | 82.173 |
| 1580 | 82.161 |
| 1578 | 82.156 |
| 1576 | 82.274 |
| 1574 | 82.252 |
| 1572 | 82.319 |
| 1570 | 82.453 |
| 1568 | 82.428 |
| 1566 | 82.512 |
| 1564 | 82.485 |
| 1562 | 82.426 |
| 1560 | 82.383 |
| 1558 | 82.324 |
| 1556 | 82.335 |
| 1554 | 82.367 |
| 1552 | 82.248 |
| 1550 | 82.187 |
| 1548 | 82.092 |
| 1546 | 81.978 |
| 1544 | 82.09 |
| 1542 | 82.178 |
| 1540 | 82.168 |
| 1538 | 82.242 |
| 1536 | 82.286 |
| 1534 | 82.217 |
| 1532 | 82.304 |
| 1530 | 82.294 |
| 1528 | 82.291 |
| 1526 | 82.34 |
| 1524 | 82.363 |
| 1522 | 82.326 |
| 1520 | 82.286 |
| 1518 | 82.31 |
| 1516 | 82.296 |
| 1514 | 82.412 |
| 1512 | 82.525 |
| 1510 | 82.513 |
| 1508 | 82.466 |
| 1506 | 82.332 |
| 1504 | 82.22 |
| 1502 | 82.202 |
| 1500 | 82.244 |
| 1498 | 82.268 |
| 1496 | 82.231 |
| 1494 | 82.265 |
| 1492 | 82.255 |
| 1490 | 82.184 |
| 1488 | 82.234 |
| 1486 | 82.224 |
| 1484 | 82.226 |
| 1482 | 82.341 |
| 1480 | 82.297 |
| 1478 | 82.302 |
| 1476 | 82.244 |
| 1474 | 82.211 |
| 1472 | 82.251 |
| 1470 | 82.219 |
| 1468 | 82.178 |
| 1466 | 82.147 |
| 1464 | 82.115 |
| 1462 | 82.109 |
| 1460 | 82.208 |
| 1458 | 82.193 |
| 1456 | 82.197 |
| 1454 | 82.223 |
| 1452 | 82.175 |
| 1450 | 82.174 |
| 1448 | 82.17 |
| 1446 | 82.11 |
| 1444 | 82.073 |
| 1442 | 82.072 |
| 1440 | 82.102 |
| 1438 | 82.063 |
| 1436 | 82.159 |
| 1434 | 82.168 |
| 1432 | 82.142 |
| 1430 | 82.237 |
| 1428 | 82.178 |
| 1426 | 82.192 |
| 1424 | 82.239 |
| 1422 | 82.233 |
| 1420 | 82.248 |
| 1418 | 82.26 |
| 1416 | 82.256 |
| 1414 | 82.229 |
| 1412 | 82.284 |
| 1410 | 82.274 |
| 1408 | 82.321 |
| 1406 | 82.377 |
| 1404 | 82.296 |
| 1402 | 82.361 |
| 1400 | 82.319 |
| 1398 | 82.351 |
| 1396 | 82.44 |
| 1394 | 82.442 |
| 1392 | 82.474 |
| 1390 | 82.503 |
| 1388 | 82.533 |
| 1386 | 82.566 |
| 1384 | 82.646 |
| 1382 | 82.633 |
| 1380 | 82.652 |
| 1378 | 82.674 |
| 1376 | 82.623 |
| 1374 | 82.646 |
| 1372 | 82.629 |
| 1370 | 82.672 |
| 1368 | 82.735 |
| 1366 | 82.798 |
| 1364 | 82.788 |
| 1362 | 82.782 |
| 1360 | 82.842 |
| 1358 | 82.891 |
| 1356 | 82.983 |
| 1354 | 83.002 |
| 1352 | 82.969 |
| 1350 | 82.96 |
| 1348 | 82.949 |
| 1346 | 82.977 |
| 1344 | 82.992 |
| 1342 | 82.938 |
| 1340 | 82.94 |
| 1338 | 82.832 |
| 1336 | 82.832 |
| 1334 | 82.87 |
| 1332 | 82.901 |
| 1330 | 82.983 |
| 1328 | 82.989 |
| 1326 | 83.001 |
| 1324 | 82.979 |
| 1322 | 83.023 |
| 1320 | 82.997 |
| 1318 | 83.042 |
| 1316 | 83.056 |
| 1314 | 83.089 |
| 1312 | 83.102 |
| 1310 | 83.041 |
| 1308 | 83.102 |
| 1306 | 83.07 |
| 1304 | 83.116 |
| 1302 | 83.177 |
| 1300 | 83.126 |
| 1298 | 83.12 |
| 1296 | 83.092 |
| 1294 | 83.057 |
| 1292 | 83.059 |
| 1290 | 83.087 |
| 1288 | 83.115 |
| 1286 | 83.105 |
| 1284 | 83.121 |
| 1282 | 83.123 |
| 1280 | 83.14 |
| 1278 | 83.169 |
| 1276 | 83.203 |
| 1274 | 83.213 |
| 1272 | 83.176 |
| 1270 | 83.171 |
| 1268 | 83.159 |
| 1266 | 83.15 |
| 1264 | 83.173 |
| 1262 | 83.206 |
| 1260 | 83.23 |
| 1258 | 83.242 |
| 1256 | 83.29 |
| 1254 | 83.342 |
| 1252 | 83.339 |
| 1250 | 83.35 |
| 1248 | 83.327 |
| 1246 | 83.287 |
| 1244 | 83.254 |
| 1242 | 83.251 |
| 1240 | 83.247 |
| 1238 | 83.279 |
| 1236 | 83.295 |
| 1234 | 83.366 |
| 1232 | 83.404 |
| 1230 | 83.381 |
| 1228 | 83.428 |
| 1226 | 83.368 |
| 1224 | 83.367 |
| 1222 | 83.418 |
| 1220 | 83.389 |
| 1218 | 83.353 |
| 1216 | 83.386 |
| 1214 | 83.34 |
| 1212 | 83.374 |
| 1210 | 83.432 |
| 1208 | 83.445 |
| 1206 | 83.426 |
| 1204 | 83.418 |
| 1202 | 83.418 |
| 1200 | 83.386 |
| 1198 | 83.405 |
| 1196 | 83.421 |
| 1194 | 83.405 |
| 1192 | 83.395 |
| 1190 | 83.451 |
| 1188 | 83.545 |
| 1186 | 83.59 |
| 1184 | 83.594 |
| 1182 | 83.574 |
| 1180 | 83.514 |
| 1178 | 83.512 |
| 1176 | 83.607 |
| 1174 | 83.635 |
| 1172 | 83.623 |
| 1170 | 83.587 |
| 1168 | 83.503 |
| 1166 | 83.506 |
| 1164 | 83.45 |
| 1162 | 83.481 |
| 1160 | 83.53 |
| 1158 | 83.492 |
| 1156 | 83.525 |
| 1154 | 83.507 |
| 1152 | 83.451 |
| 1150 | 83.501 |
| 1148 | 83.505 |
| 1146 | 83.552 |
| 1144 | 83.581 |
| 1142 | 83.513 |
| 1140 | 83.492 |
| 1138 | 83.506 |
| 1136 | 83.559 |
| 1134 | 83.63 |
| 1132 | 83.618 |
| 1130 | 83.545 |
| 1128 | 83.501 |
| 1126 | 83.472 |
| 1124 | 83.528 |
| 1122 | 83.549 |
| 1120 | 83.553 |
| 1118 | 83.558 |
| 1116 | 83.577 |
| 1114 | 83.641 |
| 1112 | 83.595 |
| 1110 | 83.589 |
| 1108 | 83.579 |
| 1106 | 83.516 |
| 1104 | 83.545 |
| 1102 | 83.556 |
| 1100 | 83.536 |
| 1098 | 83.58 |
| 1096 | 83.614 |
| 1094 | 83.639 |
| 1092 | 83.686 |
| 1090 | 83.662 |
| 1088 | 83.667 |
| 1086 | 83.666 |
| 1084 | 83.638 |
| 1082 | 83.636 |
| 1080 | 83.626 |
| 1078 | 83.618 |
| 1076 | 83.635 |
| 1074 | 83.63 |
| 1072 | 83.624 |
| 1070 | 83.659 |
| 1068 | 83.669 |
| 1066 | 83.728 |
| 1064 | 83.724 |
| 1062 | 83.7 |
| 1060 | 83.696 |
| 1058 | 83.684 |
| 1056 | 83.756 |
| 1054 | 83.771 |
| 1052 | 83.769 |
| 1050 | 83.784 |
| 1048 | 83.752 |
| 1046 | 83.779 |
| 1044 | 83.779 |
| 1042 | 83.77 |
| 1040 | 83.771 |
| 1038 | 83.79 |
| 1036 | 83.798 |
| 1034 | 83.833 |
| 1032 | 83.855 |
| 1030 | 83.828 |
| 1028 | 83.874 |
| 1026 | 83.883 |
| 1024 | 83.898 |
| 1022 | 83.963 |
| 1020 | 83.951 |
| 1018 | 83.98 |
| 1016 | 84.005 |
| 1014 | 83.977 |
| 1012 | 84.024 |
| 1010 | 84.01 |
| 1008 | 84.027 |
| 1006 | 84.089 |
| 1004 | 84.109 |
| 1002 | 84.125 |
| 1000 | 84.114 |
| 998 | 84.075 |
| 996 | 84.035 |
| 994 | 84.057 |
| 992 | 84.087 |
| 990 | 84.095 |
| 988 | 84.126 |
| 986 | 84.142 |
| 984 | 84.126 |
| 982 | 84.219 |
| 980 | 84.29 |
| 978 | 84.335 |
| 976 | 84.443 |
| 974 | 84.426 |
| 972 | 84.441 |
| 970 | 84.469 |
| 968 | 84.485 |
| 966 | 84.543 |
| 964 | 84.567 |
| 962 | 84.541 |
| 960 | 84.493 |
| 958 | 84.526 |
| 956 | 84.545 |
| 954 | 84.594 |
| 952 | 84.726 |
| 950 | 84.706 |
| 948 | 84.766 |
| 946 | 84.846 |
| 944 | 84.807 |
| 942 | 84.798 |
| 940 | 84.718 |
| 938 | 84.711 |
| 936 | 84.724 |
| 934 | 84.813 |
| 932 | 84.874 |
| 930 | 84.905 |
| 928 | 84.968 |
| 926 | 84.982 |
| 924 | 85.035 |
| 922 | 85.124 |
| 920 | 85.166 |
| 918 | 85.231 |
| 916 | 85.244 |
| 914 | 85.179 |
| 912 | 85.186 |
| 910 | 85.152 |
| 908 | 85.102 |
| 906 | 85.176 |
| 904 | 85.199 |
| 902 | 85.293 |
| 900 | 85.437 |
| 898 | 85.387 |
| 896 | 85.325 |
| 894 | 85.267 |
| 892 | 85.324 |
| 890 | 85.345 |
| 888 | 85.491 |
| 886 | 85.432 |
| 884 | 85.333 |
| 882 | 85.391 |
| 880 | 85.38 |
| 878 | 85.576 |
| 876 | 85.658 |
| 874 | 85.726 |
| 872 | 85.843 |
| 870 | 85.822 |
| 868 | 85.906 |
| 866 | 85.891 |
| 864 | 85.866 |
| 862 | 85.946 |
| 860 | 85.958 |
| 858 | 86.01 |
| 856 | 86.044 |
| 854 | 86.211 |
| 852 | 86.185 |
| 850 | 86.214 |
| 848 | 86.131 |
| 846 | 86.226 |
| 844 | 86.01 |
| 842 | 85.927 |
| 840 | 86.132 |
| 838 | 86.102 |
| 836 | 86.372 |
| 834 | 86.546 |
| 832 | 86.509 |
| 830 | 86.488 |
| 828 | 86.382 |
| 826 | 86.399 |
| 824 | 86.389 |
| 822 | 86.497 |
| 820 | 86.643 |
| 818 | 86.581 |
| 816 | 86.528 |
| 814 | 86.602 |
| 812 | 86.579 |
| 810 | 86.743 |
| 808 | 86.9 |
| 806 | 86.9 |
| 804 | 86.807 |
| 802 | 86.852 |
| 800 | 86.895 |
| 798 | 87.01 |
| 796 | 87.034 |
| 794 | 87.091 |
| 792 | 87.07 |
| 790 | 86.923 |
| 788 | 86.944 |
| 786 | 86.87 |
| 784 | 86.862 |
| 782 | 86.949 |
| 780 | 87.1 |
| 778 | 87.13 |
| 776 | 87.145 |
| 774 | 87.162 |
| 772 | 87.086 |
| 770 | 87.123 |
| 768 | 87.212 |
| 766 | 87.244 |
| 764 | 87.222 |
| 762 | 87.136 |
| 760 | 87.178 |
| 758 | 87.11 |
| 756 | 87.236 |
| 754 | 87.268 |
| 752 | 87.266 |
| 750 | 87.243 |
| 748 | 87.236 |
| 746 | 87.447 |
| 744 | 87.609 |
| 742 | 87.496 |
| 740 | 87.491 |
| 738 | 87.541 |
| 736 | 87.472 |
| 734 | 87.541 |
| 732 | 87.461 |
| 730 | 87.464 |
| 728 | 87.481 |
| 726 | 87.468 |
| 724 | 87.473 |
| 722 | 87.445 |
| 720 | 87.56 |
| 718 | 87.62 |
| 716 | 87.668 |
| 714 | 87.727 |
| 712 | 87.732 |
| 710 | 87.786 |
| 708 | 87.735 |
| 706 | 87.8 |
| 704 | 87.779 |
| 702 | 87.722 |
| 700 | 87.839 |
| 698 | 87.785 |
| 696 | 87.896 |
| 694 | 88.039 |
| 692 | 88.108 |
| 690 | 88.367 |
| 688 | 88.316 |
| 686 | 88.263 |
| 684 | 88.241 |
| 682 | 88.099 |
| 680 | 88.121 |
| 678 | 88.181 |
| 676 | 88.193 |
| 674 | 88.289 |
| 672 | 88.384 |
| 670 | 88.371 |
| 668 | 88.475 |
| 666 | 88.552 |
| 664 | 88.514 |
| 662 | 88.561 |
| 660 | 88.606 |
| 658 | 88.541 |
| 656 | 88.662 |
| 654 | 88.693 |
| 652 | 88.697 |
| 650 | 88.769 |
| 648 | 88.766 |
| 646 | 88.798 |
| 644 | 88.819 |
| 642 | 88.856 |
| 640 | 88.902 |
| 638 | 89.059 |
| 636 | 89.156 |
| 634 | 89.195 |
| 632 | 89.192 |
| 630 | 89.155 |
| 628 | 89.167 |
| 626 | 89.152 |
| 624 | 89.161 |
| 622 | 89.264 |
| 620 | 89.313 |
| 618 | 89.352 |
| 616 | 89.418 |
| 614 | 89.431 |
| 612 | 89.461 |
| 610 | 89.55 |
| 608 | 89.467 |
| 606 | 89.401 |
| 604 | 89.532 |
| 602 | 89.558 |
| 600 | 89.717 |
| 598 | 89.81 |
| 596 | 89.806 |
| 594 | 89.796 |
| 592 | 89.901 |
| 590 | 90.027 |
| 588 | 90.047 |
| 586 | 90.204 |
| 584 | 90.199 |
| 582 | 90.249 |
| 580 | 90.238 |
| 578 | 90.231 |
| 576 | 90.313 |
| 574 | 90.304 |
| 572 | 90.379 |
| 570 | 90.433 |
| 568 | 90.476 |
| 566 | 90.586 |
| 564 | 90.681 |
| 562 | 90.823 |
| 560 | 90.983 |
| 558 | 91.009 |
| 556 | 91.038 |
| 554 | 91.051 |
| 552 | 91.189 |
| 550 | 91.375 |
| 548 | 91.468 |
| 546 | 91.611 |
| 544 | 91.601 |
| 542 | 91.513 |
| 540 | 91.666 |
| 538 | 91.755 |
| 536 | 91.878 |
| 534 | 92.037 |
| 532 | 92.037 |
| 530 | 92.055 |
| 528 | 92.121 |
| 526 | 92.262 |
| 524 | 92.486 |
| 522 | 92.748 |
| 520 | 92.841 |
| 518 | 92.815 |
| 516 | 92.879 |
| 514 | 92.962 |
| 512 | 93.047 |
| 510 | 93.217 |
| 508 | 93.246 |
| 506 | 93.371 |
| 504 | 93.512 |
| 502 | 93.644 |
| 500 | 93.856 |
| 498 | 94.045 |
| 496 | 94.146 |
| 494 | 94.21 |
| 492 | 94.326 |
| 490 | 94.391 |
| 488 | 94.602 |
| 486 | 94.675 |
| 484 | 94.867 |
| 482 | 95.003 |
| 480 | 95.016 |
| 478 | 95.239 |
| 476 | 95.349 |
| 474 | 95.436 |
| 472 | 95.651 |
| 470 | 95.78 |
| 468 | 95.841 |
| 466 | 96.008 |
| 464 | 96.222 |
| 462 | 96.319 |
| 460 | 96.437 |
| 458 | 96.61 |
| 456 | 96.77 |
| 454 | 96.945 |
| 452 | 97.075 |
| 450 | 97.074 |
| 448 | 97.113 |
| 446 | 97.223 |
| 444 | 97.432 |
| 442 | 97.534 |
| 440 | 97.723 |
| 438 | 97.958 |
| 436 | 97.989 |
| 434 | 98.102 |
| 432 | 98.09 |
| 430 | 98.176 |
| 428 | 98.44 |
| 426 | 98.701 |
| 424 | 98.62 |
| 422 | 98.461 |
| 420 | 98.956 |
| 418 | 98.89 |
| 416 | 98.797 |
| 414 | 98.67 |
| 412 | 98.627 |
| 410 | 98.686 |
| 408 | 98.44 |
| 406 | 98.275 |
| 404 | 97.619 |
| 402 | 96.919 |
| 400 | 95.955 |
| 398 | 94.914 |
| 396 | 94.006 |
| 394 | 92.833 |
| 392 | 91.884 |
| 390 | 90.827 |
| 388 | 89.916 |
| 386 | 89.031 |
| 384 | 88.118 |
| 382 | 87.441 |
| 380 | 86.76 |
| 378 | 86.266 |
| 376 | 85.696 |
| 374 | 84.327 |
| 372 | 83.909 |
| 370 | 83.23 |
| 368 | 83.015 |
| 366 | 82.9 |
| 364 | 82.432 |
| 362 | 82.194 |
| 360 | 81.764 |
| 358 | 81.497 |
| 356 | 81.156 |
| 354 | 80.498 |
| 352 | 79.697 |
| 350 | 78.612 |
| 348 | 77.896 |
| 346 | 76.994 |
| 344 | 75.264 |
| 342 | 73.187 |
| 340 | 72.978 |
| 338 | 72.373 |
| 336 | 70.936 |
| 334 | 69.138 |
| 332 | 67.305 |
| 330 | 65.49 |
| 328 | 63.633 |
| 326 | 61.79 |
| 324 | 60.021 |
| 322 | 58.519 |
| 320 | 57.041 |
| 318 | 55.754 |
| 316 | 54.672 |
| 314 | 53.11 |
| 312 | 51.576 |
| 310 | 49.899 |
| 308 | 48.068 |
| 306 | 46.162 |
| 304 | 44.421 |
| 302 | 42.596 |
| 300 | 41.048 |
| 298 | 39.698 |
| 296 | 38.38 |
| 294 | 37.132 |
| 292 | 36.05 |
| 290 | 35.227 |
| 288 | 34.533 |
| 286 | 34.068 |
| 284 | 33.324 |
| 282 | 32.714 |
| 280 | 31.805 |
| 278 | 30.894 |
| 276 | 30.297 |
| 274 | 29.533 |
| 272 | 29.41 |
| 270 | 29.356 |
| 268 | 29.397 |
| 266 | 29.554 |
| 264 | 29.576 |
| 262 | 29.827 |
| 260 | 29.964 |
| 258 | 30.099 |
| 256 | 30.318 |
| 254 | 30.675 |
| 252 | 31.183 |
| 250 | 31.61 |
| 248 | 32.163 |
| 246 | 32.552 |
| 244 | 33.391 |
| 242 | 34.231 |
| 240 | 35.365 |
| 238 | 36.475 |
| 236 | 37.168 |
| 234 | 38.308 |
| 232 | 39.69 |
| 230 | 41.333 |
| 228 | 42.838 |
| 226 | 45.014 |
| 224 | 45.42 |
| 222 | 45.986 |
| 220 | 45.804 |
| 218 | 44.999 |
| 216 | 45.03 |
| 214 | 45.135 |
| 212 | 46.084 |
| 210 | 46.125 |
| 208 | 45.873 |
| 206 | 47.293 |
| 204 | 46.209 |
| 202 | 55.308 |
| 200 | 62.519 |
| 198 | 60.408 |
| 196 | 67.056 |
| 194 | 101.32 |
| 192 | 100.487 |
| 190 | 71.132 |
