# Supplementary material for: Advancing energy storage and supercapacitor applications through the development of Li+-doped MgTiO3 perovskite nano-ceramics
Source: Sci Rep. 2024 Jan 22;14:1849. doi: 10.1038/s41598-024-52262-6 (PMC10803294; doi:10.1038/s41598-024-52262-6)
Supplement: Supplementary file 7 — Supplementary Information 7. [file 41598_2024_52262_MOESM7_ESM.docx]

Diffuse reflectance (Sample: MT5Li)

| TITLE | MT5Li |
| --- | --- |
| DATA TYPE | |
| ORIGIN | JASCO |
| OWNER |  |
| DATE | 23/05/22 |
| TIME | 10:28:38 |
| SPECTROMETER/DATA SYSTEM | JASCO Corp., V-570, Rev. 1.00 |
| RESOLUTION | |
| DELTAX | -2 |
| XUNITS | NANOMETERS |
| YUNITS | REFLECTANCE |
| FIRSTX | 2500 |
| LASTX | 190 |
| NPOINTS | 1156 |
| FIRSTY | 0.83649 |
| MAXY | 0.95132 |
| MINY | 0.29801 |
| XYDATA |  |
| 2500 | 83.649 |
| 2498 | 83.763 |
| 2496 | 84.056 |
| 2494 | 80.938 |
| 2492 | 81.135 |
| 2490 | 81.237 |
| 2488 | 83.208 |
| 2486 | 81.564 |
| 2484 | 81.083 |
| 2482 | 82.925 |
| 2480 | 81.678 |
| 2478 | 83.479 |
| 2476 | 85.475 |
| 2474 | 83.998 |
| 2472 | 84.794 |
| 2470 | 84.895 |
| 2468 | 82.246 |
| 2466 | 81.724 |
| 2464 | 81.151 |
| 2462 | 81.221 |
| 2460 | 82.075 |
| 2458 | 82.029 |
| 2456 | 83.158 |
| 2454 | 83.116 |
| 2452 | 83.05 |
| 2450 | 83.591 |
| 2448 | 81.334 |
| 2446 | 81.135 |
| 2444 | 81.181 |
| 2442 | 81.669 |
| 2440 | 82.38 |
| 2438 | 82.849 |
| 2436 | 83.458 |
| 2434 | 82.473 |
| 2432 | 83.176 |
| 2430 | 82.58 |
| 2428 | 83.479 |
| 2426 | 82.789 |
| 2424 | 81.538 |
| 2422 | 82.429 |
| 2420 | 80.528 |
| 2418 | 81.226 |
| 2416 | 82.427 |
| 2414 | 81.818 |
| 2412 | 82.596 |
| 2410 | 81.772 |
| 2408 | 81.831 |
| 2406 | 82.521 |
| 2404 | 82.792 |
| 2402 | 84.493 |
| 2400 | 84.395 |
| 2398 | 84.227 |
| 2396 | 83.707 |
| 2394 | 82.98 |
| 2392 | 82.788 |
| 2390 | 82.897 |
| 2388 | 83.516 |
| 2386 | 84.287 |
| 2384 | 84.174 |
| 2382 | 83.73 |
| 2380 | 83.74 |
| 2378 | 83.474 |
| 2376 | 83.695 |
| 2374 | 83.672 |
| 2372 | 84.276 |
| 2370 | 83.428 |
| 2368 | 83.064 |
| 2366 | 82.861 |
| 2364 | 82.093 |
| 2362 | 83.13 |
| 2360 | 83.511 |
| 2358 | 83.413 |
| 2356 | 84.131 |
| 2354 | 84.172 |
| 2352 | 84.917 |
| 2350 | 85.062 |
| 2348 | 84.585 |
| 2346 | 84.579 |
| 2344 | 84.753 |
| 2342 | 85.464 |
| 2340 | 85.247 |
| 2338 | 85.069 |
| 2336 | 83.135 |
| 2334 | 82.056 |
| 2332 | 82.691 |
| 2330 | 82.608 |
| 2328 | 83.411 |
| 2326 | 83.835 |
| 2324 | 83.3 |
| 2322 | 82.554 |
| 2320 | 82.97 |
| 2318 | 82.461 |
| 2316 | 82.08 |
| 2314 | 82.652 |
| 2312 | 82.693 |
| 2310 | 83.178 |
| 2308 | 83.395 |
| 2306 | 83.169 |
| 2304 | 82.902 |
| 2302 | 82.901 |
| 2300 | 82.521 |
| 2298 | 82.615 |
| 2296 | 82.592 |
| 2294 | 82.824 |
| 2292 | 82.564 |
| 2290 | 82.26 |
| 2288 | 82.416 |
| 2286 | 81.96 |
| 2284 | 82.648 |
| 2282 | 83.091 |
| 2280 | 82.913 |
| 2278 | 82.999 |
| 2276 | 82.524 |
| 2274 | 81.85 |
| 2272 | 81.802 |
| 2270 | 81.639 |
| 2268 | 81.38 |
| 2266 | 82.035 |
| 2264 | 81.888 |
| 2262 | 82.258 |
| 2260 | 82.904 |
| 2258 | 82.371 |
| 2256 | 82.065 |
| 2254 | 81.967 |
| 2252 | 81.957 |
| 2250 | 82.504 |
| 2248 | 82.993 |
| 2246 | 82.705 |
| 2244 | 82.338 |
| 2242 | 81.328 |
| 2240 | 80.716 |
| 2238 | 81.63 |
| 2236 | 81.634 |
| 2234 | 82.437 |
| 2232 | 82.38 |
| 2230 | 81.338 |
| 2228 | 81.374 |
| 2226 | 81.136 |
| 2224 | 81.305 |
| 2222 | 81.38 |
| 2220 | 81.085 |
| 2218 | 80.914 |
| 2216 | 80.756 |
| 2214 | 80.311 |
| 2212 | 80.439 |
| 2210 | 80.678 |
| 2208 | 81.212 |
| 2206 | 81.971 |
| 2204 | 81.93 |
| 2202 | 82.241 |
| 2200 | 82.311 |
| 2198 | 81.971 |
| 2196 | 82.343 |
| 2194 | 82.152 |
| 2192 | 81.944 |
| 2190 | 81.663 |
| 2188 | 81.627 |
| 2186 | 81.388 |
| 2184 | 81.166 |
| 2182 | 82.068 |
| 2180 | 82.479 |
| 2178 | 82.283 |
| 2176 | 82.928 |
| 2174 | 82.779 |
| 2172 | 82.36 |
| 2170 | 83.249 |
| 2168 | 82.992 |
| 2166 | 82.995 |
| 2164 | 83.39 |
| 2162 | 83.053 |
| 2160 | 83.765 |
| 2158 | 83.858 |
| 2156 | 83.8 |
| 2154 | 84.236 |
| 2152 | 83.931 |
| 2150 | 84.036 |
| 2148 | 84.148 |
| 2146 | 84.056 |
| 2144 | 84.006 |
| 2142 | 84.58 |
| 2140 | 84.575 |
| 2138 | 84.556 |
| 2136 | 84.994 |
| 2134 | 84.563 |
| 2132 | 84.239 |
| 2130 | 84.92 |
| 2128 | 84.751 |
| 2126 | 84.487 |
| 2124 | 84.946 |
| 2122 | 84.452 |
| 2120 | 84.397 |
| 2118 | 84.693 |
| 2116 | 84.843 |
| 2114 | 84.468 |
| 2112 | 84.039 |
| 2110 | 84.059 |
| 2108 | 83.765 |
| 2106 | 83.94 |
| 2104 | 84.278 |
| 2102 | 84.111 |
| 2100 | 84.114 |
| 2098 | 84.129 |
| 2096 | 83.818 |
| 2094 | 83.296 |
| 2092 | 83.172 |
| 2090 | 83.369 |
| 2088 | 83.664 |
| 2086 | 84.094 |
| 2084 | 83.923 |
| 2082 | 83.818 |
| 2080 | 83.507 |
| 2078 | 83.4 |
| 2076 | 83.444 |
| 2074 | 83.247 |
| 2072 | 83.152 |
| 2070 | 83.588 |
| 2068 | 83.597 |
| 2066 | 83.78 |
| 2064 | 83.905 |
| 2062 | 83.269 |
| 2060 | 83.128 |
| 2058 | 82.979 |
| 2056 | 83.088 |
| 2054 | 83.28 |
| 2052 | 83.73 |
| 2050 | 83.168 |
| 2048 | 82.856 |
| 2046 | 82.477 |
| 2044 | 82 |
| 2042 | 81.965 |
| 2040 | 81.889 |
| 2038 | 82.118 |
| 2036 | 82.024 |
| 2034 | 82.291 |
| 2032 | 82.406 |
| 2030 | 82.177 |
| 2028 | 82.262 |
| 2026 | 82.02 |
| 2024 | 82.065 |
| 2022 | 81.738 |
| 2020 | 81.753 |
| 2018 | 81.515 |
| 2016 | 81.368 |
| 2014 | 81.901 |
| 2012 | 81.609 |
| 2010 | 81.63 |
| 2008 | 81.231 |
| 2006 | 80.903 |
| 2004 | 80.658 |
| 2002 | 80.645 |
| 2000 | 80.921 |
| 1998 | 81.065 |
| 1996 | 81.123 |
| 1994 | 81.045 |
| 1992 | 81.04 |
| 1990 | 80.734 |
| 1988 | 80.791 |
| 1986 | 80.914 |
| 1984 | 80.504 |
| 1982 | 80.25 |
| 1980 | 79.964 |
| 1978 | 79.754 |
| 1976 | 79.88 |
| 1974 | 80.1 |
| 1972 | 80.238 |
| 1970 | 80.27 |
| 1968 | 80.021 |
| 1966 | 79.818 |
| 1964 | 79.763 |
| 1962 | 79.722 |
| 1960 | 79.266 |
| 1958 | 79.238 |
| 1956 | 79.178 |
| 1954 | 79.437 |
| 1952 | 79.944 |
| 1950 | 79.985 |
| 1948 | 79.984 |
| 1946 | 79.819 |
| 1944 | 79.913 |
| 1942 | 80.121 |
| 1940 | 80.148 |
| 1938 | 80.163 |
| 1936 | 80.299 |
| 1934 | 80.105 |
| 1932 | 80.178 |
| 1930 | 79.862 |
| 1928 | 79.51 |
| 1926 | 79.579 |
| 1924 | 79.609 |
| 1922 | 79.501 |
| 1920 | 79.867 |
| 1918 | 79.838 |
| 1916 | 79.506 |
| 1914 | 79.898 |
| 1912 | 79.766 |
| 1910 | 79.273 |
| 1908 | 80.031 |
| 1906 | 80.214 |
| 1904 | 81.17 |
| 1902 | 80.467 |
| 1900 | 80.205 |
| 1898 | 80.178 |
| 1896 | 80.354 |
| 1894 | 80.272 |
| 1892 | 80.681 |
| 1890 | 80.71 |
| 1888 | 80.681 |
| 1886 | 80.801 |
| 1884 | 80.704 |
| 1882 | 80.812 |
| 1880 | 81.133 |
| 1878 | 81.174 |
| 1876 | 81.316 |
| 1874 | 81.377 |
| 1872 | 81.608 |
| 1870 | 81.89 |
| 1868 | 81.789 |
| 1866 | 81.726 |
| 1864 | 81.554 |
| 1862 | 81.439 |
| 1860 | 81.645 |
| 1858 | 81.777 |
| 1856 | 81.994 |
| 1854 | 81.979 |
| 1852 | 82.075 |
| 1850 | 82.286 |
| 1848 | 82.191 |
| 1846 | 82.327 |
| 1844 | 82.322 |
| 1842 | 82.147 |
| 1840 | 82.165 |
| 1838 | 82.194 |
| 1836 | 81.896 |
| 1834 | 81.977 |
| 1832 | 82.122 |
| 1830 | 82.116 |
| 1828 | 82.258 |
| 1826 | 82.372 |
| 1824 | 82.347 |
| 1822 | 82.264 |
| 1820 | 82.369 |
| 1818 | 82.402 |
| 1816 | 82.353 |
| 1814 | 82.387 |
| 1812 | 82.363 |
| 1810 | 82.302 |
| 1808 | 82.34 |
| 1806 | 82.3 |
| 1804 | 82.383 |
| 1802 | 82.359 |
| 1800 | 82.278 |
| 1798 | 82.282 |
| 1796 | 82.127 |
| 1794 | 82.159 |
| 1792 | 82.111 |
| 1790 | 82.265 |
| 1788 | 82.27 |
| 1786 | 82.253 |
| 1784 | 82.325 |
| 1782 | 82.393 |
| 1780 | 82.56 |
| 1778 | 82.546 |
| 1776 | 82.473 |
| 1774 | 82.283 |
| 1772 | 82.184 |
| 1770 | 82.118 |
| 1768 | 82.165 |
| 1766 | 82.317 |
| 1764 | 82.27 |
| 1762 | 82.401 |
| 1760 | 82.382 |
| 1758 | 82.394 |
| 1756 | 82.459 |
| 1754 | 82.486 |
| 1752 | 82.669 |
| 1750 | 82.549 |
| 1748 | 82.632 |
| 1746 | 82.521 |
| 1744 | 82.478 |
| 1742 | 82.644 |
| 1740 | 82.609 |
| 1738 | 82.756 |
| 1736 | 82.788 |
| 1734 | 82.785 |
| 1732 | 82.918 |
| 1730 | 82.945 |
| 1728 | 83.078 |
| 1726 | 83.089 |
| 1724 | 83.144 |
| 1722 | 83.164 |
| 1720 | 83.077 |
| 1718 | 83.158 |
| 1716 | 83.088 |
| 1714 | 83.041 |
| 1712 | 83.144 |
| 1710 | 83.114 |
| 1708 | 82.995 |
| 1706 | 83.184 |
| 1704 | 83.232 |
| 1702 | 83.276 |
| 1700 | 83.545 |
| 1698 | 83.404 |
| 1696 | 83.327 |
| 1694 | 83.337 |
| 1692 | 83.386 |
| 1690 | 83.306 |
| 1688 | 83.356 |
| 1686 | 83.218 |
| 1684 | 83.014 |
| 1682 | 83.06 |
| 1680 | 82.924 |
| 1678 | 83.014 |
| 1676 | 83.076 |
| 1674 | 83.069 |
| 1672 | 83.119 |
| 1670 | 82.954 |
| 1668 | 82.97 |
| 1666 | 82.942 |
| 1664 | 82.906 |
| 1662 | 83.147 |
| 1660 | 83.075 |
| 1658 | 83.152 |
| 1656 | 83.124 |
| 1654 | 82.944 |
| 1652 | 82.889 |
| 1650 | 82.977 |
| 1648 | 82.953 |
| 1646 | 83.05 |
| 1644 | 83.121 |
| 1642 | 83 |
| 1640 | 82.951 |
| 1638 | 82.886 |
| 1636 | 82.894 |
| 1634 | 82.991 |
| 1632 | 83.129 |
| 1630 | 83.178 |
| 1628 | 83.146 |
| 1626 | 82.999 |
| 1624 | 82.989 |
| 1622 | 83.019 |
| 1620 | 83.125 |
| 1618 | 83.252 |
| 1616 | 83.258 |
| 1614 | 83.206 |
| 1612 | 83.117 |
| 1610 | 83.117 |
| 1608 | 83.083 |
| 1606 | 83.09 |
| 1604 | 83.118 |
| 1602 | 83.145 |
| 1600 | 83.194 |
| 1598 | 83.223 |
| 1596 | 83.207 |
| 1594 | 83.269 |
| 1592 | 83.289 |
| 1590 | 83.301 |
| 1588 | 83.266 |
| 1586 | 83.213 |
| 1584 | 83.185 |
| 1582 | 83.129 |
| 1580 | 83.236 |
| 1578 | 83.191 |
| 1576 | 83.228 |
| 1574 | 83.272 |
| 1572 | 83.249 |
| 1570 | 83.224 |
| 1568 | 83.211 |
| 1566 | 83.178 |
| 1564 | 83.097 |
| 1562 | 83.161 |
| 1560 | 83.141 |
| 1558 | 83.209 |
| 1556 | 83.221 |
| 1554 | 83.256 |
| 1552 | 83.214 |
| 1550 | 83.174 |
| 1548 | 83.225 |
| 1546 | 83.14 |
| 1544 | 83.222 |
| 1542 | 83.264 |
| 1540 | 83.203 |
| 1538 | 83.292 |
| 1536 | 83.212 |
| 1534 | 83.142 |
| 1532 | 83.14 |
| 1530 | 83.063 |
| 1528 | 83.119 |
| 1526 | 83.184 |
| 1524 | 83.267 |
| 1522 | 83.267 |
| 1520 | 83.254 |
| 1518 | 83.265 |
| 1516 | 83.194 |
| 1514 | 83.242 |
| 1512 | 83.319 |
| 1510 | 83.322 |
| 1508 | 83.315 |
| 1506 | 83.254 |
| 1504 | 83.227 |
| 1502 | 83.194 |
| 1500 | 83.291 |
| 1498 | 83.268 |
| 1496 | 83.19 |
| 1494 | 83.191 |
| 1492 | 83.137 |
| 1490 | 83.077 |
| 1488 | 83.062 |
| 1486 | 83.059 |
| 1484 | 83.038 |
| 1482 | 83.161 |
| 1480 | 83.135 |
| 1478 | 83.131 |
| 1476 | 83.104 |
| 1474 | 83.064 |
| 1472 | 83.088 |
| 1470 | 83.051 |
| 1468 | 82.982 |
| 1466 | 82.998 |
| 1464 | 83.019 |
| 1462 | 83.052 |
| 1460 | 83.182 |
| 1458 | 83.131 |
| 1456 | 83.132 |
| 1454 | 83.077 |
| 1452 | 83.081 |
| 1450 | 83.082 |
| 1448 | 83.027 |
| 1446 | 82.988 |
| 1444 | 82.91 |
| 1442 | 82.894 |
| 1440 | 82.94 |
| 1438 | 82.98 |
| 1436 | 83.044 |
| 1434 | 83.063 |
| 1432 | 83.058 |
| 1430 | 83.079 |
| 1428 | 83.071 |
| 1426 | 83.092 |
| 1424 | 83.117 |
| 1422 | 83.145 |
| 1420 | 83.083 |
| 1418 | 83.108 |
| 1416 | 83.067 |
| 1414 | 83.032 |
| 1412 | 83.063 |
| 1410 | 83.086 |
| 1408 | 83.081 |
| 1406 | 83.163 |
| 1404 | 83.209 |
| 1402 | 83.232 |
| 1400 | 83.284 |
| 1398 | 83.294 |
| 1396 | 83.333 |
| 1394 | 83.323 |
| 1392 | 83.366 |
| 1390 | 83.353 |
| 1388 | 83.313 |
| 1386 | 83.307 |
| 1384 | 83.362 |
| 1382 | 83.423 |
| 1380 | 83.437 |
| 1378 | 83.426 |
| 1376 | 83.314 |
| 1374 | 83.286 |
| 1372 | 83.324 |
| 1370 | 83.367 |
| 1368 | 83.403 |
| 1366 | 83.421 |
| 1364 | 83.432 |
| 1362 | 83.46 |
| 1360 | 83.522 |
| 1358 | 83.604 |
| 1356 | 83.663 |
| 1354 | 83.667 |
| 1352 | 83.67 |
| 1350 | 83.613 |
| 1348 | 83.6 |
| 1346 | 83.632 |
| 1344 | 83.653 |
| 1342 | 83.602 |
| 1340 | 83.524 |
| 1338 | 83.427 |
| 1336 | 83.402 |
| 1334 | 83.441 |
| 1332 | 83.513 |
| 1330 | 83.551 |
| 1328 | 83.526 |
| 1326 | 83.572 |
| 1324 | 83.549 |
| 1322 | 83.571 |
| 1320 | 83.586 |
| 1318 | 83.575 |
| 1316 | 83.576 |
| 1314 | 83.604 |
| 1312 | 83.585 |
| 1310 | 83.51 |
| 1308 | 83.54 |
| 1306 | 83.523 |
| 1304 | 83.582 |
| 1302 | 83.651 |
| 1300 | 83.66 |
| 1298 | 83.641 |
| 1296 | 83.633 |
| 1294 | 83.622 |
| 1292 | 83.603 |
| 1290 | 83.638 |
| 1288 | 83.65 |
| 1286 | 83.646 |
| 1284 | 83.656 |
| 1282 | 83.653 |
| 1280 | 83.653 |
| 1278 | 83.67 |
| 1276 | 83.674 |
| 1274 | 83.674 |
| 1272 | 83.653 |
| 1270 | 83.613 |
| 1268 | 83.62 |
| 1266 | 83.577 |
| 1264 | 83.585 |
| 1262 | 83.643 |
| 1260 | 83.596 |
| 1258 | 83.62 |
| 1256 | 83.651 |
| 1254 | 83.656 |
| 1252 | 83.699 |
| 1250 | 83.716 |
| 1248 | 83.723 |
| 1246 | 83.704 |
| 1244 | 83.67 |
| 1242 | 83.693 |
| 1240 | 83.674 |
| 1238 | 83.732 |
| 1236 | 83.758 |
| 1234 | 83.756 |
| 1232 | 83.829 |
| 1230 | 83.735 |
| 1228 | 83.791 |
| 1226 | 83.822 |
| 1224 | 83.743 |
| 1222 | 83.835 |
| 1220 | 83.775 |
| 1218 | 83.743 |
| 1216 | 83.834 |
| 1214 | 83.784 |
| 1212 | 83.805 |
| 1210 | 83.769 |
| 1208 | 83.744 |
| 1206 | 83.691 |
| 1204 | 83.648 |
| 1202 | 83.717 |
| 1200 | 83.709 |
| 1198 | 83.775 |
| 1196 | 83.771 |
| 1194 | 83.778 |
| 1192 | 83.806 |
| 1190 | 83.847 |
| 1188 | 83.903 |
| 1186 | 83.948 |
| 1184 | 83.91 |
| 1182 | 83.873 |
| 1180 | 83.832 |
| 1178 | 83.803 |
| 1176 | 83.871 |
| 1174 | 83.847 |
| 1172 | 83.844 |
| 1170 | 83.794 |
| 1168 | 83.736 |
| 1166 | 83.752 |
| 1164 | 83.744 |
| 1162 | 83.79 |
| 1160 | 83.841 |
| 1158 | 83.832 |
| 1156 | 83.806 |
| 1154 | 83.759 |
| 1152 | 83.666 |
| 1150 | 83.675 |
| 1148 | 83.694 |
| 1146 | 83.783 |
| 1144 | 83.848 |
| 1142 | 83.791 |
| 1140 | 83.782 |
| 1138 | 83.73 |
| 1136 | 83.717 |
| 1134 | 83.798 |
| 1132 | 83.746 |
| 1130 | 83.673 |
| 1128 | 83.66 |
| 1126 | 83.607 |
| 1124 | 83.666 |
| 1122 | 83.727 |
| 1120 | 83.726 |
| 1118 | 83.76 |
| 1116 | 83.77 |
| 1114 | 83.778 |
| 1112 | 83.736 |
| 1110 | 83.661 |
| 1108 | 83.613 |
| 1106 | 83.6 |
| 1104 | 83.64 |
| 1102 | 83.663 |
| 1100 | 83.721 |
| 1098 | 83.727 |
| 1096 | 83.73 |
| 1094 | 83.712 |
| 1092 | 83.693 |
| 1090 | 83.683 |
| 1088 | 83.69 |
| 1086 | 83.752 |
| 1084 | 83.712 |
| 1082 | 83.736 |
| 1080 | 83.739 |
| 1078 | 83.706 |
| 1076 | 83.726 |
| 1074 | 83.692 |
| 1072 | 83.681 |
| 1070 | 83.705 |
| 1068 | 83.684 |
| 1066 | 83.678 |
| 1064 | 83.668 |
| 1062 | 83.666 |
| 1060 | 83.725 |
| 1058 | 83.773 |
| 1056 | 83.795 |
| 1054 | 83.811 |
| 1052 | 83.76 |
| 1050 | 83.746 |
| 1048 | 83.731 |
| 1046 | 83.707 |
| 1044 | 83.762 |
| 1042 | 83.803 |
| 1040 | 83.841 |
| 1038 | 83.856 |
| 1036 | 83.805 |
| 1034 | 83.807 |
| 1032 | 83.853 |
| 1030 | 83.874 |
| 1028 | 83.913 |
| 1026 | 83.889 |
| 1024 | 83.835 |
| 1022 | 83.819 |
| 1020 | 83.851 |
| 1018 | 83.902 |
| 1016 | 83.945 |
| 1014 | 83.965 |
| 1012 | 84.008 |
| 1010 | 83.996 |
| 1008 | 84.003 |
| 1006 | 84.051 |
| 1004 | 84.041 |
| 1002 | 84.085 |
| 1000 | 84.088 |
| 998 | 84.1 |
| 996 | 84.086 |
| 994 | 84.084 |
| 992 | 84.114 |
| 990 | 84.111 |
| 988 | 84.153 |
| 986 | 84.192 |
| 984 | 84.202 |
| 982 | 84.257 |
| 980 | 84.283 |
| 978 | 84.295 |
| 976 | 84.312 |
| 974 | 84.301 |
| 972 | 84.33 |
| 970 | 84.389 |
| 968 | 84.472 |
| 966 | 84.573 |
| 964 | 84.628 |
| 962 | 84.66 |
| 960 | 84.673 |
| 958 | 84.66 |
| 956 | 84.683 |
| 954 | 84.686 |
| 952 | 84.749 |
| 950 | 84.771 |
| 948 | 84.829 |
| 946 | 84.82 |
| 944 | 84.776 |
| 942 | 84.797 |
| 940 | 84.787 |
| 938 | 84.864 |
| 936 | 84.955 |
| 934 | 85.005 |
| 932 | 85.03 |
| 930 | 85.052 |
| 928 | 85.078 |
| 926 | 85.099 |
| 924 | 85.133 |
| 922 | 85.19 |
| 920 | 85.196 |
| 918 | 85.222 |
| 916 | 85.157 |
| 914 | 85.145 |
| 912 | 85.215 |
| 910 | 85.239 |
| 908 | 85.333 |
| 906 | 85.424 |
| 904 | 85.419 |
| 902 | 85.567 |
| 900 | 85.707 |
| 898 | 85.739 |
| 896 | 85.727 |
| 894 | 85.684 |
| 892 | 85.74 |
| 890 | 85.808 |
| 888 | 85.887 |
| 886 | 85.895 |
| 884 | 85.739 |
| 882 | 85.731 |
| 880 | 85.763 |
| 878 | 85.92 |
| 876 | 86.023 |
| 874 | 85.983 |
| 872 | 86.061 |
| 870 | 85.859 |
| 868 | 85.987 |
| 866 | 86.083 |
| 864 | 86.08 |
| 862 | 86.413 |
| 860 | 86.474 |
| 858 | 86.511 |
| 856 | 86.454 |
| 854 | 86.506 |
| 852 | 86.56 |
| 850 | 86.714 |
| 848 | 86.815 |
| 846 | 86.828 |
| 844 | 86.594 |
| 842 | 86.494 |
| 840 | 86.659 |
| 838 | 86.792 |
| 836 | 87.028 |
| 834 | 87.272 |
| 832 | 87.262 |
| 830 | 87.204 |
| 828 | 87.164 |
| 826 | 87.073 |
| 824 | 87.111 |
| 822 | 87.204 |
| 820 | 87.292 |
| 818 | 87.294 |
| 816 | 87.193 |
| 814 | 87.207 |
| 812 | 87.194 |
| 810 | 87.277 |
| 808 | 87.363 |
| 806 | 87.403 |
| 804 | 87.297 |
| 802 | 87.355 |
| 800 | 87.384 |
| 798 | 87.406 |
| 796 | 87.52 |
| 794 | 87.62 |
| 792 | 87.626 |
| 790 | 87.569 |
| 788 | 87.591 |
| 786 | 87.46 |
| 784 | 87.403 |
| 782 | 87.492 |
| 780 | 87.529 |
| 778 | 87.665 |
| 776 | 87.631 |
| 774 | 87.644 |
| 772 | 87.672 |
| 770 | 87.709 |
| 768 | 87.75 |
| 766 | 87.692 |
| 764 | 87.802 |
| 762 | 87.688 |
| 760 | 87.69 |
| 758 | 87.721 |
| 756 | 87.653 |
| 754 | 87.644 |
| 752 | 87.748 |
| 750 | 87.781 |
| 748 | 87.782 |
| 746 | 87.88 |
| 744 | 87.993 |
| 742 | 87.91 |
| 740 | 87.761 |
| 738 | 87.863 |
| 736 | 87.873 |
| 734 | 88.009 |
| 732 | 88.006 |
| 730 | 87.97 |
| 728 | 88.037 |
| 726 | 88.045 |
| 724 | 87.923 |
| 722 | 87.904 |
| 720 | 87.975 |
| 718 | 87.912 |
| 716 | 87.996 |
| 714 | 88.055 |
| 712 | 88.037 |
| 710 | 88.098 |
| 708 | 88.007 |
| 706 | 88.069 |
| 704 | 88.034 |
| 702 | 87.993 |
| 700 | 88.087 |
| 698 | 88.108 |
| 696 | 88.123 |
| 694 | 88.162 |
| 692 | 88.143 |
| 690 | 88.315 |
| 688 | 88.286 |
| 686 | 88.275 |
| 684 | 88.345 |
| 682 | 88.133 |
| 680 | 88.107 |
| 678 | 88.098 |
| 676 | 88.109 |
| 674 | 88.203 |
| 672 | 88.34 |
| 670 | 88.385 |
| 668 | 88.473 |
| 666 | 88.541 |
| 664 | 88.423 |
| 662 | 88.429 |
| 660 | 88.416 |
| 658 | 88.379 |
| 656 | 88.495 |
| 654 | 88.611 |
| 652 | 88.61 |
| 650 | 88.611 |
| 648 | 88.637 |
| 646 | 88.567 |
| 644 | 88.569 |
| 642 | 88.63 |
| 640 | 88.587 |
| 638 | 88.688 |
| 636 | 88.744 |
| 634 | 88.738 |
| 632 | 88.813 |
| 630 | 88.821 |
| 628 | 88.831 |
| 626 | 88.796 |
| 624 | 88.715 |
| 622 | 88.797 |
| 620 | 88.743 |
| 618 | 88.736 |
| 616 | 88.848 |
| 614 | 88.804 |
| 612 | 88.814 |
| 610 | 88.953 |
| 608 | 88.891 |
| 606 | 88.795 |
| 604 | 88.853 |
| 602 | 88.832 |
| 600 | 88.857 |
| 598 | 88.894 |
| 596 | 88.941 |
| 594 | 88.877 |
| 592 | 88.942 |
| 590 | 89.041 |
| 588 | 89.048 |
| 586 | 89.146 |
| 584 | 89.206 |
| 582 | 89.157 |
| 580 | 89.108 |
| 578 | 89.143 |
| 576 | 89.163 |
| 574 | 89.126 |
| 572 | 89.173 |
| 570 | 89.247 |
| 568 | 89.259 |
| 566 | 89.343 |
| 564 | 89.483 |
| 562 | 89.539 |
| 560 | 89.57 |
| 558 | 89.655 |
| 556 | 89.695 |
| 554 | 89.696 |
| 552 | 89.801 |
| 550 | 89.885 |
| 548 | 89.961 |
| 546 | 90.034 |
| 544 | 90.063 |
| 542 | 90.043 |
| 540 | 90.128 |
| 538 | 90.281 |
| 536 | 90.408 |
| 534 | 90.513 |
| 532 | 90.536 |
| 530 | 90.575 |
| 528 | 90.59 |
| 526 | 90.774 |
| 524 | 90.885 |
| 522 | 91.098 |
| 520 | 91.193 |
| 518 | 91.244 |
| 516 | 91.343 |
| 514 | 91.459 |
| 512 | 91.546 |
| 510 | 91.699 |
| 508 | 91.679 |
| 506 | 91.807 |
| 504 | 91.941 |
| 502 | 92.089 |
| 500 | 92.274 |
| 498 | 92.443 |
| 496 | 92.451 |
| 494 | 92.437 |
| 492 | 92.58 |
| 490 | 92.578 |
| 488 | 92.877 |
| 486 | 93.025 |
| 484 | 93.095 |
| 482 | 93.299 |
| 480 | 93.326 |
| 478 | 93.347 |
| 476 | 93.403 |
| 474 | 93.483 |
| 472 | 93.634 |
| 470 | 93.791 |
| 468 | 93.916 |
| 466 | 93.924 |
| 464 | 94.006 |
| 462 | 93.985 |
| 460 | 94.021 |
| 458 | 94.212 |
| 456 | 94.271 |
| 454 | 94.432 |
| 452 | 94.58 |
| 450 | 94.421 |
| 448 | 94.398 |
| 446 | 94.455 |
| 444 | 94.454 |
| 442 | 94.663 |
| 440 | 94.773 |
| 438 | 94.896 |
| 436 | 94.988 |
| 434 | 95.069 |
| 432 | 95.058 |
| 430 | 95.08 |
| 428 | 95.132 |
| 426 | 95.125 |
| 424 | 94.675 |
| 422 | 94.378 |
| 420 | 94.228 |
| 418 | 93.967 |
| 416 | 93.486 |
| 414 | 92.954 |
| 412 | 92.22 |
| 410 | 91.314 |
| 408 | 90.421 |
| 406 | 89.21 |
| 404 | 87.498 |
| 402 | 85.871 |
| 400 | 84.134 |
| 398 | 82.515 |
| 396 | 81.165 |
| 394 | 79.992 |
| 392 | 78.964 |
| 390 | 78.197 |
| 388 | 77.563 |
| 386 | 76.814 |
| 384 | 76.334 |
| 382 | 75.656 |
| 380 | 75.11 |
| 378 | 74.736 |
| 376 | 74.187 |
| 374 | 73.443 |
| 372 | 73.286 |
| 370 | 72.785 |
| 368 | 72.623 |
| 366 | 72.38 |
| 364 | 71.919 |
| 362 | 71.674 |
| 360 | 71.022 |
| 358 | 70.623 |
| 356 | 70.139 |
| 354 | 69.602 |
| 352 | 69.02 |
| 350 | 68.389 |
| 348 | 67.438 |
| 346 | 66.279 |
| 344 | 63.948 |
| 342 | 62.15 |
| 340 | 61.719 |
| 338 | 61.407 |
| 336 | 60.179 |
| 334 | 59.03 |
| 332 | 57.746 |
| 330 | 56.502 |
| 328 | 55.189 |
| 326 | 53.888 |
| 324 | 52.878 |
| 322 | 51.891 |
| 320 | 50.978 |
| 318 | 49.982 |
| 316 | 49.172 |
| 314 | 48.133 |
| 312 | 47.147 |
| 310 | 46.306 |
| 308 | 44.94 |
| 306 | 43.669 |
| 304 | 42.428 |
| 302 | 40.88 |
| 300 | 40.008 |
| 298 | 38.902 |
| 296 | 37.963 |
| 294 | 37.187 |
| 292 | 35.965 |
| 290 | 35.318 |
| 288 | 34.776 |
| 286 | 34.287 |
| 284 | 33.877 |
| 282 | 33.493 |
| 280 | 32.508 |
| 278 | 31.832 |
| 276 | 31.476 |
| 274 | 30.512 |
| 272 | 30.275 |
| 270 | 30.049 |
| 268 | 29.801 |
| 266 | 30.303 |
| 264 | 30.678 |
| 262 | 30.949 |
| 260 | 31.234 |
| 258 | 31.502 |
| 256 | 31.899 |
| 254 | 32.477 |
| 252 | 32.887 |
| 250 | 33.26 |
| 248 | 33.745 |
| 246 | 34.512 |
| 244 | 35.311 |
| 242 | 36.091 |
| 240 | 37.011 |
| 238 | 37.834 |
| 236 | 38.958 |
| 234 | 40.378 |
| 232 | 42.023 |
| 230 | 43.381 |
| 228 | 44.713 |
| 226 | 46.486 |
| 224 | 47.017 |
| 222 | 47.729 |
| 220 | 48.208 |
| 218 | 47.789 |
| 216 | 47.806 |
| 214 | 47.511 |
| 212 | 48.506 |
| 210 | 47.809 |
| 208 | 45.24 |
| 206 | 45.61 |
| 204 | 46.716 |
| 202 | 49.791 |
| 200 | 55.703 |
| 198 | 61.736 |
| 196 | 68.263 |
| 194 | 89.816 |
| 192 | 70.887 |
| 190 | 40.187 |
