# Supplementary material for: Advancing energy storage and supercapacitor applications through the development of Li+-doped MgTiO3 perovskite nano-ceramics
Source: Sci Rep. 2024 Jan 22;14:1849. doi: 10.1038/s41598-024-52262-6 (PMC10803294; doi:10.1038/s41598-024-52262-6)
Supplement: Supplementary file 8 — Supplementary Information 8. [file 41598_2024_52262_MOESM8_ESM.docx]

XRD-MT5Li

'Id: "" Comment: "" Operator: "Lab Manager" Anode: "Cu" Scantype: "Coupled TwoTheta/Theta" TimePerStep: "54.3"

| 2theta | Intensity |
| --- | --- |
| 5 | 42.339 |
| 5.0499 | 40.884 |
| 5.0999 | 41.473 |
| 5.1498 | 40.258 |
| 5.1998 | 38.692 |
| 5.2497 | 39.834 |
| 5.2996 | 39.3 |
| 5.3496 | 38.214 |
| 5.3995 | 38.103 |
| 5.4495 | 36.206 |
| 5.4994 | 35.525 |
| 5.5494 | 36.446 |
| 5.5993 | 35.912 |
| 5.6492 | 34.788 |
| 5.6992 | 36.262 |
| 5.7491 | 33.683 |
| 5.7991 | 33.168 |
| 5.849 | 33.591 |
| 5.8989 | 32.007 |
| 5.9489 | 32.155 |
| 5.9988 | 31.326 |
| 6.0488 | 30.737 |
| 6.0987 | 31.694 |
| 6.1486 | 29.171 |
| 6.1986 | 29.908 |
| 6.2485 | 30.074 |
| 6.2985 | 28.564 |
| 6.3484 | 27.44 |
| 6.3984 | 29.337 |
| 6.4483 | 27.79 |
| 6.4982 | 26.298 |
| 6.5482 | 26.943 |
| 6.5981 | 26.446 |
| 6.6481 | 24.788 |
| 6.698 | 25.341 |
| 6.7479 | 24.162 |
| 6.7979 | 25.285 |
| 6.8478 | 24.899 |
| 6.8978 | 23.904 |
| 6.9477 | 23.628 |
| 6.9976 | 23.702 |
| 7.0476 | 22.505 |
| 7.0975 | 22.578 |
| 7.1475 | 22.099 |
| 7.1974 | 20.663 |
| 7.2474 | 20.405 |
| 7.2973 | 21.4 |
| 7.3472 | 22.21 |
| 7.3972 | 20.11 |
| 7.4471 | 19.521 |
| 7.4971 | 20.534 |
| 7.547 | 20.221 |
| 7.5969 | 18.692 |
| 7.6469 | 17.974 |
| 7.6968 | 18.766 |
| 7.7468 | 19.134 |
| 7.7967 | 17.956 |
| 7.8466 | 18.306 |
| 7.8966 | 17.127 |
| 7.9465 | 18.766 |
| 7.9965 | 15.985 |
| 8.0464 | 17.219 |
| 8.0964 | 17.919 |
| 8.1463 | 16.703 |
| 8.1962 | 16.446 |
| 8.2462 | 16.943 |
| 8.2961 | 16.943 |
| 8.3461 | 16.796 |
| 8.396 | 16.685 |
| 8.4459 | 15.157 |
| 8.4959 | 15.506 |
| 8.5458 | 16.648 |
| 8.5958 | 15.157 |
| 8.6457 | 15.727 |
| 8.6956 | 15.709 |
| 8.7456 | 15.175 |
| 8.7955 | 15.304 |
| 8.8455 | 15.046 |
| 8.8954 | 16.225 |
| 8.9454 | 13.849 |
| 8.9953 | 15.672 |
| 9.0452 | 15.138 |
| 9.0952 | 15.212 |
| 9.1451 | 15.341 |
| 9.1951 | 15.212 |
| 9.245 | 14.622 |
| 9.2949 | 14.309 |
| 9.3449 | 14.586 |
| 9.3948 | 14.659 |
| 9.4448 | 13.904 |
| 9.4947 | 14.567 |
| 9.5446 | 13.996 |
| 9.5946 | 13.278 |
| 9.6445 | 14.494 |
| 9.6945 | 13.812 |
| 9.7444 | 14.494 |
| 9.7944 | 14.549 |
| 9.8443 | 12.707 |
| 9.8942 | 14.033 |
| 9.9442 | 13.407 |
| 9.9941 | 14.18 |
| 10.0441 | 13.831 |
| 10.094 | 14.033 |
| 10.1439 | 13.223 |
| 10.1939 | 13.867 |
| 10.2438 | 14.788 |
| 10.2938 | 13.481 |
| 10.3437 | 12.505 |
| 10.3936 | 13.149 |
| 10.4436 | 12.523 |
| 10.4935 | 13.186 |
| 10.5435 | 13.536 |
| 10.5934 | 12.615 |
| 10.6434 | 13.794 |
| 10.6933 | 12.394 |
| 10.7432 | 12.689 |
| 10.7932 | 14.144 |
| 10.8431 | 13.241 |
| 10.8931 | 13.444 |
| 10.943 | 12.376 |
| 10.9929 | 12.707 |
| 11.0429 | 12.413 |
| 11.0928 | 12.597 |
| 11.1428 | 12.394 |
| 11.1927 | 12.155 |
| 11.2426 | 12.505 |
| 11.2926 | 11.657 |
| 11.3425 | 11.842 |
| 11.3925 | 12.431 |
| 11.4424 | 12.302 |
| 11.4924 | 12.155 |
| 11.5423 | 12.118 |
| 11.5922 | 11.971 |
| 11.6422 | 11.51 |
| 11.6921 | 11.915 |
| 11.7421 | 12.026 |
| 11.792 | 11.565 |
| 11.8419 | 11.51 |
| 11.8919 | 12.192 |
| 11.9418 | 11.123 |
| 11.9918 | 11.436 |
| 12.0417 | 10.295 |
| 12.0916 | 9.742 |
| 12.1416 | 10.847 |
| 12.1915 | 10.976 |
| 12.2415 | 9.963 |
| 12.2914 | 10.092 |
| 12.3413 | 9.908 |
| 12.3913 | 9.926 |
| 12.4412 | 9.116 |
| 12.4912 | 9.761 |
| 12.5411 | 9.245 |
| 12.5911 | 10.055 |
| 12.641 | 9.816 |
| 12.6909 | 9.245 |
| 12.7409 | 8.803 |
| 12.7908 | 8.269 |
| 12.8408 | 8.6 |
| 12.8907 | 9.042 |
| 12.9406 | 7.919 |
| 12.9906 | 8.582 |
| 13.0405 | 8.195 |
| 13.0905 | 7.937 |
| 13.1404 | 7.956 |
| 13.1903 | 8.545 |
| 13.2403 | 7.698 |
| 13.2902 | 7.403 |
| 13.3402 | 7.698 |
| 13.3901 | 7.017 |
| 13.4401 | 7.035 |
| 13.49 | 6.943 |
| 13.5399 | 7.495 |
| 13.5899 | 7.477 |
| 13.6398 | 6.722 |
| 13.6898 | 7.348 |
| 13.7397 | 7.072 |
| 13.7896 | 7.33 |
| 13.8396 | 6.519 |
| 13.8895 | 6.317 |
| 13.9395 | 6.427 |
| 13.9894 | 6.538 |
| 14.0393 | 6.796 |
| 14.0893 | 6.446 |
| 14.1392 | 5.912 |
| 14.1892 | 6.28 |
| 14.2391 | 6.593 |
| 14.2891 | 6.409 |
| 14.339 | 6.206 |
| 14.3889 | 6.28 |
| 14.4389 | 6.004 |
| 14.4888 | 6.114 |
| 14.5388 | 6.372 |
| 14.5887 | 6.022 |
| 14.6386 | 5.764 |
| 14.6886 | 5.691 |
| 14.7385 | 5.672 |
| 14.7885 | 5.654 |
| 14.8384 | 5.451 |
| 14.8883 | 5.617 |
| 14.9383 | 5.727 |
| 14.9882 | 5.359 |
| 15.0382 | 5.157 |
| 15.0881 | 5.341 |
| 15.1381 | 4.917 |
| 15.188 | 5.764 |
| 15.2379 | 5.82 |
| 15.2879 | 4.917 |
| 15.3378 | 5.249 |
| 15.3878 | 5.691 |
| 15.4377 | 5.506 |
| 15.4876 | 5.046 |
| 15.5376 | 5.157 |
| 15.5875 | 4.972 |
| 15.6375 | 5.304 |
| 15.6874 | 4.549 |
| 15.7373 | 4.788 |
| 15.7873 | 4.77 |
| 15.8372 | 5.801 |
| 15.8872 | 4.586 |
| 15.9371 | 5.175 |
| 15.9871 | 5.175 |
| 16.037 | 4.843 |
| 16.0869 | 4.899 |
| 16.1369 | 4.715 |
| 16.1868 | 4.365 |
| 16.2368 | 4.512 |
| 16.2867 | 4.733 |
| 16.3366 | 4.217 |
| 16.3866 | 4.254 |
| 16.4365 | 4.696 |
| 16.4865 | 4.457 |
| 16.5364 | 4.659 |
| 16.5863 | 4.862 |
| 16.6363 | 4.309 |
| 16.6862 | 4.843 |
| 16.7362 | 4.825 |
| 16.7861 | 4.586 |
| 16.8361 | 4.144 |
| 16.886 | 4.18 |
| 16.9359 | 3.867 |
| 16.9859 | 4.162 |
| 17.0358 | 4.567 |
| 17.0858 | 4.291 |
| 17.1357 | 4.18 |
| 17.1856 | 3.941 |
| 17.2356 | 4.291 |
| 17.2855 | 5.064 |
| 17.3355 | 4.567 |
| 17.3854 | 6.63 |
| 17.4353 | 8.103 |
| 17.4853 | 5.378 |
| 17.5352 | 4.457 |
| 17.5852 | 3.978 |
| 17.6351 | 4.107 |
| 17.6851 | 4.107 |
| 17.735 | 3.886 |
| 17.7849 | 4.751 |
| 17.8349 | 4.954 |
| 17.8848 | 5.783 |
| 17.9348 | 6.869 |
| 17.9847 | 6.519 |
| 18.0346 | 4.217 |
| 18.0846 | 3.591 |
| 18.1345 | 3.941 |
| 18.1845 | 3.665 |
| 18.2344 | 3.923 |
| 18.2843 | 3.628 |
| 18.3343 | 3.573 |
| 18.3842 | 4.107 |
| 18.4342 | 4.015 |
| 18.4841 | 3.683 |
| 18.5341 | 3.425 |
| 18.584 | 3.941 |
| 18.6339 | 3.499 |
| 18.6839 | 5.359 |
| 18.7338 | 7.109 |
| 18.7838 | 13.941 |
| 18.8337 | 17.735 |
| 18.8836 | 11.381 |
| 18.9336 | 5.322 |
| 18.9835 | 3.923 |
| 19.0335 | 3.61 |
| 19.0834 | 3.738 |
| 19.1333 | 3.794 |
| 19.1833 | 3.683 |
| 19.2332 | 3.241 |
| 19.2832 | 3.554 |
| 19.3331 | 3.039 |
| 19.3831 | 3.223 |
| 19.433 | 2.965 |
| 19.4829 | 3.665 |
| 19.5329 | 3.278 |
| 19.5828 | 3.057 |
| 19.6328 | 3.204 |
| 19.6827 | 2.947 |
| 19.7326 | 3.407 |
| 19.7826 | 3.057 |
| 19.8325 | 2.818 |
| 19.8825 | 3.444 |
| 19.9324 | 3.904 |
| 19.9823 | 3.941 |
| 20.0323 | 3.831 |
| 20.0822 | 3.186 |
| 20.1322 | 2.855 |
| 20.1821 | 3.37 |
| 20.2321 | 3.297 |
| 20.282 | 3.186 |
| 20.3319 | 2.652 |
| 20.3819 | 3.149 |
| 20.4318 | 3.352 |
| 20.4818 | 3.315 |
| 20.5317 | 2.67 |
| 20.5816 | 3.333 |
| 20.6316 | 2.836 |
| 20.6815 | 3.094 |
| 20.7315 | 3.481 |
| 20.7814 | 3.812 |
| 20.8313 | 4.512 |
| 20.8813 | 8.987 |
| 20.9312 | 18.379 |
| 20.9812 | 15.599 |
| 21.0311 | 8.14 |
| 21.0811 | 3.794 |
| 21.131 | 3.407 |
| 21.1809 | 2.689 |
| 21.2309 | 3.241 |
| 21.2808 | 3.002 |
| 21.3308 | 3.094 |
| 21.3807 | 2.173 |
| 21.4306 | 2.799 |
| 21.4806 | 2.541 |
| 21.5305 | 2.247 |
| 21.5805 | 2.449 |
| 21.6304 | 2.707 |
| 21.6803 | 2.302 |
| 21.7303 | 2.818 |
| 21.7802 | 2.394 |
| 21.8302 | 2.91 |
| 21.8801 | 2.983 |
| 21.9301 | 2.67 |
| 21.98 | 2.634 |
| 22.0299 | 2.376 |
| 22.0799 | 2.376 |
| 22.1298 | 2.099 |
| 22.1798 | 2.689 |
| 22.2297 | 2.486 |
| 22.2796 | 2.578 |
| 22.3296 | 2.431 |
| 22.3795 | 2.486 |
| 22.4295 | 2.21 |
| 22.4794 | 2.449 |
| 22.5293 | 2.413 |
| 22.5793 | 2.284 |
| 22.6292 | 1.989 |
| 22.6792 | 2.173 |
| 22.7291 | 2.56 |
| 22.7791 | 2.228 |
| 22.829 | 2.523 |
| 22.8789 | 2.597 |
| 22.9289 | 3.223 |
| 22.9788 | 2.652 |
| 23.0288 | 2.67 |
| 23.0787 | 2.762 |
| 23.1286 | 2.449 |
| 23.1786 | 2.597 |
| 23.2285 | 2.652 |
| 23.2785 | 1.989 |
| 23.3284 | 2.357 |
| 23.3783 | 3.002 |
| 23.4283 | 3.186 |
| 23.4782 | 3.131 |
| 23.5282 | 3.646 |
| 23.5781 | 5.985 |
| 23.6281 | 13.499 |
| 23.678 | 25.267 |
| 23.7279 | 18.987 |
| 23.7779 | 8.969 |
| 23.8278 | 3.941 |
| 23.8778 | 2.578 |
| 23.9277 | 2.21 |
| 23.9776 | 2.836 |
| 24.0276 | 2.468 |
| 24.0775 | 2.155 |
| 24.1275 | 2.155 |
| 24.1774 | 2.339 |
| 24.2273 | 1.971 |
| 24.2773 | 1.878 |
| 24.3272 | 2.376 |
| 24.3772 | 2.136 |
| 24.4271 | 1.897 |
| 24.4771 | 2.173 |
| 24.527 | 1.952 |
| 24.5769 | 2.099 |
| 24.6269 | 1.86 |
| 24.6768 | 2.32 |
| 24.7268 | 2.339 |
| 24.7767 | 2.136 |
| 24.8266 | 1.694 |
| 24.8766 | 1.952 |
| 24.9265 | 2.21 |
| 24.9765 | 2.118 |
| 25.0264 | 1.952 |
| 25.0763 | 3.112 |
| 25.1263 | 5.985 |
| 25.1762 | 11.897 |
| 25.2262 | 12.376 |
| 25.2761 | 7.974 |
| 25.3261 | 3.812 |
| 25.376 | 2.523 |
| 25.4259 | 1.584 |
| 25.4759 | 1.86 |
| 25.5258 | 1.805 |
| 25.5758 | 2.192 |
| 25.6257 | 1.786 |
| 25.6756 | 2.21 |
| 25.7256 | 1.934 |
| 25.7755 | 1.75 |
| 25.8255 | 1.823 |
| 25.8754 | 1.676 |
| 25.9253 | 1.639 |
| 25.9753 | 1.639 |
| 26.0252 | 1.584 |
| 26.0752 | 1.731 |
| 26.1251 | 1.602 |
| 26.1751 | 1.934 |
| 26.225 | 2.081 |
| 26.2749 | 2.118 |
| 26.3249 | 1.915 |
| 26.3748 | 1.731 |
| 26.4248 | 1.51 |
| 26.4747 | 1.731 |
| 26.5246 | 1.455 |
| 26.5746 | 1.381 |
| 26.6245 | 1.86 |
| 26.6745 | 1.565 |
| 26.7244 | 1.989 |
| 26.7743 | 1.86 |
| 26.8243 | 1.897 |
| 26.8742 | 1.805 |
| 26.9242 | 1.86 |
| 26.9741 | 2.044 |
| 27.024 | 2.505 |
| 27.074 | 4.512 |
| 27.1239 | 5.267 |
| 27.1739 | 3.702 |
| 27.2238 | 2.67 |
| 27.2738 | 1.915 |
| 27.3237 | 1.529 |
| 27.3736 | 1.86 |
| 27.4236 | 1.657 |
| 27.4735 | 1.381 |
| 27.5235 | 1.436 |
| 27.5734 | 1.473 |
| 27.6233 | 1.731 |
| 27.6733 | 1.436 |
| 27.7232 | 1.934 |
| 27.7732 | 1.455 |
| 27.8231 | 1.657 |
| 27.873 | 1.565 |
| 27.923 | 1.473 |
| 27.9729 | 1.4 |
| 28.0229 | 1.529 |
| 28.0728 | 1.455 |
| 28.1228 | 1.492 |
| 28.1727 | 1.418 |
| 28.2226 | 1.455 |
| 28.2726 | 1.492 |
| 28.3225 | 1.363 |
| 28.3725 | 1.436 |
| 28.4224 | 1.308 |
| 28.4723 | 1.602 |
| 28.5223 | 1.768 |
| 28.5722 | 1.289 |
| 28.6222 | 1.529 |
| 28.6721 | 1.381 |
| 28.722 | 1.381 |
| 28.772 | 1.289 |
| 28.8219 | 1.529 |
| 28.8719 | 1.344 |
| 28.9218 | 1.105 |
| 28.9718 | 1.344 |
| 29.0217 | 1.381 |
| 29.0716 | 1.529 |
| 29.1216 | 1.179 |
| 29.1715 | 1.363 |
| 29.2215 | 1.16 |
| 29.2714 | 1.031 |
| 29.3213 | 1.344 |
| 29.3713 | 1.215 |
| 29.4212 | 1.344 |
| 29.4712 | 1.344 |
| 29.5211 | 0.737 |
| 29.571 | 1.252 |
| 29.621 | 1.308 |
| 29.6709 | 1.657 |
| 29.7209 | 1.289 |
| 29.7708 | 1.381 |
| 29.8208 | 1.197 |
| 29.8707 | 1.215 |
| 29.9206 | 1.289 |
| 29.9706 | 1.013 |
| 30.0205 | 1.234 |
| 30.0705 | 1.381 |
| 30.1204 | 1.418 |
| 30.1703 | 0.994 |
| 30.2203 | 1.381 |
| 30.2702 | 1.215 |
| 30.3202 | 1.308 |
| 30.3701 | 0.976 |
| 30.42 | 1.234 |
| 30.47 | 1.308 |
| 30.5199 | 1.234 |
| 30.5699 | 1.381 |
| 30.6198 | 1.344 |
| 30.6698 | 1.436 |
| 30.7197 | 1.271 |
| 30.7696 | 1.492 |
| 30.8196 | 1.621 |
| 30.8695 | 1.75 |
| 30.9195 | 1.805 |
| 30.9694 | 1.657 |
| 31.0193 | 1.639 |
| 31.0693 | 1.565 |
| 31.1192 | 1.326 |
| 31.1692 | 1.326 |
| 31.2191 | 1.344 |
| 31.269 | 1.308 |
| 31.319 | 1.179 |
| 31.3689 | 1.436 |
| 31.4189 | 1.676 |
| 31.4688 | 1.51 |
| 31.5188 | 1.252 |
| 31.5687 | 1.234 |
| 31.6186 | 1.068 |
| 31.6686 | 1.234 |
| 31.7185 | 1.51 |
| 31.7685 | 1.639 |
| 31.8184 | 1.363 |
| 31.8683 | 1.197 |
| 31.9183 | 1.436 |
| 31.9682 | 1.455 |
| 32.0182 | 1.51 |
| 32.0681 | 2.081 |
| 32.118 | 3.02 |
| 32.168 | 5.599 |
| 32.2179 | 8.435 |
| 32.2679 | 7.735 |
| 32.3178 | 6.133 |
| 32.3678 | 5.654 |
| 32.4177 | 8.066 |
| 32.4676 | 20.534 |
| 32.5176 | 50.663 |
| 32.5675 | 45.12 |
| 32.6175 | 32.357 |
| 32.6674 | 15.212 |
| 32.7173 | 8.729 |
| 32.7673 | 7.109 |
| 32.8172 | 5.138 |
| 32.8672 | 4.42 |
| 32.9171 | 2.836 |
| 32.967 | 2.32 |
| 33.017 | 2.007 |
| 33.0669 | 1.602 |
| 33.1169 | 1.455 |
| 33.1668 | 1.326 |
| 33.2168 | 1.326 |
| 33.2667 | 1.197 |
| 33.3166 | 1.197 |
| 33.3666 | 1.234 |
| 33.4165 | 1.436 |
| 33.4665 | 1.308 |
| 33.5164 | 1.05 |
| 33.5663 | 1.068 |
| 33.6163 | 0.976 |
| 33.6662 | 1.289 |
| 33.7162 | 1.123 |
| 33.7661 | 0.994 |
| 33.816 | 0.958 |
| 33.866 | 1.252 |
| 33.9159 | 1.068 |
| 33.9659 | 1.068 |
| 34.0158 | 1.197 |
| 34.0658 | 1.179 |
| 34.1157 | 1.105 |
| 34.1656 | 1.087 |
| 34.2156 | 1.05 |
| 34.2655 | 1.087 |
| 34.3155 | 0.976 |
| 34.3654 | 0.958 |
| 34.4153 | 1.068 |
| 34.4653 | 0.921 |
| 34.5152 | 0.921 |
| 34.5652 | 0.81 |
| 34.6151 | 0.958 |
| 34.665 | 0.829 |
| 34.715 | 1.492 |
| 34.7649 | 1.547 |
| 34.8149 | 1.51 |
| 34.8648 | 1.694 |
| 34.9148 | 1.86 |
| 34.9647 | 2.947 |
| 35.0146 | 3.665 |
| 35.0646 | 7.735 |
| 35.1145 | 22.707 |
| 35.1645 | 26.022 |
| 35.2144 | 17.956 |
| 35.2643 | 12.081 |
| 35.3143 | 3.517 |
| 35.3642 | 1.823 |
| 35.4142 | 1.4 |
| 35.4641 | 1.197 |
| 35.514 | 1.068 |
| 35.564 | 1.179 |
| 35.6139 | 1.4 |
| 35.6639 | 1.473 |
| 35.7138 | 2.744 |
| 35.7638 | 2.541 |
| 35.8137 | 2.32 |
| 35.8636 | 1.234 |
| 35.9136 | 1.123 |
| 35.9635 | 0.939 |
| 36.0135 | 0.792 |
| 36.0634 | 0.902 |
| 36.1133 | 0.976 |
| 36.1633 | 0.755 |
| 36.2132 | 1.252 |
| 36.2632 | 1.252 |
| 36.3131 | 1.897 |
| 36.363 | 1.952 |
| 36.413 | 1.602 |
| 36.4629 | 1.289 |
| 36.5129 | 1.234 |
| 36.5628 | 1.565 |
| 36.6128 | 1.805 |
| 36.6627 | 1.455 |
| 36.7126 | 1.713 |
| 36.7626 | 1.197 |
| 36.8125 | 1.087 |
| 36.8625 | 0.958 |
| 36.9124 | 1.75 |
| 36.9623 | 1.657 |
| 37.0123 | 1.363 |
| 37.0622 | 1.087 |
| 37.1122 | 0.737 |
| 37.1621 | 0.847 |
| 37.212 | 0.792 |
| 37.262 | 0.626 |
| 37.3119 | 0.663 |
| 37.3619 | 0.718 |
| 37.4118 | 0.866 |
| 37.4618 | 0.718 |
| 37.5117 | 0.994 |
| 37.5616 | 0.737 |
| 37.6116 | 0.939 |
| 37.6615 | 0.939 |
| 37.7115 | 0.755 |
| 37.7614 | 0.939 |
| 37.8113 | 1.344 |
| 37.8613 | 0.884 |
| 37.9112 | 0.902 |
| 37.9612 | 1.013 |
| 38.0111 | 1.123 |
| 38.061 | 0.847 |
| 38.111 | 0.681 |
| 38.1609 | 0.847 |
| 38.2109 | 0.589 |
| 38.2608 | 0.792 |
| 38.3108 | 0.737 |
| 38.3607 | 0.663 |
| 38.4106 | 0.958 |
| 38.4606 | 0.681 |
| 38.5105 | 0.847 |
| 38.5605 | 1.16 |
| 38.6104 | 0.866 |
| 38.6603 | 0.921 |
| 38.7103 | 0.81 |
| 38.7602 | 1.031 |
| 38.8102 | 1.381 |
| 38.8601 | 1.123 |
| 38.91 | 1.05 |
| 38.96 | 0.755 |
| 39.0099 | 0.921 |
| 39.0599 | 0.792 |
| 39.1098 | 0.663 |
| 39.1598 | 0.81 |
| 39.2097 | 0.884 |
| 39.2596 | 0.663 |
| 39.3096 | 0.792 |
| 39.3595 | 0.847 |
| 39.4095 | 0.663 |
| 39.4594 | 0.755 |
| 39.5093 | 0.608 |
| 39.5593 | 0.663 |
| 39.6092 | 0.663 |
| 39.6592 | 0.976 |
| 39.7091 | 0.773 |
| 39.759 | 0.866 |
| 39.809 | 0.829 |
| 39.8589 | 1.105 |
| 39.9089 | 1.142 |
| 39.9588 | 1.252 |
| 40.0088 | 1.86 |
| 40.0587 | 2.376 |
| 40.1086 | 3.407 |
| 40.1586 | 5.101 |
| 40.2085 | 15.175 |
| 40.2585 | 33.112 |
| 40.3084 | 23.352 |
| 40.3583 | 19.521 |
| 40.4083 | 12.91 |
| 40.4582 | 4.144 |
| 40.5082 | 2.652 |
| 40.5581 | 2.063 |
| 40.608 | 1.289 |
| 40.658 | 1.271 |
| 40.7079 | 1.16 |
| 40.7579 | 1.013 |
| 40.8078 | 1.436 |
| 40.8578 | 1.86 |
| 40.9077 | 3.039 |
| 40.9576 | 2.063 |
| 41.0076 | 1.842 |
| 41.0575 | 1.547 |
| 41.1075 | 0.976 |
| 41.1574 | 0.976 |
| 41.2073 | 0.773 |
| 41.2573 | 1.013 |
| 41.3072 | 0.681 |
| 41.3572 | 0.773 |
| 41.4071 | 0.939 |
| 41.457 | 0.884 |
| 41.507 | 0.847 |
| 41.5569 | 0.792 |
| 41.6069 | 0.718 |
| 41.6568 | 0.534 |
| 41.7067 | 0.645 |
| 41.7567 | 0.737 |
| 41.8066 | 0.755 |
| 41.8566 | 0.663 |
| 41.9065 | 0.571 |
| 41.9565 | 0.7 |
| 42.0064 | 0.46 |
| 42.0563 | 0.718 |
| 42.1063 | 0.46 |
| 42.1562 | 0.792 |
| 42.2062 | 0.663 |
| 42.2561 | 0.681 |
| 42.306 | 0.663 |
| 42.356 | 0.773 |
| 42.4059 | 0.792 |
| 42.4559 | 1.805 |
| 42.5058 | 2.947 |
| 42.5557 | 2.486 |
| 42.6057 | 2.044 |
| 42.6556 | 1.805 |
| 42.7056 | 1.289 |
| 42.7555 | 0.608 |
| 42.8055 | 0.737 |
| 42.8554 | 0.608 |
| 42.9053 | 1.013 |
| 42.9553 | 0.866 |
| 43.0052 | 0.829 |
| 43.0552 | 0.847 |
| 43.1051 | 0.608 |
| 43.155 | 0.442 |
| 43.205 | 0.608 |
| 43.2549 | 0.442 |
| 43.3049 | 0.626 |
| 43.3548 | 0.497 |
| 43.4047 | 0.516 |
| 43.4547 | 0.589 |
| 43.5046 | 0.516 |
| 43.5546 | 0.571 |
| 43.6045 | 0.626 |
| 43.6545 | 0.792 |
| 43.7044 | 0.737 |
| 43.7543 | 0.589 |
| 43.8043 | 0.792 |
| 43.8542 | 0.571 |
| 43.9042 | 0.773 |
| 43.9541 | 0.663 |
| 44.004 | 0.368 |
| 44.054 | 0.589 |
| 44.1039 | 0.626 |
| 44.1539 | 0.626 |
| 44.2038 | 0.663 |
| 44.2537 | 0.46 |
| 44.3037 | 0.516 |
| 44.3536 | 0.571 |
| 44.4036 | 0.516 |
| 44.4535 | 0.405 |
| 44.5035 | 0.497 |
| 44.5534 | 0.589 |
| 44.6033 | 0.497 |
| 44.6533 | 0.626 |
| 44.7032 | 0.479 |
| 44.7532 | 0.405 |
| 44.8031 | 0.479 |
| 44.853 | 0.497 |
| 44.903 | 0.46 |
| 44.9529 | 0.681 |
| 45.0029 | 0.608 |
| 45.0528 | 0.516 |
| 45.1027 | 0.589 |
| 45.1527 | 0.552 |
| 45.2026 | 0.589 |
| 45.2526 | 0.516 |
| 45.3025 | 0.46 |
| 45.3525 | 0.405 |
| 45.4024 | 0.534 |
| 45.4523 | 0.516 |
| 45.5023 | 0.681 |
| 45.5522 | 0.921 |
| 45.6022 | 0.939 |
| 45.6521 | 1.16 |
| 45.702 | 1.326 |
| 45.752 | 1.271 |
| 45.8019 | 1.547 |
| 45.8519 | 1.694 |
| 45.9018 | 2.044 |
| 45.9517 | 1.529 |
| 46.0017 | 1.05 |
| 46.0516 | 0.81 |
| 46.1016 | 0.626 |
| 46.1515 | 0.645 |
| 46.2015 | 0.571 |
| 46.2514 | 0.681 |
| 46.3013 | 0.534 |
| 46.3513 | 0.589 |
| 46.4012 | 0.718 |
| 46.4512 | 0.497 |
| 46.5011 | 0.405 |
| 46.551 | 0.46 |
| 46.601 | 0.387 |
| 46.6509 | 0.46 |
| 46.7009 | 0.866 |
| 46.7508 | 0.663 |
| 46.8007 | 0.442 |
| 46.8507 | 0.479 |
| 46.9006 | 0.552 |
| 46.9506 | 0.534 |
| 47.0005 | 0.902 |
| 47.0505 | 1.252 |
| 47.1004 | 2.247 |
| 47.1503 | 1.934 |
| 47.2003 | 1.934 |
| 47.2502 | 1.473 |
| 47.3002 | 1.16 |
| 47.3501 | 0.866 |
| 47.4 | 0.755 |
| 47.45 | 0.516 |
| 47.4999 | 0.479 |
| 47.5499 | 0.497 |
| 47.5998 | 0.626 |
| 47.6497 | 0.608 |
| 47.6997 | 0.737 |
| 47.7496 | 0.424 |
| 47.7996 | 0.46 |
| 47.8495 | 0.645 |
| 47.8995 | 0.497 |
| 47.9494 | 0.405 |
| 47.9993 | 0.534 |
| 48.0493 | 0.589 |
| 48.0992 | 0.7 |
| 48.1492 | 0.681 |
| 48.1991 | 0.81 |
| 48.249 | 1.455 |
| 48.299 | 2.744 |
| 48.3489 | 3.112 |
| 48.3989 | 2.947 |
| 48.4488 | 2.247 |
| 48.4987 | 2.099 |
| 48.5487 | 2.007 |
| 48.5986 | 2.431 |
| 48.6486 | 2.523 |
| 48.6985 | 6.556 |
| 48.7485 | 17.459 |
| 48.7984 | 18.158 |
| 48.8483 | 11.326 |
| 48.8983 | 12.836 |
| 48.9482 | 8.508 |
| 48.9982 | 3.61 |
| 49.0481 | 1.934 |
| 49.098 | 1.197 |
| 49.148 | 0.902 |
| 49.1979 | 0.884 |
| 49.2479 | 0.718 |
| 49.2978 | 0.958 |
| 49.3477 | 0.792 |
| 49.3977 | 0.442 |
| 49.4476 | 0.534 |
| 49.4976 | 0.479 |
| 49.5475 | 0.516 |
| 49.5975 | 0.552 |
| 49.6474 | 0.681 |
| 49.6973 | 0.516 |
| 49.7473 | 0.516 |
| 49.7972 | 0.626 |
| 49.8472 | 0.589 |
| 49.8971 | 0.497 |
| 49.947 | 0.755 |
| 49.997 | 1.492 |
| 50.0469 | 1.786 |
| 50.0969 | 1.031 |
| 50.1468 | 1.068 |
| 50.1967 | 0.866 |
| 50.2467 | 0.755 |
| 50.2966 | 0.497 |
| 50.3466 | 0.442 |
| 50.3965 | 0.516 |
| 50.4465 | 0.534 |
| 50.4964 | 0.589 |
| 50.5463 | 0.681 |
| 50.5963 | 0.571 |
| 50.6462 | 0.46 |
| 50.6962 | 0.479 |
| 50.7461 | 0.534 |
| 50.796 | 0.405 |
| 50.846 | 0.516 |
| 50.8959 | 0.46 |
| 50.9459 | 0.479 |
| 50.9958 | 0.552 |
| 51.0457 | 0.35 |
| 51.0957 | 0.295 |
| 51.1456 | 0.424 |
| 51.1956 | 0.46 |
| 51.2455 | 0.35 |
| 51.2955 | 0.405 |
| 51.3454 | 0.534 |
| 51.3953 | 0.608 |
| 51.4453 | 0.405 |
| 51.4952 | 0.479 |
| 51.5452 | 0.534 |
| 51.5951 | 0.552 |
| 51.645 | 0.534 |
| 51.695 | 0.571 |
| 51.7449 | 0.552 |
| 51.7949 | 0.902 |
| 51.8448 | 0.958 |
| 51.8947 | 1.031 |
| 51.9447 | 1.087 |
| 51.9946 | 1.16 |
| 52.0446 | 0.737 |
| 52.0945 | 0.939 |
| 52.1445 | 0.773 |
| 52.1944 | 0.681 |
| 52.2443 | 0.479 |
| 52.2943 | 0.608 |
| 52.3442 | 0.718 |
| 52.3942 | 0.663 |
| 52.4441 | 0.626 |
| 52.494 | 0.608 |
| 52.544 | 0.479 |
| 52.5939 | 0.479 |
| 52.6439 | 0.737 |
| 52.6938 | 0.718 |
| 52.7437 | 0.645 |
| 52.7937 | 0.902 |
| 52.8436 | 0.884 |
| 52.8936 | 1.252 |
| 52.9435 | 1.436 |
| 52.9935 | 2.118 |
| 53.0434 | 2.357 |
| 53.0933 | 3.536 |
| 53.1433 | 7.403 |
| 53.1932 | 20.184 |
| 53.2432 | 27.79 |
| 53.2931 | 15.064 |
| 53.343 | 14.199 |
| 53.393 | 14.457 |
| 53.4429 | 6.077 |
| 53.4929 | 2.744 |
| 53.5428 | 1.676 |
| 53.5927 | 1.234 |
| 53.6427 | 1.05 |
| 53.6926 | 1.087 |
| 53.7426 | 0.847 |
| 53.7925 | 0.939 |
| 53.8425 | 1.087 |
| 53.8924 | 1.878 |
| 53.9423 | 2.431 |
| 53.9923 | 1.142 |
| 54.0422 | 1.252 |
| 54.0922 | 1.565 |
| 54.1421 | 0.792 |
| 54.192 | 0.516 |
| 54.242 | 0.608 |
| 54.2919 | 0.645 |
| 54.3419 | 0.424 |
| 54.3918 | 0.516 |
| 54.4417 | 0.497 |
| 54.4917 | 0.571 |
| 54.5416 | 0.7 |
| 54.5916 | 0.589 |
| 54.6415 | 1.234 |
| 54.6915 | 1.179 |
| 54.7414 | 1.16 |
| 54.7913 | 1.234 |
| 54.8413 | 1.013 |
| 54.8912 | 0.681 |
| 54.9412 | 0.681 |
| 54.9911 | 0.571 |
| 55.041 | 0.663 |
| 55.091 | 0.46 |
| 55.1409 | 0.516 |
| 55.1909 | 0.608 |
| 55.2408 | 0.387 |
| 55.2907 | 0.608 |
| 55.3407 | 0.645 |
| 55.3906 | 0.7 |
| 55.4406 | 0.958 |
| 55.4905 | 1.492 |
| 55.5404 | 2.026 |
| 55.5904 | 1.031 |
| 55.6403 | 0.958 |
| 55.6903 | 1.234 |
| 55.7402 | 1.013 |
| 55.7902 | 0.589 |
| 55.8401 | 0.737 |
| 55.89 | 0.681 |
| 55.94 | 1.013 |
| 55.9899 | 1.326 |
| 56.0399 | 1.768 |
| 56.0898 | 1.123 |
| 56.1397 | 1.215 |
| 56.1897 | 1.326 |
| 56.2396 | 1.473 |
| 56.2896 | 1.289 |
| 56.3395 | 1.087 |
| 56.3894 | 1.197 |
| 56.4394 | 0.81 |
| 56.4893 | 2.063 |
| 56.5393 | 3.72 |
| 56.5892 | 4.825 |
| 56.6392 | 2.523 |
| 56.6891 | 2.744 |
| 56.739 | 2.965 |
| 56.789 | 2.855 |
| 56.8389 | 1.455 |
| 56.8889 | 1.271 |
| 56.9388 | 1.363 |
| 56.9887 | 1.013 |
| 57.0387 | 0.902 |
| 57.0886 | 0.571 |
| 57.1386 | 0.552 |
| 57.1885 | 0.829 |
| 57.2384 | 0.773 |
| 57.2884 | 0.424 |
| 57.3383 | 0.442 |
| 57.3883 | 0.516 |
| 57.4382 | 0.368 |
| 57.4882 | 0.331 |
| 57.5381 | 0.516 |
| 57.588 | 0.276 |
| 57.638 | 0.295 |
| 57.6879 | 0.331 |
| 57.7379 | 0.442 |
| 57.7878 | 0.295 |
| 57.8377 | 0.276 |
| 57.8877 | 0.46 |
| 57.9376 | 0.313 |
| 57.9876 | 0.497 |
| 58.0375 | 0.516 |
| 58.0874 | 0.626 |
| 58.1374 | 0.534 |
| 58.1873 | 0.534 |
| 58.2373 | 0.608 |
| 58.2872 | 0.497 |
| 58.3372 | 0.46 |
| 58.3871 | 0.534 |
| 58.437 | 0.792 |
| 58.487 | 0.884 |
| 58.5369 | 0.884 |
| 58.5869 | 0.81 |
| 58.6368 | 0.958 |
| 58.6867 | 0.755 |
| 58.7367 | 0.939 |
| 58.7866 | 1.068 |
| 58.8366 | 1.123 |
| 58.8865 | 0.921 |
| 58.9364 | 1.068 |
| 58.9864 | 1.252 |
| 59.0363 | 0.847 |
| 59.0863 | 0.755 |
| 59.1362 | 0.645 |
| 59.1862 | 0.737 |
| 59.2361 | 0.755 |
| 59.286 | 1.252 |
| 59.336 | 1.215 |
| 59.3859 | 2.026 |
| 59.4359 | 1.51 |
| 59.4858 | 1.473 |
| 59.5357 | 1.363 |
| 59.5857 | 1.123 |
| 59.6356 | 1.252 |
| 59.6856 | 0.884 |
| 59.7355 | 0.313 |
| 59.7854 | 0.46 |
| 59.8354 | 0.516 |
| 59.8853 | 0.737 |
| 59.9353 | 1.142 |
| 59.9852 | 1.344 |
| 60.0352 | 1.271 |
| 60.0851 | 1.344 |
| 60.135 | 0.792 |
| 60.185 | 1.289 |
| 60.2349 | 0.81 |
| 60.2849 | 0.792 |
| 60.3348 | 0.442 |
| 60.3847 | 0.331 |
| 60.4347 | 0.497 |
| 60.4846 | 0.497 |
| 60.5346 | 0.35 |
| 60.5845 | 0.571 |
| 60.6344 | 0.497 |
| 60.6844 | 0.35 |
| 60.7343 | 0.442 |
| 60.7843 | 0.589 |
| 60.8342 | 0.276 |
| 60.8842 | 0.497 |
| 60.9341 | 0.442 |
| 60.984 | 0.552 |
| 61.034 | 0.645 |
| 61.0839 | 0.405 |
| 61.1339 | 0.626 |
| 61.1838 | 0.829 |
| 61.2337 | 0.755 |
| 61.2837 | 0.921 |
| 61.3336 | 0.939 |
| 61.3836 | 1.326 |
| 61.4335 | 1.197 |
| 61.4834 | 1.75 |
| 61.5334 | 1.86 |
| 61.5833 | 3.039 |
| 61.6333 | 8.674 |
| 61.6832 | 14.972 |
| 61.7332 | 8.913 |
| 61.7831 | 6.059 |
| 61.833 | 8.508 |
| 61.883 | 7.624 |
| 61.9329 | 3.278 |
| 61.9829 | 2.044 |
| 62.0328 | 1.4 |
| 62.0827 | 1.16 |
| 62.1327 | 0.718 |
| 62.1826 | 0.866 |
| 62.2326 | 1.252 |
| 62.2825 | 0.902 |
| 62.3324 | 0.737 |
| 62.3824 | 0.626 |
| 62.4323 | 0.792 |
| 62.4823 | 0.847 |
| 62.5322 | 0.958 |
| 62.5822 | 0.589 |
| 62.6321 | 0.829 |
| 62.682 | 0.884 |
| 62.732 | 1.381 |
| 62.7819 | 1.639 |
| 62.8319 | 0.939 |
| 62.8818 | 0.884 |
| 62.9317 | 1.473 |
| 62.9817 | 1.529 |
| 63.0316 | 1.068 |
| 63.0816 | 1.271 |
| 63.1315 | 1.215 |
| 63.1814 | 2.192 |
| 63.2314 | 3.941 |
| 63.2813 | 10.718 |
| 63.3313 | 17.772 |
| 63.3812 | 10.166 |
| 63.4312 | 6.409 |
| 63.4811 | 8.95 |
| 63.531 | 8.95 |
| 63.581 | 3.444 |
| 63.6309 | 1.676 |
| 63.6809 | 1.492 |
| 63.7308 | 1.234 |
| 63.7807 | 0.773 |
| 63.8307 | 0.792 |
| 63.8806 | 0.479 |
| 63.9306 | 0.571 |
| 63.9805 | 0.424 |
| 64.0304 | 0.497 |
| 64.0804 | 0.571 |
| 64.1303 | 0.442 |
| 64.1803 | 0.442 |
| 64.2302 | 0.552 |
| 64.2802 | 0.479 |
| 64.3301 | 0.442 |
| 64.38 | 0.331 |
| 64.43 | 0.221 |
| 64.4799 | 0.497 |
| 64.5299 | 0.276 |
| 64.5798 | 0.295 |
| 64.6297 | 0.387 |
| 64.6797 | 0.571 |
| 64.7296 | 0.405 |
| 64.7796 | 0.552 |
| 64.8295 | 0.35 |
| 64.8794 | 0.626 |
| 64.9294 | 0.424 |
| 64.9793 | 0.497 |
| 65.0293 | 0.516 |
| 65.0792 | 0.552 |
| 65.1292 | 0.405 |
| 65.1791 | 1.05 |
| 65.229 | 0.902 |
| 65.279 | 0.81 |
| 65.3289 | 0.902 |
| 65.3789 | 0.866 |
| 65.4288 | 0.737 |
| 65.4787 | 0.534 |
| 65.5287 | 0.571 |
| 65.5786 | 0.589 |
| 65.6286 | 0.387 |
| 65.6785 | 0.239 |
| 65.7284 | 0.368 |
| 65.7784 | 0.368 |
| 65.8283 | 0.184 |
| 65.8783 | 0.331 |
| 65.9282 | 0.46 |
| 65.9782 | 0.387 |
| 66.0281 | 0.35 |
| 66.078 | 0.276 |
| 66.128 | 0.405 |
| 66.1779 | 0.239 |
| 66.2279 | 0.35 |
| 66.2778 | 0.184 |
| 66.3277 | 0.276 |
| 66.3777 | 0.442 |
| 66.4276 | 0.276 |
| 66.4776 | 0.368 |
| 66.5275 | 0.405 |
| 66.5774 | 0.368 |
| 66.6274 | 0.424 |
| 66.6773 | 0.516 |
| 66.7273 | 0.866 |
| 66.7772 | 1.087 |
| 66.8272 | 1.4 |
| 66.8771 | 0.81 |
| 66.927 | 0.645 |
| 66.977 | 0.994 |
| 67.0269 | 0.939 |
| 67.0769 | 0.479 |
| 67.1268 | 0.405 |
| 67.1767 | 0.35 |
| 67.2267 | 0.405 |
| 67.2766 | 0.331 |
| 67.3266 | 0.35 |
| 67.3765 | 0.46 |
| 67.4264 | 0.442 |
| 67.4764 | 0.516 |
| 67.5263 | 0.516 |
| 67.5763 | 0.534 |
| 67.6262 | 0.387 |
| 67.6762 | 0.497 |
| 67.7261 | 0.534 |
| 67.776 | 0.589 |
| 67.826 | 0.295 |
| 67.8759 | 0.552 |
| 67.9259 | 0.405 |
| 67.9758 | 0.387 |
| 68.0257 | 0.552 |
| 68.0757 | 0.35 |
| 68.1256 | 0.405 |
| 68.1756 | 0.35 |
| 68.2255 | 0.479 |
| 68.2754 | 0.405 |
| 68.3254 | 0.479 |
| 68.3753 | 0.479 |
| 68.4253 | 0.589 |
| 68.4752 | 0.829 |
| 68.5252 | 1.657 |
| 68.5751 | 1.694 |
| 68.625 | 0.976 |
| 68.675 | 1.031 |
| 68.7249 | 1.105 |
| 68.7749 | 1.013 |
| 68.8248 | 0.7 |
| 68.8747 | 0.737 |
| 68.9247 | 0.792 |
| 68.9746 | 0.829 |
| 69.0246 | 0.847 |
| 69.0745 | 0.958 |
| 69.1244 | 0.976 |
| 69.1744 | 0.847 |
| 69.2243 | 0.755 |
| 69.2743 | 0.718 |
| 69.3242 | 0.7 |
| 69.3742 | 0.7 |
| 69.4241 | 0.626 |
| 69.474 | 0.442 |
| 69.524 | 0.7 |
| 69.5739 | 0.46 |
| 69.6239 | 0.313 |
| 69.6738 | 0.387 |
| 69.7237 | 0.479 |
| 69.7737 | 0.331 |
| 69.8236 | 0.424 |
| 69.8736 | 0.313 |
| 69.9235 | 0.424 |
| 69.9734 | 0.442 |
| 70.0234 | 0.276 |
| 70.0733 | 0.35 |
| 70.1233 | 0.313 |
| 70.1732 | 0.313 |
| 70.2231 | 0.295 |
| 70.2731 | 0.313 |
| 70.323 | 0.534 |
| 70.373 | 0.589 |
| 70.4229 | 0.534 |
| 70.4729 | 0.792 |
| 70.5228 | 1.16 |
| 70.5727 | 1.731 |
| 70.6227 | 3.352 |
| 70.6726 | 4.77 |
| 70.7226 | 3.389 |
| 70.7725 | 2.026 |
| 70.8224 | 2.541 |
| 70.8724 | 2.781 |
| 70.9223 | 2.247 |
| 70.9723 | 1.123 |
| 71.0222 | 1.473 |
| 71.0721 | 2.099 |
| 71.1221 | 2.762 |
| 71.172 | 1.418 |
| 71.222 | 1.05 |
| 71.2719 | 1.308 |
| 71.3219 | 1.842 |
| 71.3718 | 1.289 |
| 71.4217 | 0.81 |
| 71.4717 | 0.737 |
| 71.5216 | 0.737 |
| 71.5716 | 0.497 |
| 71.6215 | 0.46 |
| 71.6714 | 0.368 |
| 71.7214 | 0.331 |
| 71.7713 | 0.313 |
| 71.8213 | 0.479 |
| 71.8712 | 0.239 |
| 71.9211 | 0.387 |
| 71.9711 | 0.35 |
| 72.021 | 0.35 |
| 72.071 | 0.313 |
| 72.1209 | 0.35 |
| 72.1709 | 0.571 |
| 72.2208 | 0.387 |
| 72.2707 | 0.276 |
| 72.3207 | 0.147 |
| 72.3706 | 0.405 |
| 72.4206 | 0.35 |
| 72.4705 | 0.313 |
| 72.5204 | 0.184 |
| 72.5704 | 0.35 |
| 72.6203 | 0.184 |
| 72.6703 | 0.368 |
| 72.7202 | 0.35 |
| 72.7701 | 0.442 |
| 72.8201 | 0.405 |
| 72.87 | 0.424 |
| 72.92 | 0.368 |
| 72.9699 | 0.424 |
| 73.0199 | 0.442 |
| 73.0698 | 0.258 |
| 73.1197 | 0.387 |
| 73.1697 | 0.313 |
| 73.2196 | 0.221 |
| 73.2696 | 0.387 |
| 73.3195 | 0.166 |
| 73.3694 | 0.276 |
| 73.4194 | 0.258 |
| 73.4693 | 0.129 |
| 73.5193 | 0.276 |
| 73.5692 | 0.258 |
| 73.6191 | 0.368 |
| 73.6691 | 0.258 |
| 73.719 | 0.313 |
| 73.769 | 0.239 |
| 73.8189 | 0.368 |
| 73.8689 | 0.239 |
| 73.9188 | 0.387 |
| 73.9687 | 0.276 |
| 74.0187 | 0.368 |
| 74.0686 | 0.405 |
| 74.1186 | 0.866 |
| 74.1685 | 1.492 |
| 74.2184 | 1.197 |
| 74.2684 | 0.663 |
| 74.3183 | 0.718 |
| 74.3683 | 0.829 |
| 74.4182 | 0.847 |
| 74.4681 | 0.571 |
| 74.5181 | 0.442 |
| 74.568 | 0.829 |
| 74.618 | 1.326 |
| 74.6679 | 2.136 |
| 74.7179 | 2.21 |
| 74.7678 | 1.473 |
| 74.8177 | 1.234 |
| 74.8677 | 1.087 |
| 74.9176 | 1.713 |
| 74.9676 | 1.363 |
| 75.0175 | 0.958 |
| 75.0674 | 0.681 |
| 75.1174 | 0.737 |
| 75.1673 | 0.552 |
| 75.2173 | 0.442 |
| 75.2672 | 0.516 |
| 75.3171 | 0.424 |
| 75.3671 | 0.424 |
| 75.417 | 0.35 |
| 75.467 | 0.203 |
| 75.5169 | 0.35 |
| 75.5669 | 0.295 |
| 75.6168 | 0.295 |
| 75.6667 | 0.184 |
| 75.7167 | 0.331 |
| 75.7666 | 0.368 |
| 75.8166 | 0.35 |
| 75.8665 | 0.331 |
| 75.9164 | 0.295 |
| 75.9664 | 0.405 |
| 76.0163 | 0.331 |
| 76.0663 | 0.626 |
| 76.1162 | 0.81 |
| 76.1661 | 0.737 |
| 76.2161 | 0.405 |
| 76.266 | 0.534 |
| 76.316 | 0.571 |
| 76.3659 | 0.368 |
| 76.4159 | 0.276 |
| 76.4658 | 0.387 |
| 76.5157 | 0.405 |
| 76.5657 | 0.276 |
| 76.6156 | 0.405 |
| 76.6656 | 0.46 |
| 76.7155 | 0.479 |
| 76.7654 | 0.773 |
| 76.8154 | 0.847 |
| 76.8653 | 0.46 |
| 76.9153 | 0.479 |
| 76.9652 | 0.589 |
| 77.0151 | 0.497 |
| 77.0651 | 0.479 |
| 77.115 | 0.368 |
| 77.165 | 0.313 |
| 77.2149 | 0.239 |
| 77.2649 | 0.11 |
| 77.3148 | 0.239 |
| 77.3647 | 0.258 |
| 77.4147 | 0.239 |
| 77.4646 | 0.35 |
| 77.5146 | 0.313 |
| 77.5645 | 0.424 |
| 77.6144 | 0.331 |
| 77.6644 | 0.387 |
| 77.7143 | 0.46 |
| 77.7643 | 0.405 |
| 77.8142 | 0.497 |
| 77.8641 | 0.681 |
| 77.9141 | 1.657 |
| 77.964 | 1.473 |
| 78.014 | 1.031 |
| 78.0639 | 0.718 |
| 78.1139 | 0.976 |
| 78.1638 | 1.436 |
| 78.2137 | 1.142 |
| 78.2637 | 0.958 |
| 78.3136 | 0.884 |
| 78.3636 | 0.792 |
| 78.4135 | 0.81 |
| 78.4634 | 0.589 |
| 78.5134 | 0.534 |
| 78.5633 | 0.7 |
| 78.6133 | 0.718 |
| 78.6632 | 1.271 |
| 78.7131 | 1.068 |
| 78.7631 | 0.534 |
| 78.813 | 0.663 |
| 78.863 | 0.737 |
| 78.9129 | 0.847 |
| 78.9629 | 0.516 |
| 79.0128 | 0.534 |
| 79.0627 | 0.571 |
| 79.1127 | 0.442 |
| 79.1626 | 0.46 |
| 79.2126 | 0.331 |
| 79.2625 | 0.552 |
| 79.3124 | 0.295 |
| 79.3624 | 0.534 |
| 79.4123 | 0.7 |
| 79.4623 | 0.663 |
| 79.5122 | 1.142 |
| 79.5621 | 1.878 |
| 79.6121 | 1.584 |
| 79.662 | 1.087 |
| 79.712 | 1.436 |
| 79.7619 | 1.676 |
| 79.8119 | 1.989 |
| 79.8618 | 0.921 |
| 79.9117 | 1.031 |
| 79.9617 | 0.792 |
| 80.0116 | 0.571 |
